# Histone H4 acetylation regulates behavioral inter-individual variability in zebrafish

**DOI:** 10.1186/s13059-018-1428-y

**Published:** 2018-04-25

**Authors:** Angel-Carlos Román, Julián Vicente-Page, Alfonso Pérez-Escudero, Jose M. Carvajal-González, Pedro M. Fernández-Salguero, Gonzalo G. de Polavieja

**Affiliations:** 1 Champalimaud Neuroscience Programme, Champalimaud Centre for the Unknown, Avenida Brasília s/n, 1400-038 Lisbon, Portugal; 2 Instituto Cajal, Consejo Superior de Investigaciones Científicas, Av. Doctor Arce, 37, 28002 Madrid, Spain; 3 Physics Department, MIT, Cambridge, Massachusetts USA; 4 Departamento de Bioquímica y Biología Molecular y Genética, Universidad de Extremadura, Av. de Elvas s/n, 06071 Badajoz, Spain

**Keywords:** Epigenetics, HDAC, YY1, Behavior, Inter-individual variability, Zebrafish

## Abstract

**Background:**

Animals can show very different behaviors even in isogenic populations, but the underlying mechanisms to generate this variability remain elusive. We use the zebrafish (*Danio rerio*) as a model to test the influence of histone modifications on behavior.

**Results:**

We find that laboratory and isogenic zebrafish larvae show consistent individual behaviors when swimming freely in identical wells or in reaction to stimuli. This behavioral inter-individual variability is reduced when we impair the histone deacetylation pathway. Individuals with high levels of histone H4 acetylation, and specifically H4K12, behave similarly to the average of the population, but those with low levels deviate from it. More precisely, we find a set of genomic regions whose histone H4 acetylation is reduced with the distance between the individual and the average population behavior. We find evidence that this modulation depends on a complex of Yin-yang 1 (YY1) and histone deacetylase 1 (HDAC1) that binds to and deacetylates these regions. These changes are not only maintained at the transcriptional level but also amplified, as most target regions are located near genes encoding transcription factors.

**Conclusions:**

We suggest that stochasticity in the histone deacetylation pathway participates in the generation of genetic-independent behavioral inter-individual variability.

**Electronic supplementary material:**

The online version of this article (10.1186/s13059-018-1428-y) contains supplementary material, which is available to authorized users.

## Background

Classically, the phenotypic diversity of a population is considered to be generated by the genetic differences between its members and the disparity of their environmental influences [1]. A simple prediction from this view alone would then be that isogenic populations would not show variability when the environment is constant. Nevertheless, a pioneering study showed that there was variability independent of genetic differences in some morphological traits in mice raised in identical environments [2]. In recent years, similar results have been obtained for behavioral variability in mice and flies [3, 4]. Several mechanisms might contribute to this effect, including developmental noise [5], maternal and paternal effects [6], or the different experiences the individuals obtain by interacting with the environment or other animals [4], among others.

Our knowledge about behavioral variability independent of genetic differences has increased substantially, but its underlying mechanisms remain unclear. Neuronal changes such as neurogenesis or serotonin signaling have been shown to be final targets of behavioral individuality [3, 4], but the molecular mechanisms required to develop these differences are still unknown. Chromatin modifications could be relevant to encode stable differences among individuals and they have been hypothesized as a potential mechanism for the generation of experience-dependent behavioral individuality [4]. DNA methylation differences have been associated with behavioral castes in honeybees [7], and they are necessary and sufficient to mediate social defeat stress [8]. Histone acetylation is another of the main epigenetic modifications [9] and it has been shown to regulate different behaviors such as mating preference in prairie voles [10] or cast-mediated division of labor in ants [11]. We thus reasoned that molecular mechanisms linked to epigenetic modifications could lead to behavioral inter-individual variability.

We used zebrafish from 5 to 8 days post-fertilization (dpf) to dissect the molecular substrates of behavioral inter-individual variability. Laboratory zebrafish larvae show individuality in behavior [12] and they present some advantages, such as the wide range of genomic information, the simplicity of its pharmacological treatments, and the possibility to do large-scale behavioral analysis. Additionally, it is relevant to use a species in which we can observe directly developmental changes, as differences in behavioral individuality are likely accumulated during development [13]. Here we established zebrafish larvae as a model for the analysis of inter-individual variability in free-swimming behavior. In our experimental tests, we found that behavioral inter-individual variability of zebrafish larvae is independent of the genetic differences but it is correlated to histone H4 acetylation levels in a specific set of genomic sequences and regulated by a molecular complex composed by at least YY1 and HDAC1.

## Results

### Behavioral inter-individual variability in larval zebrafish is stable for days

**Fig. 1:**
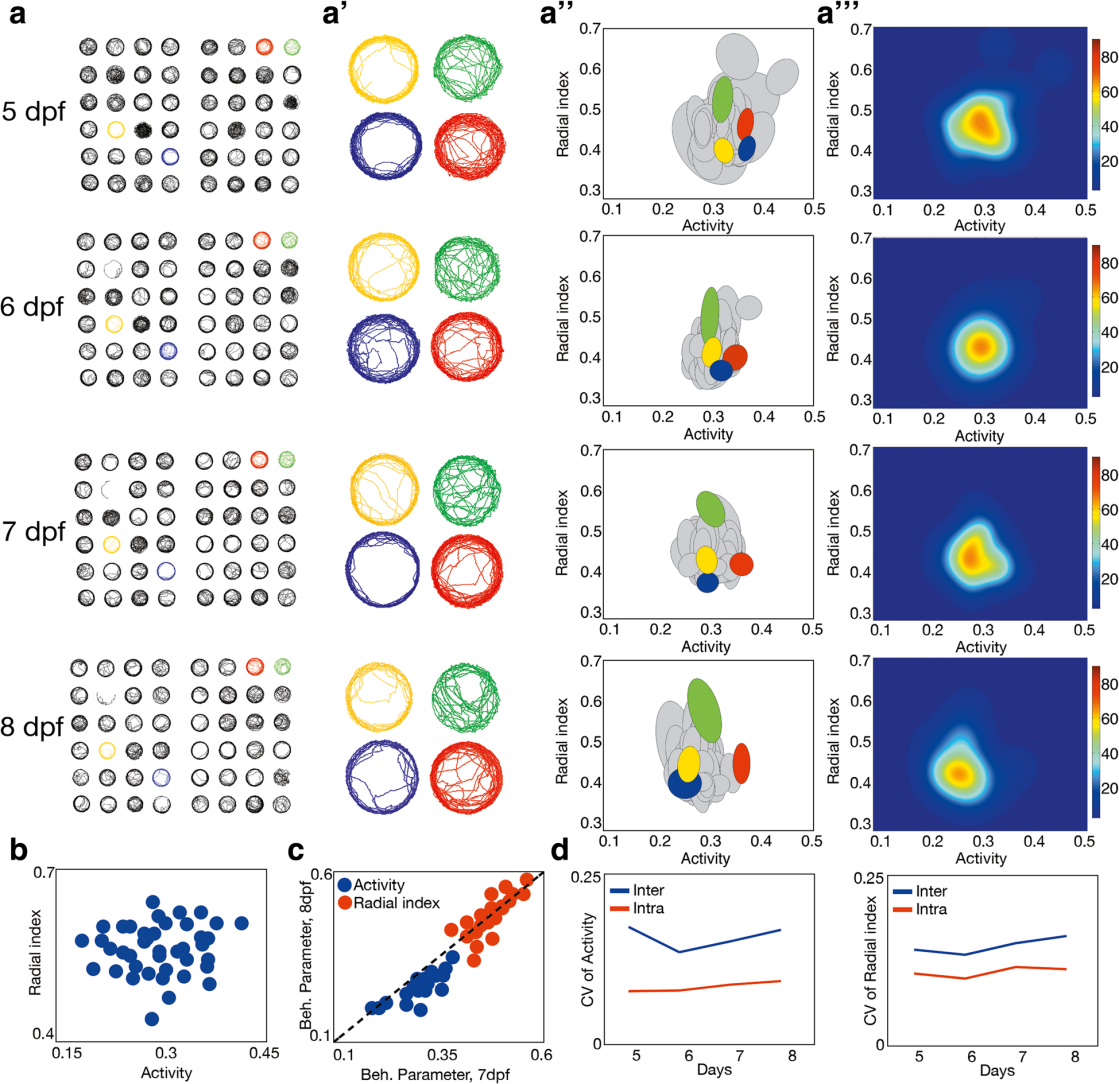
Behavioral inter-individual variability in a population of 48 larval zebrafish. **a** Example 20-min trajectories for the same larval group recorded at 5–8 dpf. **a’** Trajectories from four specific larvae zoomed from A. **a”** Population variability in activity and radial index of the same group at 5–8 dpf. Each *ellipse* represents the behavioral intra-individual variability for each single fish as described in “Methods”. Colors as in **a** and **a’**. **a”’** Probability density of finding an individual with a given mean activity and radial index at 5–8 dpf. **b** Radial index vs activity at 7 dpf of the same group. **c** Correlation of activity (*blue*) and radial index (*red*) between 7 and 8 dpf for the same group. **d** Median of intra-individual variability (*red*) and inter-individual variability (*blue*) for activity (*left*) and radial index (*right*) during the time course of the experiments

We used three steps to establish zebrafish larvae as a model to study behavioral inter-individual variability using a high-throughput setup (see “Methods” and Additional file 1: Figure S1 for the custom-built video tracking software, downloadable from www.multiwelltracker.es). We first determined that each larvae showed differences in their spontaneous behavior, as they can be observed by simple eye inspection of trajectories (Fig. 1a, a’ , from 5 to 8 dpf). We quantified this behavior by using eight parameters: overall activity (percentage of time in movement), radial index (average relative distance from the border towards the center of the well), bursting frequency (percentage of stop/move transitions), average speed (during activity), tortuosity (average turning angle during activity), circularity (percentage of the total movement within the border/center axis), average instant acceleration (in the stop/move transitions), and instant tortuosity (average turning angle in the stop/move transitions). As expected, some of these parameters were significantly correlated with one another. Specifically, bursting frequency, average speed, tortuosity, instant acceleration, and instant tortuosity correlated to overall activity (Additional file 1: Figure S2A–E, *P* < 0.009, two-sided *t*-test), while circularity correlated to radial index (Additional file 1: Figure S2F, *P* < 0.001, two-sided *t*-test). Nevertheless, activity and radial index were independent of each other (Fig. 1b, *P* = 0.98, two-sided *t*-test).

In a second step, we showed that individual differences in these eight parameters were robust over several days (Additional file 1: Figure S2G–J, R ≥ 0.38, *P* < 0.01 in two-sided *t*-tests for *R* = 0, 5 vs 6 dpf; Fig. 1c and Additional file 1: Figure S2K–M, R ≥ 0.55, *P* < 0.001 in two-sided *t*-tests for R = 0, 7 vs 8 dpf). Finally, the third step consisted in proving that inter-individual variability is larger than intra-individual variability (see “Methods”). While some parameters presented higher intra-individual variability (circularity and instant acceleration; Additional file 1: Figure S2N; *P* < 0.01, permutation tests), others showed either only slightly higher inter-individual variability (speed and instant tortuosity; Additional file 1: Figure S2O, P; *P* = 0.05 and *P* = 0.03, respectively, permutation tests) or stronger differences between inter- and intra-individual variability but with unstable variability levels across the days (bursting frequency and tortuosity; Additional file 1: Figure S2O, P; *P* < 0.01, permutation tests). In the case of activity and radial index, the inter-individual variability was consistently higher than intra-individual variability and the variability levels remained stable during the four days of the experiment (Fig. 1d, *P* < 0.01 permutation tests; see Additional file 1: Figure S2Q–T for the daily distributions of inter- and intra-individual variability of activity and radial index). This initial characterization of the parameters led us to focus on activity and radial index as the main parameters to describe variability in free-swimming behavior in larval zebrafish, although we cannot discard the additional role of the rest of the parameters during larval development.

Then, we performed several control experiments using activity and radial index in order to show that these parameters are not affected by technical artifacts in the setup (“Methods”; Additional file 1: Figure S3A–C). In addition, we tested if subtle developmental differences across the larvae could lead to differences in activity and radial index. Using larval size and *amigo1* gene expression as known reporters of developmental changes during zebrafish development [14, 15], we did not find any significant correlation between these reporters and the individual activity or radial index of the population at 7 dpf (Additional file 1: Figure S3D, E; *P* > 0.05 for all the comparisons, using two-sided *t*-tests). Finally, we showed that activity and radial index can describe inter-individual variability not only during free-swimming behavior but also in response to stimuli like light flashes, mechanical perturbation, or being in a novel tank (“Methods”; Additional file 1: Figure S3F).

We can display inter-individual and intra-individual variability of a population using the two-dimensional phenotypic space defined by activity and radial index (Fig. 1a”). The degree of inter-individual variability can then be visualized using the probability density of finding an individual in a population with a given mean activity and radial index (“Methods”; Fig. 1a”’). While this distribution gives a visual and intuitive characterization of behavioral variability, an even simpler characterization is achieved using, for each group, a single parameter summarizing its two-dimensional variability. We used generalized variance [16], computed as the determinant of the covariance matrix (Additional file 2: Table S1; see also “Methods”), as this single parameter for measuring dispersion in two dimensions. We then used this parameter to compare the behavioral inter-individual variability of two populations, but other parameters like the standard deviation for each parameter gave similar statistical results (Additional file 2: Table S2).

### Sources of behavioral inter-individual variability in zebrafish

**Fig. 2:**
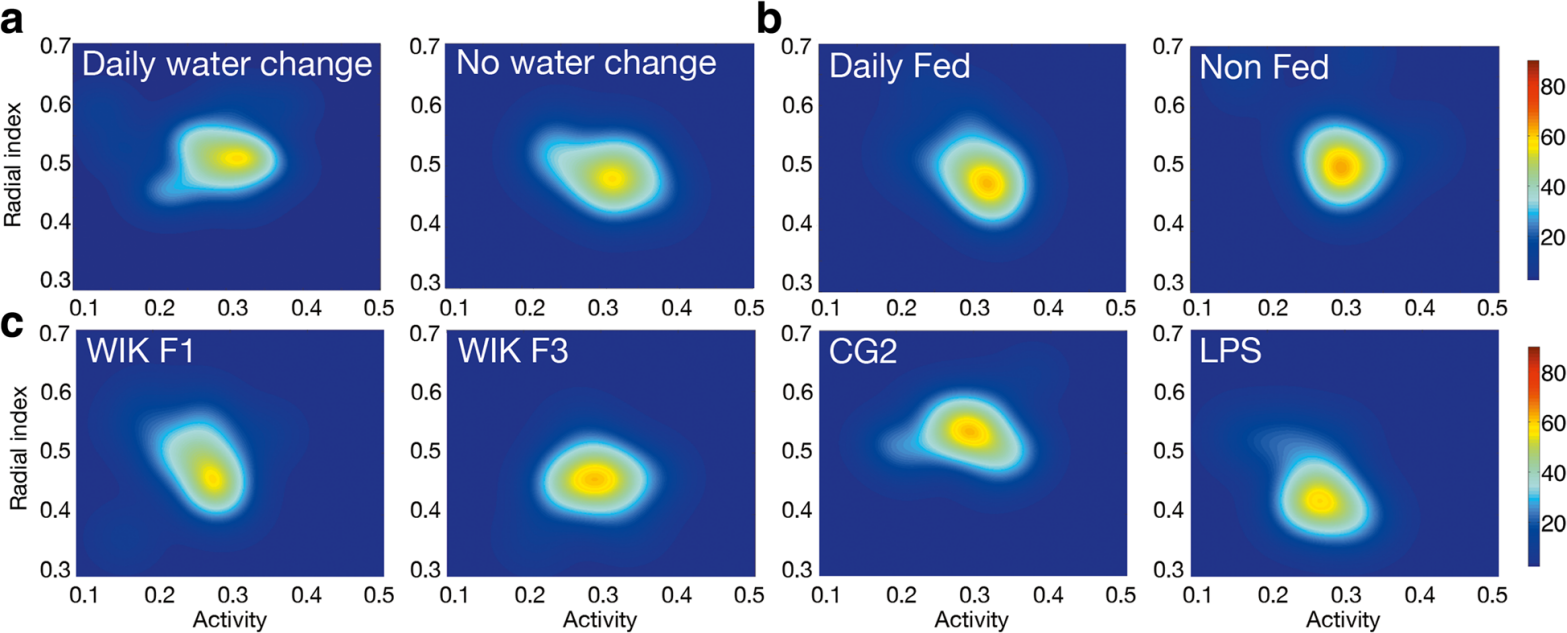
Impact of environmental changes and genetic background on behavioral inter-individual variability. **a** Probability density of finding an individual with a given mean activity and radial index for additional larval groups (24 individuals) with and without daily water changes, at 7 dpf. **b** Same as **a**, but for additional daily fed (control case) and non-fed animals throughout the experiment. **c** Same as **a** for additional groups with different genetic backgrounds: WIK F1 (three inbreeding cycles), WIK F3 (five inbreeding cycles), CG2 (gymnozygotic fish clones), and LPS (outbred parents)

Our setup allowed us to perform high-throughput tests to study the possible origins of behavioral inter-individual variability, which might depend on environmental manipulations and the genetic differences across the population. Our experiments minimized environmental influences by isolating eggs in plates at the pharyngula stage (24 hpf) and by keeping them at a controlled temperature (27–28 °C). Manual changes in water (24 h before the experiment) or feeding did not affect inter-individual variability as measured by generalized variance (Fig. 2a, *P* = 0.42 and Fig. 2b, *P* = 0.38, respectively; permutation tests).

We also found that behavioral variability of a population did not depend on the genetic variability of its individuals. Our control laboratory WIK zebrafish population (F1) resulted from a single batch of eggs retrieved from two adults with at least three cycles of inbreeding. We obtained the same behavioral inter-individual variability after two more inbreeding cycles (WIK F3, Fig. 2c, *P* = 0.33 permutation test) and in an isogenic population [17] (CG2, Fig. 2c, *P* = 0.44, permutation test). Also, we did not find changes in the behavioral inter-individual variability using groups of siblings from genetically diverse outbred parents (LPS line, Fig. 2c, *P* = 0.38, permutation test).

### Changes in the histone acetylation pathway alter behavioral inter-individual variability

**Fig. 3:**
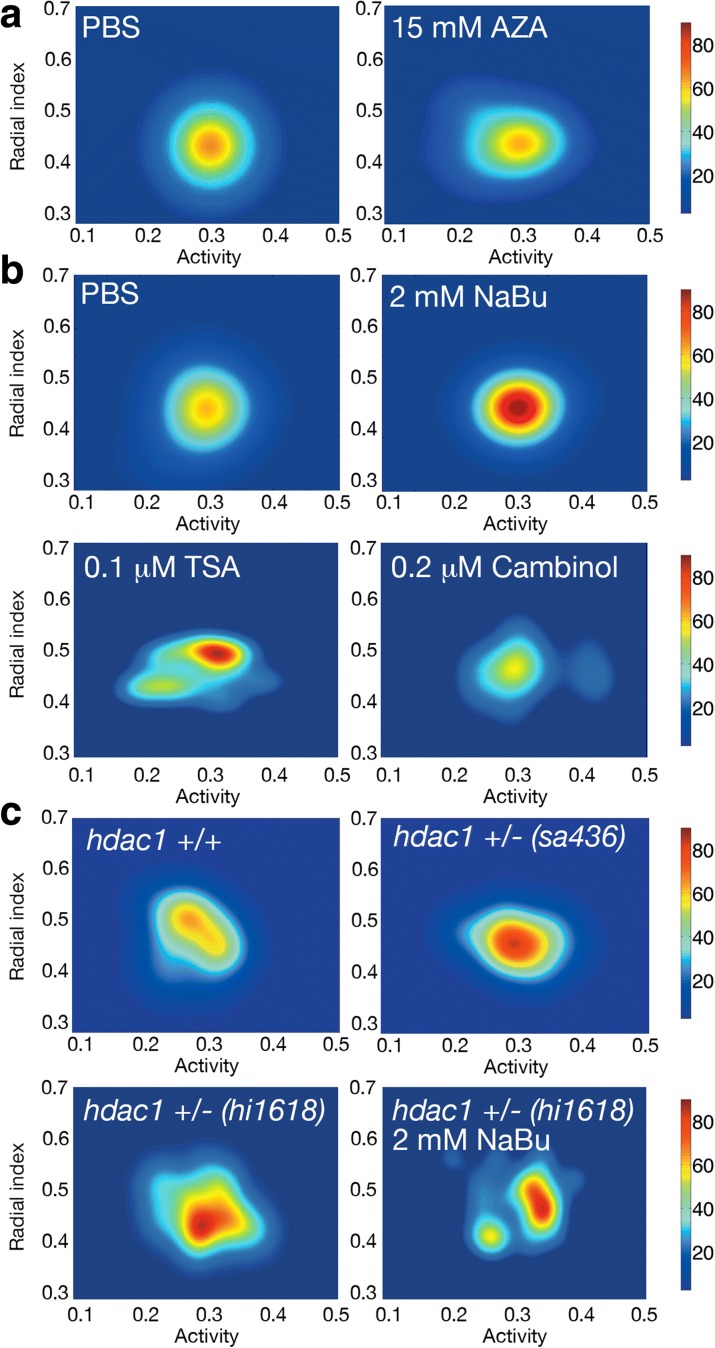
Epigenetic modulation of behavioral inter-individual variability. **a** Probability density map for 24 fish treated with a PBS solution as control and 15 mM AZA for 24 h. **b** The same for PBS (*top left*), 2 mM NaBu (*top right*), 0.1 μM trichostatin A (*TSA*, *bottom left*), and 0.2 μM cambinol (*bottom right*). **c** Probability density map for *hdac1 +/+* (*top left*), *hdac1 +/−* (*sa436* mutant, *top right*), *hdac1 +/−* (*hi1618* mutant, *bottom left*) and *hdac1 +/−* (*hi1618* mutant) with 2 mM NaBu for 24 h (*bottom right*) larvae

The absence of effects from genetic variability prompted us to test whether behavioral inter-individual variability could be modified by different epigenetic factors. To test the contribution of DNA methylation we used 5-azacytidine (AZA), an inhibitor of DNA methyltransferases [18]. We found that AZA added to the water did not alter the behavioral generalized variance of a population (15 mM AZA, Fig. 3a, *P* = 0.44, permutation test) even if it reduced 3-methyl DNA in larval zebrafish (Additional file 1: Figure S4A, *P* < 0.01 using a two-sided *t*-test). We then studied the role of histone deacetylation, a reversible molecular process in which an acetyl functional group is removed from specific residues of histones H3 and H4 [9]. This system is regulated by a group of enzymes called histone deacetylases (HDACs) that can be divided into three classes based on their sequence homology. Class I HDACs (HDAC1, HDAC3, and HDAC8 in the case of zebrafish) are strictly localized in the cell nucleus, and they are normally ubiquitously expressed, while class II HDACs shuttle from cytoplasm to nucleus, and each protein is specifically expressed in a few tissues. Class III enzymes are different from class I and II from a phylogenetic point of view; they are NAD^+^-dependent deacetylases and known as sirtuins [19]. To test the effect of HDACs on behavioral inter-individual variability, we first used sodium butyrate (NaBu; a class I HDAC inhibitor) at the standard concentration of 2 mM [20], and we confirmed that it increases the level of total acetyl-histone H4 (acH4) in larval zebrafish (Additional file 1: Figure S4B, *P* < 0.01 using a two-sided *t*-test, NaBu). We found that this treatment reduced the behavioral variability of a WIK F3 sibling population after 24 h as measured by generalized variance (2 mM NaBu, Fig. 3b, top right) compared to control PBS-treated larvae (PBS, Fig. 3b, top left, *P* < 0.001, permutation test). Note that this treatment only altered variability and not the mean of the population parameters (*P* = 0.63, permutation test). When we removed the NaBu from the water, behavioral variability was recovered after an additional 24 h (Additional file 1: Figure S4C, *P* = 0.71, permutation test). Similarly to the behavior, the total levels of acH4 increased with the treatment and recovered 24 h after removing the NaBu (Additional file 1: Figure S4B, *P* = 0.42 using a two-sided *t*-test, NaBu/PBS). In addition, there were no significant differences between the acH4 levels in WIK larvae from 5 to 9 dpf (Additional file 1: Figure S4B, *P* ≥ 0.51 for all comparisons using two-sided *t*-tests). Another HDAC inhibitor (against class I and class II HDACs) like Trichostatin A (0.1 μM TSA, Fig. 3b, bottom left) had a similar behavioral effect as NaBu, reducing the behavioral variability of the population (*P* = 0.02 permutation test) and increasing histone H4 acetylation (Additional file 1: Figure S4B, *P* < 0.01 using a two-sided *t*-test, TSA). In contrast, an inhibitor of class III HDACs like cambinol (0.2 μM cambinol, Fig. 3b, bottom right) did not alter the behavioral generalized variance of the population (*P* = 0.71, permutation test), even when cambinol treatment increased the acetylation levels of histone H4 (Additional file 1: Figure S4B, *P* < 0.01 using a two-sided *t*-test, Cmb). We confirmed the specificity of the effects on class I histone deacetylases by studying mutant larvae as an alternative to the use of drugs. We found that two different heterozygotic mutant populations of the class I histone deacetylase *hdac1* (*hdac1 +/−*), *sa436* [21] and *hi1618* [22], showed reduced behavioral inter-individual variability compared to their AB controls and an increase in histone H4 acetylation, mirroring the results obtained with the drugs (Fig. 3c, top and bottom left; *P* = 0.008 for *sa436* and *P* = 0.006 for *hi1618* permutation tests; Additional file 1: Figure S4B, *P* < 0.01 using two-sided *t*-tests, *hdac1 +/−*). Addition of 2 mM NaBu to the *hdac1 +/− hi1618* population did not change the behavioral inter-individual variability of the larvae (Fig. 3c, bottom right; *P* = 0.23 permutation test). As the NaBu treatment might inhibit the remaining HDAC1 activity in the heterozygotic larvae, we cannot discern if the drug was affecting variability through HDAC1 or if we reached a limit in the decrease of behavioral variability. The presence of a second side peak in the population might point to a differential NaBu effect depending on the reduced HDAC1 activity of each individual. These results suggest that the histone deacetylation pathway modulates the behavior of zebrafish larvae without affecting average behavior.

### Histone H4 acetylation levels correlate with behavioral distance to average behavior

**Fig. 4:**
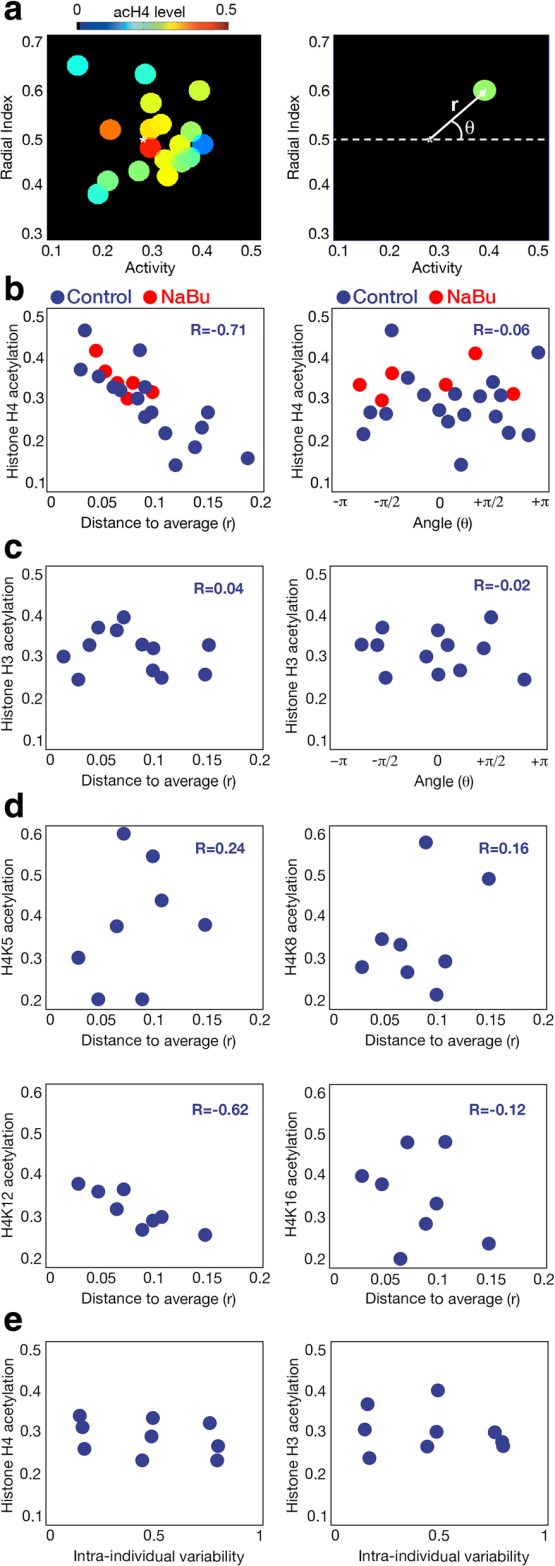
Relation between histone acetylation levels and behavior. **a** Average histone H4 acetylation levels of fish depending on their behavior using 90 fish as the initial population and clustering them into groups (*left*). The polar coordinate system as a transformation of the previous Cartesian system, and the two parameters used to analyze the dependence between histone H4 acetylation and behavior (*right*). **b** Relation between the values of histone H4 acetylation and the two parameters of the polar coordinate system centered on the average behavior of the population: the distance to the average (*left*) and the angle with the horizontal axis (*right*). *Blue dots* are control larvae while *red dots* indicate Nabu-treated animals. R coefficients for control samples are shown. **c** Same as **b**, but for histone H3 acetylation. **d** Relation between the values of histone H4K5 (*top left*), H4K8 (*top right*), H4K12 (*bottom left*), and H4K16 (*bottom right*) acetylation and the distance to the average behavior of the population. R coefficients are shown. **e** Relation between the values of histone H4 (*left*) and H3 (*right*) acetylation and clusters of larvae with similar intra-individual behavioral variability

We have shown that the degree of behavioral inter-individual variability of a population depends on its average acetylation levels. Since an increment in the global histone acetylation decreased this variability without changing the average behavior, we reasoned that the individuals with higher mean acetylation should be placed near the average population behavior in the phenotypic space. To test this hypothesis, we performed an experiment with 90 zebrafish individuals to obtain their histone H4 acetylation state depending on their distance to the average behavior of the population. As we needed at least five larvae in order to get enough tissue for the experiment, we pooled five larvae with very similar behavior and measured their acetylation state using ELISA kits that allow the quantification of histone H4 acetylation and total histone content. We found that pools of larvae whose behavior was placed near the average of the population had higher mean histone H4 acetylation values (Fig. 4a, left; *P* = 0.007 in a two-sided *t*-test). To quantify the dependence between the average histone H4 acetylation and the position in the phenotypic space of the samples, we first defined a polar coordinate system (centered on the average behavior of the population) and then obtained two magnitudes to characterize each pool of fish: their average distance to the center (*r*) and their average angle with the horizontal axis (*θ*) (Fig. 4a, right). We found that the histone H4 acetylation levels of the larval pools highly correlated with their phenotypic distance *r* to the average, while we found no correlation with their angular position *θ* (Fig. 4b, blue dots; *P* < 0.001 and *P* = 0.53, respectively, in two-sided *t*-tests for *R* = 0). We found similar correlations when we analyzed the distance to the mean of each behavioral parameter separately (Additional file 1: Figure S5A, *P* < 0.001 for both activity and radial index in two-sided *t*-tests for R = 0).

So far, we have shown that individuals with higher histone H4 acetylation levels display a behavior similar to the average of the population, while the variability of the population behavior increases at lower histone H4 acetylation levels. This is consistent with our previous experiments that reduced the behavioral variability of a population by increasing its acetylation levels using HDACi or *hdac1 +/−* mutant animals. In fact, when we used fish treated with NaBu to perform the same acH4 quantification, we found that their histone H4 acetylation is at a similar level to the non-treated individuals with highest histone H4 acetylation (Fig. 4b, red dots; *P* = 0.24, two-sided *t*-test). This shows that the NaBu-treated animals present histone H4 acetylation levels within the physiological range of the animals, consistent with NaBu having the global effect of increasing the histone H4 acetylation levels of the population by bringing them close to the animals with highest acetylation. Then we analyzed if the effect observed for histone H4 acetylation was also present for histone H3. Interestingly, histone H3 acetylation levels correlated with neither *r* nor *θ* (Fig. 4c, *P* = 0.61 and *P* = 0.64, respectively, in two-sided *t*-tests for R = 0). This result made us focus on the specific marks for histone H4 acetylation. We found that H4K12 acetylation levels correlated with *r*, while other marks were not affected by the behavioral position of the samples (Fig. 4d and Additional file 1: Figure S5B, *P* = 0.005 for H4K12, *P* > 0.27 for the rest of the marks in two-sided *t*-tests for R = 0). Our approach consisting of pooling larvae with a similar behavior is consistent, as we observed that different pools obtained from the same behavioral position maintain very similar acH4 and acH3 levels (Additional file 1: Figure S5C, left), and pools located very near one another in the behavioral space maintain very similar histone H4 acetylation levels compared to the rest of the clusters (Additional file 1: Figure S5C, right; *P* = 0.56, two-sided *t*-test).

Finally, we studied if intra-individual variability could also be linked to histone acetylation. We pooled samples with similar intra-individual variability and quantified histone H4 and H3 acetylation (Fig. 4e). We did not find a correlation between acetylation levels and behavioral intra-individual variability (*P* = 0.49 and *P* = 0.58 for histones H4 and H3, respectively, two-sided *t*-tests).

### Genomic regions linking histone H4 acetylation and behavioral inter-individual variability

**Fig. 5:**
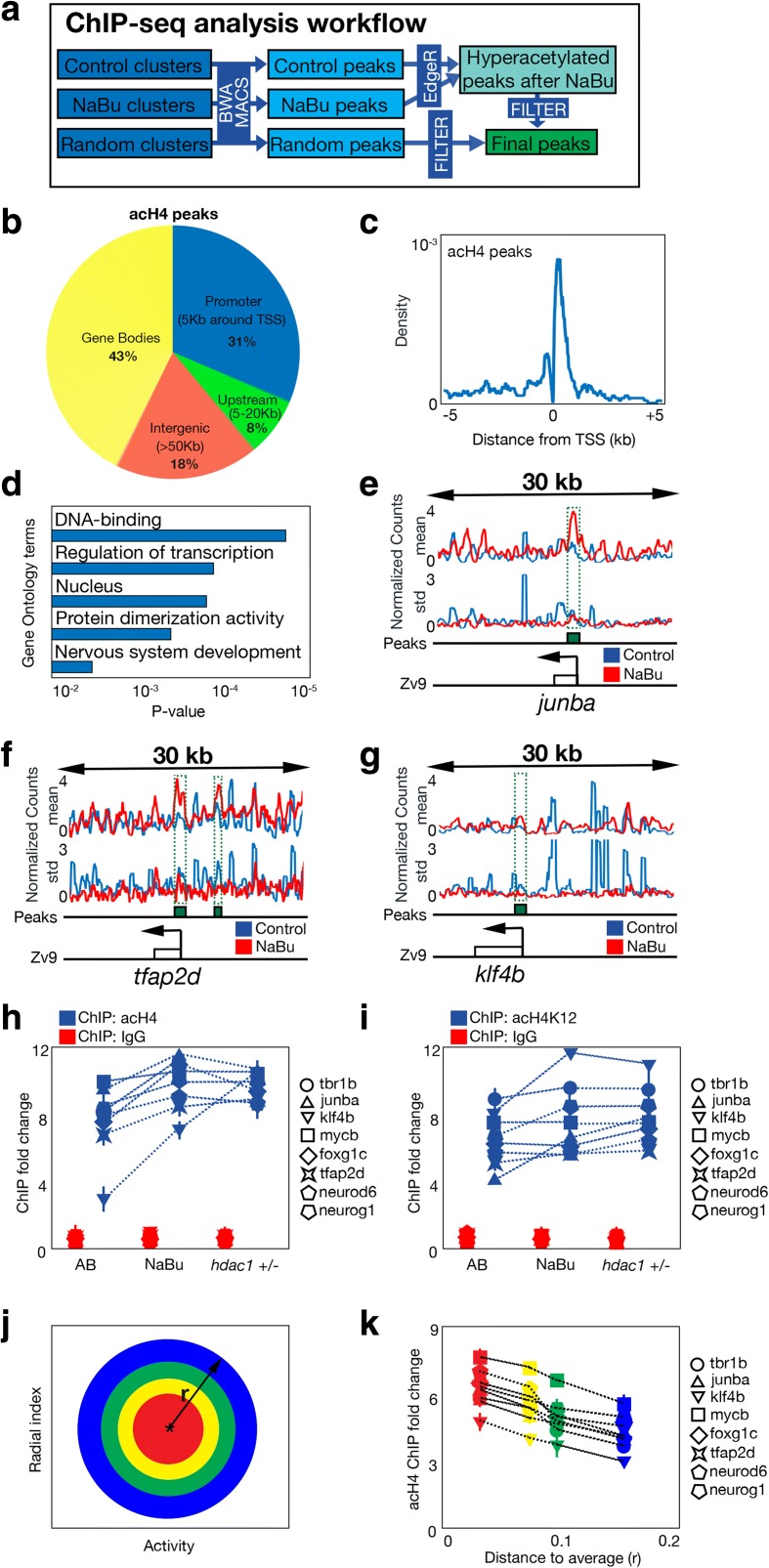
Histone 4 acetylation regions related to behavioral inter-individual variability. **a** Workflow of the analytical steps using histone H4 acetylation ChIP-seq. **b** Classification of the acH4 peaks obtained depending on their position relative to the transcription start site (*TSS*) of the nearest gene. **c** Histogram representing the relative positions of the acH4 peaks located around the TSS of the nearest gene. **d** Enriched GO terms of the acH4 peaks. **e** Snapshot of the raw reads results obtained in the acH4 ChIP-seq in the 30-kb region around *junba* (marked with a *box* and an *arrow* showing its TSS). At the top, *blue lines* indicate the mean reads in the control samples, while *red lines* indicate the mean reads in the NaBu-treated samples. At the *bottom*, the *lines* indicate the standard deviation of control and Nabu-treated samples across the region. *Green box* indicates the peaks detected by the algorithm. **f** Same as **e** but for the *tfap2d* gene. **g** Same as **e** but for the *klf4b* gene. **h** Histone H4 acetylation levels quantified in conventional ChIP as the fold change compared to the non-bound fraction in eight selected regions in control, NaBu-treated, and *hdac1 +/−* larvae. *Blue symbols* represent the acH4 ChIP, while *red symbols* show a control ChIP using only IgG. *Bars* represent standard deviation using three replicates. The legend on the *right* indicates the names of the regions. **i** Same as **h** but using H4K12 acetylation ChIPs. **j** Diagram representing cluster selection in **k**. Each color marks the behavioral area from which we retrieve larvae for comparison. This area depends on the distance to the average behavior of the population (*r*). **k** Same as **h** but comparing the acH4 differences in the different clusters represented in **j**

Our results link histone H4 acetylation level of the individuals to their behavior. However, fish with similar (low) histone H4 acetylation levels also can show very different behaviors, so other factors must contribute to behavioral inter-individual variability. We hypothesized that these factors could be the acetylation differences in specific genomic regions associated with behavior. To explore this possibility, we compared the histone H4 acetylation levels between two groups of zebrafish, one with high and the other with low behavioral variability. For the first population (control), we used four samples of five pooled sibling fish. The larvae within each sample had similar behaviors, and each sample behaved differently to the others (see “Methods” for details). For the second population (NaBu), we used four samples of five sibling fish treated with NaBu. As in the first case, the larvae from each sample had very similar behavioral parameters. Nevertheless, as the NaBu population had reduced behavioral inter-individual variability (Fig. 3b), the behavioral differences between the groups were low. We then retrieved the acH4 epigenomic profiles of the samples in each group using ChIP-seq and calculated the peaks for each sample with the MACS algorithms. Then, we computed the histone H4 acetylation differences for each peak between control and NaBu populations using standard techniques adopted from gene expression analysis as EdgeR (see Fig. 5a and “Methods” for details). We selected the candidate peaks that had significantly higher histone H4 acetylation in the NaBu population (*P* < 0.01, exact test for quantile-adjusted conditional maximum likelihood) compared to the control population. Following our hypothesis, the histone H4 acetylation variability within these peaks across the population might be responsible for the behavioral inter-individual variation. To test this idea, we compared the acetylation variability across the four control samples observed in these candidate peaks with the results of a novel ChIP-seq experiment. In this case, we selected four samples of five randomly selected pooled fish. Thus, the histone H4 acetylation variability across these samples should be reduced, as it will not be associated with different behaviors between the samples. Supporting our hypothesis, most of the candidate peaks (95%) showed less variability in the random samples than in the control samples (Additional file 1: Figure S6A). Still, we then excluded the candidate regions whose acH4 variability was not associated with behavior. From this procedure we obtained a final set of 729 regions in which NaBu increased histone H4 acetylation potentially responsible for the behavioral inter-individual variability differences observed after alteration of the HDAC pathway (Additional file 2: Table S3).

We studied the relative genomic positions of these peaks (Fig. 5b); the peaks that were hyperacetylated after NaBu were enriched in promoter regions (± 5 kb around the transcription start site (TSS)) and gene bodies (31% and 43%, respectively). Upstream regions (from 5 to 20 kb of the TSS) accounted for 8% of the total peaks, while intergenic regions accounted for 18%. This distribution is not very different to others obtained for acetyl-histone marks in other conditions [23]. We then studied the subset of peaks located near the TSS, as the histone H4 acetylation changes detected in these regions could be associated with differences in the expression of nearby >genes. We observed that these peaks were located very near to the TSS, but excluded from the exact TSS (Fig. 5c), a typical acH4 effect seen in previous work [23]. We then characterized this set of regions located near TSSs using Gene Ontology (GO) terms associated with their neighboring genes (Fig. 5d). We found five enriched terms (*P* < 0.01, Fisher’s exact test); the highest was related to transcription factor activity while the others were related to neural development. For subsequent analyses, and in order to assess their role in the control of behavioral inter-individual variability, we selected eight of the candidate peaks that were located near the TSS of transcription factors that played an important role for neural development (*tbr1b*, *junba*, *klf4b*, *mycb*, *foxg1c*, *tfpa2d*, *neurod6*, *neurog1*; see Fig. 5e-g for ChIP-seq snapshots of the normalized levels of acH4 around these regions) [24–28]. We considered that these transcription factors might be involved in major transcriptional changes that resulted in inter-individual behavioral differences.

We analyzed the levels of histone H4 acetylation in these eight regions by conventional ChIP for three different conditions: AB populations, NaBu-treated animals, and *hdac1 +/−* populations (Fig. 5h). We observed that the acH4 content in these regions was increased not only after NaBu treatment (*P* < 0.01 for all the regions, two-sided *t*-tests), as predicted by the ChIP-seq results, but also in *hdac1 +/−* populations (*P* < 0.01 for all the regions, two-sided *t*-tests). Acetylation of the specific H4K12 mark in these regions did not reflect exactly the same results of global acH4 (Fig. 5i), as five regions increased their H4K12 acetylation in either NaBu-treated or *hdac1 +/−* populations (*tfap2d*, *junba*, *klf4b*, *neurog1* and *tbr1b*, *P* < 0.03, two-sided *t*-tests), while two of them only increased H4K12 acetylation in *hdac1 +/−* larvae (*foxg1c*, *neurod6*, *P* < 0.02, two-sided *t*-tests) and another one did not respond at all (*mycb*). These results suggest that the effect of NaBu was mediated by the inhibition of HDAC1 deacetylation of H4K12 residues on specific regions of the genome. At this point, we wondered if the histone H4 acetylation levels in these eight regions reflected the effect observed for global histone H4 acetylation and behavior shown in Fig. 4b: an inverse correlation between acetylation and the distance to the average behavior of the population. We prepared four pooled samples (ten larvae per sample) with different behavioral distance to the average of the population (Fig. 5j), and we quantified the acH4 content within the eight selected regions (Fig. 5k). We found that the histone H4 acetylation levels in these regions decreased with the distance to the average behavior (*P* < 0.01 for all regions, two-sided *t*-tests), which suggested a role for these regions in the behavioral phenotypes previously observed. In addition, average histone H4 acetylation levels in these regions were not significantly altered during post-embryonic development (5–9 dpf, Additional file 1: Figure S6B, *P* > 0.05, two-sided *t*-tests), so we discarded any effect of subtle developmental differences across larvae.

### A complex composed by YY1–HDAC1 deacetylates histone H4 in target regions

**Fig. 6:**
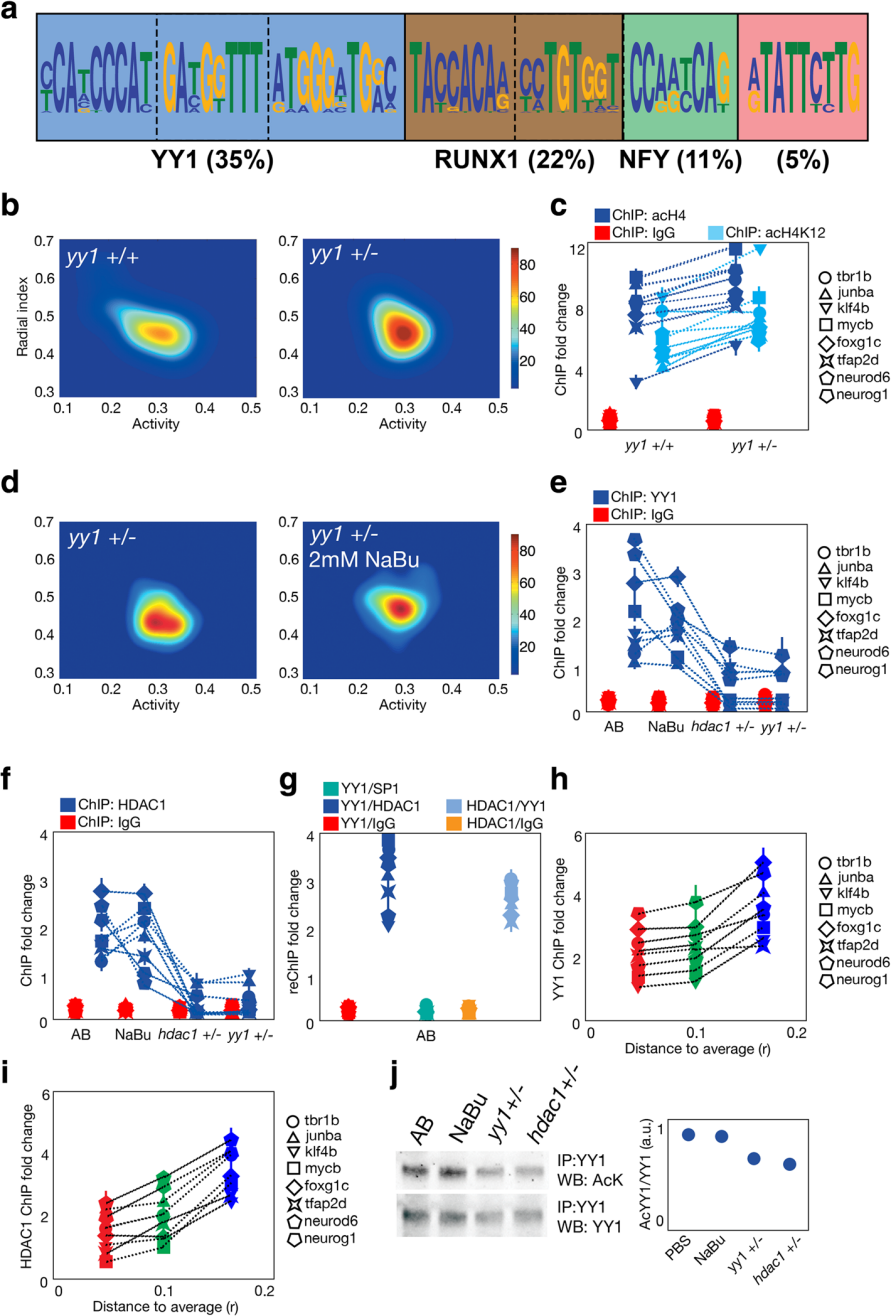
YY1 and HDAC1 role in inter-individual behavioral variability and acH4 changes. **a** The most represented motifs found in the acH4 peaks located near TSS. The predicted transcription factors that can bind to these sites are also indicated if found, with the percentage of the total peaks that present at least one of these motifs. **b** Probability density map for the behavior of 24 *yy1 +/+* (*left*) and *yy1 +/−* (*right*) fish. **c** AcH4 (*dark blue*), acetyl-H4K12 (*light blue*) and additional control IgG (*red*) levels found in *yy1 +/+* and *yy1 +/−* larvae, as detected by fold change compared to an unbound fraction in eight selected regions that are shown in the legend on the *right*. **d** The same as **b** but for *yy1 +/−* and *yy1 +/−* treated with 2 mM NaBu for 24 h. **e** YY1 binding (*blue dots*) and additional IgG presence (*red dots*) to the eight selected regions quantified by fold change compared to the unbound fraction in eight selected regions in AB (control), NaBu-treated, *hdac1 +/−*, and *yy1 +/−* larvae. **f** Same as **e** but for HDAC1 binding. **g** ReChIP fold change in control AB larvae. The order of the two consecutive ChIPs is noted in the names of the conditions. **h** YY1 binding in eight selected regions to clusters of larvae with different distances *r* to the average behavior of the population. **i** Same as **h** but for HDAC1 binding. **j** YY1 acetylation in control AB, NaBu-treated, *hdac1 +/−*, and *yy1 +/−* larvae. YY1-immunoprecipitated extracts were subjected to western blot analysis using an acetylated-lysine antibody (*top left*) or YY1 antibody (*bottom left*). Quantification of the acetyl-YY1/YY1 ratio is shown on the *right*

To find whether these regions could have a causal action in behavioral inter-individual variability, we decided to affect them by impairing DNA-interacting proteins that significantly bind near these regions. We found several DNA motifs that were enriched (*E*-value < 0.0001, MEME estimation [29]) near the candidate regions, Yin-Yang 1 (YY1; 35% of the total sequences and E-value < 10^−142^), RUNX1 (22%, E-value = 10^−112^), NFY (11%, E-value = 10^−53^) binding sites and even an unknown sequence (5%, E-value = 10^−42^) (Fig. 6a). YY1 is a transcription factor that can activate or repress the same target gene depending on recruited co-factors [30], HDAC1 [31] being one of its main partners. We then studied if YY1 might be implicated in the inter-individual variability by testing the behavior of a heterozygotic mutant *yy1a* (*yy1 +/−*) population (Fig. 6b). We found that this alteration decreased the behavioral inter-individual variability, measured by generalized variance (*P* = 0.003, permutation test) compared to wild-type counterparts. This result suggested that YY1 is necessary for the maintenance of this variability. As YY1 binding sites are present near the candidate regions, we quantified the differences in histone H4 acetylation that occurred near the eight previously selected regions in the *yy1+/−* population. We found that not only global histone H4 acetylation but also specific H4K12 acetylation were increased in the mutant background (Fig. 6c, *P*< 0.01 for all the regions except acetyl-H4K12 levels in *tbr1b*, *P* = 0.23, two-sided *t*-tests), similar to the results obtained in NaBu-treated and *hdac1 +/−* animals.

These results prompted us to study whether histone deacetylation and YY1 share the same pathway. First we tested the behavioral inter-individual variability of NaBu-treated *yy1 +/−* animals and a double heterozygotic *hdac1+/− yy1+/−* population (Fig. 6d). We found that NaBu treatment did not further decrease the behavioral inter-individual variability of the *yy1 +/−* fish (*P* = 0.54, permutation test), while the double heterozygotic mutation was lethal to the animals. Afterwards, we analyzed the recruitment of YY1 to the selected regions in wild-type, NaBu-treated, *yy1 +/−*, and *hdac1 +/−* populations by ChIP (Fig. 6e). YY1 binds to these regions in control conditions and the treatment with NaBu did not alter this recruitment (*P* > 0.05 for all the regions, two-sided *t*-tests). Interestingly, not only in the *yy1 +/−* but also in the *hdac1 +/−* populations, the binding of YY1 to the regions was decreased (*P* < 0.02 for all the regions, two-sided *t*-tests), suggesting that HDAC1 presence but not its activity was necessary for the recruitment of YY1 to the candidate regions. We also performed the same experiments for HDAC1 recruitment (Fig. 6f) and showed that the binding of the enzyme correlated to YY1 binding (*P* > 0.05 for AB vs NaBu, *P* < 0.01 for AB vs *yy1 +/−*, and AB vs *hdac1 +/−*, two-sided *t*-tests). Thus, these results not only point to a common pathway between YY1 and HDAC1, but also suggest they participate in the same regulatory complex in the candidate regions. Then we used double consecutive ChIP (reChIP) to confirm our hypothesis (Fig. 6g), showing that YY1 and HDAC1 were bound together to the eight regions (*P* < 0.01 for all the regions, two-sided *t*-tests). Finally, we performed the same experiment as shown in Fig. 5j, k, but looking at HDAC1 and YY1 recruitment to the target regions depending on the distance to the average behavior. We found that both HDAC1 and YY1 binding increases with the distance to the average behavior, consistent with the deacetylation process that occurs depending on this distance (Fig. 6h, i, *P* < 0.04 for all the regions, two-sided *t*-tests). As YY1 itself can be dynamically acetylated in a process in which HDAC1 participates [31], we analyzed YY1 acetylation by co-immunoprecipitation in different conditions (Fig. 6j and Additional file 1: Figure S6C). We found that YY1 is acetylated in basal conditions and treatment with NaBu does not seem to affect this acetylation (*P* = 0.51, two-sided *t*-test), while both in *hdac1 +/−* and *yy1 +/−* populations this acetylation is decreased (*P* < 0.01, two-sided *t*-test). As YY1 acetylation does not correlate with behavioral inter-individual variability or the chromatin changes previously observed, we cannot confirm a role for this modification in the phenotype. Nevertheless, we cannot exclude its participation in the dynamics of the recruitment or the activity of the YY1–HDAC1 complex.

### Gene expression is changed in the set of regions with alterations in histone H4 acetylation

**Fig. 7:**
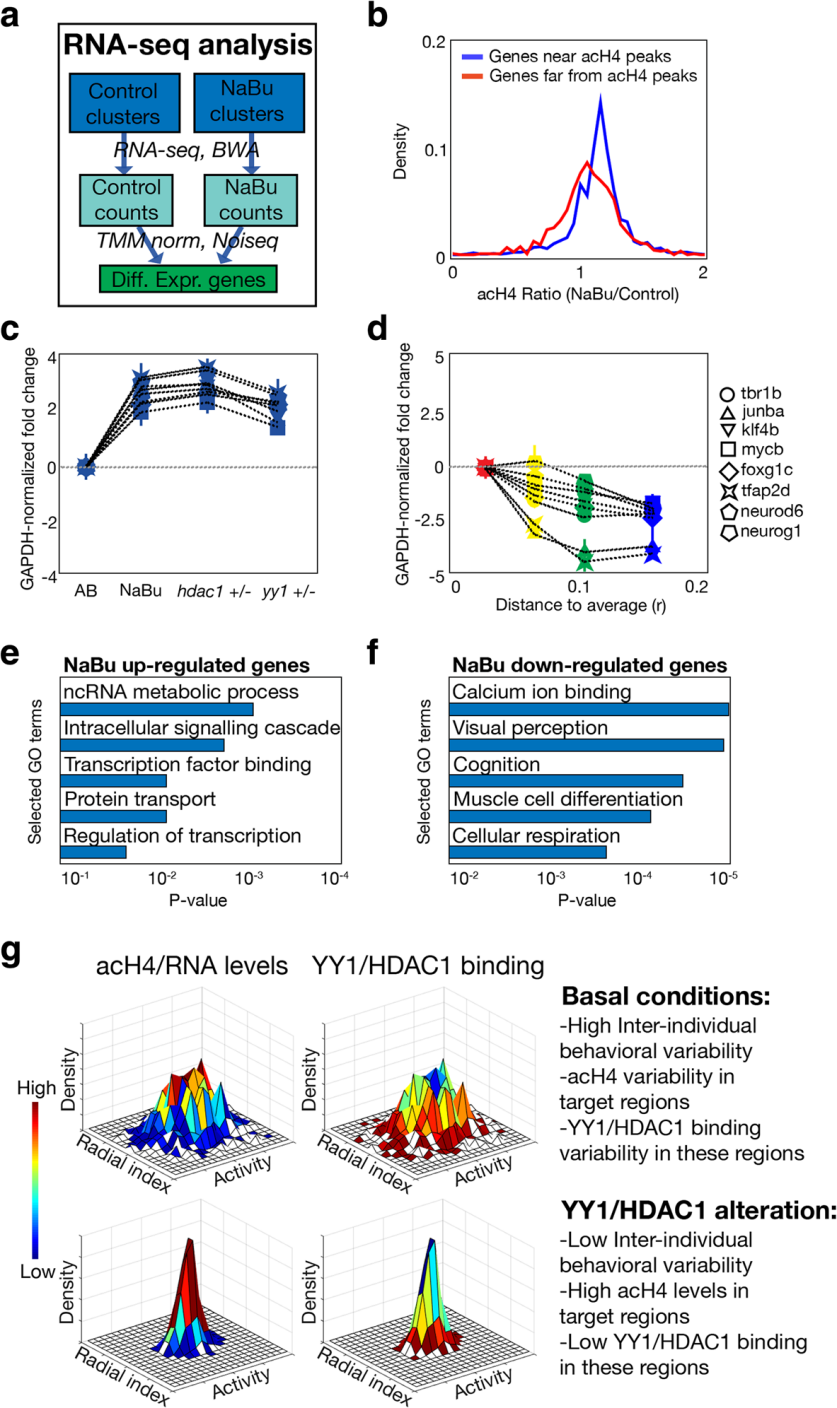
Conservation between epigenomic and transcriptomic results. **a** Workflow of the RNA-seq analysis of control and NaBu-treated samples. **b** The density of the gene expression ratio (NaBu normalized counts divided by control normalized counts) in the genes located near acH4 candidate peaks (*blue*) and genes not located near acH4 candidate peaks (*red*). **c** Gene expression fold change differences in eight selected genes in AB, NaBu-treated, *hdac1 +/−*, and *yy1 +/−* larvae. Normalization was made by subtracting the values obtained for the *gapdh* gene in each sample, and *bars* mark standard deviation obtained from three replicates. **d** Same as **c** but comparing samples with different distance *r* to the average behavior of the population. **e** Enriched Gene Ontology terms found in the set of genes over-expressed after NaBu treatment. **f** Same as **e** but for genes down-regulated after NaBu treatment. **g** Schematic model of the results obtained in the manuscript. In control conditions, heterogeneous populations as classified by their activity and radial index were obtained. After alteration of the YY1/HDAC1 pathway, more homogeneous populations were observed, while the acH4 content and the YY1/HDAC1 presence in a set of genomic regions were anti-correlated, these epigenetic changes being transferred to the gene expression level

So far we have found a relationship between a YY1–HDAC1 complex, histone H4 acetylation changes, and larval behavioral inter-individual variability. Still, a mechanistic explanation is needed to describe how these chromatin alterations could lead to altered behavior. One possible justification is that the transcriptional changes in the genes located near the candidate regions could lead to functional differences across individuals and finally to altered behavior. To test this, we used RNA-seq to analyze if the histone H4 acetylation changes observed in ChIP-seq were maintained at the gene expression level. We compared gene expression profiles retrieved for control zebrafish and NaBu-treated groups using the Noiseq algorithm [32] (which normalized gene counts using the Trimmed Mean of the M values (TMMs) [33]) and then applied a test to obtain differentially expressed genes by comparing the differences in the gene expression among groups of the same condition and groups of different conditions [32] (Fig. 7a, adjusted *P*-value < 0.1, Noiseq algorithm). There was a significant enrichment in the overlap between the over-expressed genes and the set of candidate peaks previously obtained in acH4 ChIP-seq (25%, *P* = 0.003, permutation tests). In addition, the genes located near the candidate peaks had higher expression after NaBu treatment compared to genes located far from these candidate peaks (Fig. 7b, *P* < 0.001, two-sided *t*-test).

We then verified the results obtained in the RNA-seq experiment by quantifying the expression of the genes located near the eight previously selected regions. As predicted by RNA-seq, and in parallel to histone H4 acetylation, gene expression was increased not only in NaBu-treated but also in *yy1 +/−* and *hdac1 +/−* animals (Fig. 7c, *P* < 0.01 for all the genes, two-sided *t*-tests). Moreover, gene expression decreased with the distance to the average behavior, similar to the effect that takes place at the histone H4 acetylation level (Fig. 7d, *P* < 0.01 for all the genes, two-sided *t*-tests).

In addition to the regions detected by acH4 peaks, other genes were also over-expressed (~ 3400) or repressed (~ 2100) after NaBu treatment using the Noiseq algorithm (see a list in Additional file 2: Tables S4 and S5). This effect confirms a major gene expression profile alteration after HDAC inhibition, being compatible with the enrichment obtained in the ChIP-seq analysis for transcription factors. In this way, the changes in histone H4 acetylation for several transcription factors would lead to an amplified gene expression response. GO terms enriched either in the up-regulated or the down-regulated genes were unique, showing that major cellular pathways are altered in NaBu-treated animals. Biological processes that are up-regulated include protein phosphorylation and signaling and RNA metabolism, in additional to transcriptional regulation, which was already predicted using the ChIP-seq data. Down-regulated processes include many metabolic pathways as well as cognition, response to light, and muscle cell development, among others (Fig. 7e, f and Additional file 2: Tables S6 and S7).

In summary, we have shown that behavioral inter-individual variability depends on a regulatory pathway that affects histone H4 acetylation. In control conditions, the YY1–HDAC1 complex deacetylates the histone H4 content of several regions located near genes coding for transcription factors. These changes, probably happening at the specific H4K12 mark, would lead to alterations in gene expression profiles that might result in individual differences in behavior. In the case of alteration of the YY1/HDAC1 pathway, these candidate regions are hyperacetylated and subsequently the neighboring genes overexpressed, leading to decreased inter-individual differences (Fig. 7g).

## Discussion

We have found that a histone H4 acetylation pathway modulates individual behavior in a genetics-independent manner without affecting the global average behavior of the population. Histone H4 acetylation levels of an individual correlated with its individual behavior compared to the average of the population. Therefore, while the average behavior might mostly depend on genetic background (as seen for different strains in Fig. 2) or environmental changes (as seen for different responses in Additional file 1: Figure S3F), behavioral inter-individual variability could result from histone H4 acetylation differences.

Several important questions arise from these results. The origin of genetics-independent changes in the individuals of an inbred population is still unknown. Our results suggest the existence of a stochastic basis for the generation of these individual differences. Several stochastic mechanisms could underlie behavioral inter-individual variability, such as paternal and maternal effects [34], differences in the experiences of individuals [4], or developmental noise, among others. Our results are consistent with the stochastic binding of a transcription factor to a set of target regions as the mechanism to generate transcriptional variability. Chromatin modifiers and histone marks have been shown to specifically affect gene expression noise [35, 36]. Thus, future studies will address the function of the YY1–HDAC1 complex in order to determine its binding dynamics. In addition, endogenous butyrate levels have been shown to be responsible for changes in behavior within a microbiota–gut–brain link [37]. ß-Hydroxybutyrate arising from lipid metabolism has also been found to endogenously inhibit HDACs, with the brain being one of the main target tissues [38]. It will be interesting to study inter-individual differences in terms of microbiota and metabolomic content, and their possible relation to behavior.

Another open question is related to how histone H4 acetylation changes could lead to behavioral inter-individual variability. We found that histone H4 acetylation levels are functionally transformed into changes in gene expression. In addition, genes located near the candidate regions are significantly related to transcriptional regulation, so differences in their expression might be amplified and this ultimately could lead to differences in processes like cognition or visual response, as our RNA-seq results suggested. Previous work has described how inter-individual variability in other behavioral paradigms underlies different physiological processes like neurogenesis [4, 11] and serotonin signaling [3, 12]. As some of the target genes of the YY1/HDAC1 pathway are related to neurogenesis, like *neurog1* [39], it will be interesting to study if gene expression differences for these transcription factors could lead to alterations in neurogenesis and/or the serotonin pathway. In addition, we have used activity and radial index as the parameters to quantify free-swimming behavior. As those might become proxy measures of anxiety levels and exploratory functions in zebrafish [40], it is even more relevant to characterize the serotonin pathway in the context of inter-individual variability in zebrafish.

Finally, we want to remark on an interesting question that also arises from our data. A group of fish with high levels of histone H4 acetylation will behave more similarly to one another than a different group of fish with lower levels of histone H4 acetylation. A potential explanation might be that the histone H4 acetylation (and consequently the gene expression) profiles become more different as their total levels decrease, due to stochastic binding to target regions. Nevertheless, we cannot exclude the changes that occur at the cellular and/or tissue levels, or that gene interactions participate in the generation of inter-individual differences. A future combination of experiments and theoretical modeling might clarify the generation of inter-individual variability by differences in histone H4 acetylation levels.

## Conclusions

Our data suggest that Histone acetylation differences across individuals are important for the development of behavioral inter-individual variability in zebrafish. A transcription factor, YY1, seems to provide target specificity to this pathway.

## Methods

### Zebrafish lines and care

Zebrafish (*Danio rerio*) WIK strain [41] was kindly provided by Dr. Bovolenta (CBM-UAM) and inbred in our laboratory for at least three generations before the experiments. Afterwards, a WIK F1 population was generated from a single batch of embryos from a single couple of adult fish. Two additional cycles of inbreeding were carried out, crossing a couple of siblings from the former generation. The CG2 clone population [17], generated by double gymnogenetic heat-shock and characterized by being pure isogenic zebrafish, was kindly provided by Dr. Revskoy (Univ Northwestern) as a control for reduced genetic differences between siblings. The outbred LPS (Local Pet Store) strain was recently described [42] and was used as a model of genetic heterogeneity. Heterozygotic *hdac1* (*hi1618*) and *yy1a* (*sa7439*) mutant strains with wild-type (AB strain) counterparts were obtained from ZIRC, while heterozygotic *hdac1* (*sa436*) [21] with wild-type counterparts were kindly provided by Dr. Ober (University of Copenhagen).

Care and breeding of the zebrafish strains were as described [42], with specific additional details. Eggs were isolated 24 h post-fertilization and maintained in custom multiwell plates until 10 days post-fertilization (dpf). They were fed (JBL NovoBaby) from 6 dpf and water was changed daily if not indicated specifically in the experiment.

### Free-swimming setup and recording

The setup consists of a monochrome camera located over the wells at a distance of 70 cm and pointing downwards. The camera used was a 1.2 MPixel camera (Basler A622f, with a Pentax objective of focal length 16 mm). The wells are circular, carved on transparent PMMA (24 wells per plate, and typically two plates are recorded simultaneously), and have their walls tilted so that even in the most lateral wells the wall never hides the larva from the camera. Each well is 15 mm deep, and has a diameter of 1.8 mm at the bottom and a diameter of 30 mm at the top (Additional file 1: Figure S1A). For the experiments, each well is filled with a volume of 3 ml. The dishes are supported by a white PMMA surface that is only partially opaque. Behind this white surface we place two infrared LED arrays (830 nm, TSHG8400 Vishay Semiconductors) pointing outwards (Additional file 1: Figure S1A). Two paper sheets stand between the lights and the central space that lies directly under the wells. With this disposition we ensure that only diffuse indirect light reaches the wells, so that the illumination is roughly uniform (most of the light comes from below the wells through the white surface). White curtains entirely surround the set-up. The video camera recorded at 25 fps (Additional file 1: Figure S1B, C for examples of a single frame and final trajectories).

A larval population (5–8 dpf) consisted of at least 24 fish siblings from the same batch of embryos. After 5 min of acclimation to the new environment, the larvae were recorded for 20 min. Water temperature was maintained in a strict range (27–28 °C) during each experiment.

### Custom-built software tracking larvae

We developed multiwellTracker, a software to automatically track zebrafish larva in wells. The software is available at http://www.multiwelltracker.es.

#### Detection of wells

The program is prepared to auto-detect circular wells, regardless of their spatial arrangement. To detect the wells we use the circular Hough transform (we have modified the code of Tao Peng distributed by Matlab Central under BSD license). In order to estimate the diameter of the wells, it computes the image’s Hough transform for 100 radii different in 5 pixels and a rough estimate of the largest possible radius (length of the longer side of the image divided by the square root of the number of wells) (Additional file 1: Figure S1D). The system selects the highest point of this measure as an estimate of the radius of the wells (r_est_). It is possible to skip this first step and instead manually specify a value for r_est_. This may be advisable when many videos are recorded with the same set-up and the same wells.

In the second step the system locates the centers of the wells. To do this it performs a Hough transform of the original image, this time with radii only in the range between 0.8r_est_ and 1.2r_est_. The transformed image usually has clear peaks in the centers of the wells. Then it filters the transformed image with a Gaussian filter to increase its smoothness (the resulting transformed image is shown in Additional file 1: Figure S1E). Then, it selects the maximum of the transformed image as the center of the first well. To prevent selecting the same well twice, the system discards all the pixels of the transformed image that are within radius r_est_ of the selected center (Additional file 1: Figure S1F). It selects the new maximum as the center of the second well, and repeats the procedure until all wells have been found (Additional file 1: Figure S1G). The experimenter can correct the result by manually clicking on the center of the wells that have not been correctly located (< 1% of cases).

#### Pre-processing of images

In order to control for fluctuations in illumination, each frame is normalized by dividing the intensity of each pixel by the average intensity across all pixels of the frame. After normalizing the frame, a 2D Gaussian filter is used to smooth the image (Additional file 1: Figure S1H shows the image before and Additional file 1: Figure S1I after filtering).

#### Background subtraction and detection of the larva

In order to extract the image of the larva from the background, the system finds the average of 1000 frames equi-spaced along the whole video. This average image is what we will call “static background”. By substracting the static background from each frame, we obtain an image in which the larvae correspond to dark regions (Additional file 1: Figure S1J). However, because of relatively slow changes in the set-up over time, the system uses the static background in combination with a dynamic background, which is computed as the average of the previous five frames. The difference between the current frame and the dynamic background will only show larvae that are moving in that precise moment (Additional file 1: Figure S1K).

The specific algorithm to detect the larva is as follows. First, the difference between the current frame and the static background is thresholded, keeping only pixels for which the difference is below − 0.5. We then find connected components (“blobs”) in this thresholded image, keeping those that are larger than one pixel. Because these blobs come from the difference with the static background, both static and moving larvae will be detected. But at this stage some blobs come simply from noise. In order to filter out noisy blobs, the system accepts a blob if it fulfills at least one of these two conditions: (a) it contains at least one pixel that was identified as part of the larva in the previous frame or (b) it contains at least one pixel for which the difference between the current frame minus the dynamic background is below the same threshold as before (− 0.5).

#### Removal of reflections

In most cases only one blob is obtained after the process described in the previous sections. But when the larva is close to the wall of the well, its reflection on the wall may also be selected. The system considers that a blob A is a reflection of blob B when all of the following conditions are met: (a) blob B is bigger than blob A, (b) blob B is closer to the center of the well than blob A, and (c) the lines between the center of the well and the two blobs form an angle < 10°. When these three conditions are met, the system removes blob A.

#### Acquisition of the position of the larvae

If more than one blob remains in the same well after the previous steps, the system selects the one with highest contrast. For the selected blob, the system takes the position of its most contrasted pixel, and adds this position to the trajectory of the larva. If in a well no blob remains after the previous steps, the trajectory is left with a gap. When this happens, the program will not re-track the larva until it moves.

### Behavioral parameters

Different parameters reflecting the behavior of individual larvae were measured, and two were used throughout the paper: (i) activity (percentage of time in movement) and (ii) radial index (average position from the border towards the center of the well). We also studied six additional parameters: (iii) tortuosity in the trajectory was calculated as the scalar product of the velocity vectors between two consecutive frames and the value in Additional file 1: Figure S2C was obtained by averaging this parameter through the whole video, excluding the frames where the animal was immobile. (iv) Speed was calculated as the average distance (in pixels) travelled per frame, in those frames where the fish was active. (v) Bursting was obtained as the total number of frames where fish changed from immobility to motion. (vi) Circularity was calculated as the distance travelled in the border/center axis divided by the total distance travelled by the individual. (vii) Instant acceleration was obtained as the average distance travelled by the individual in the frames where the fish changed from immobility to motion. (viii) Instant tortuosity was the average tortuosity in the frames where the fish changed from immobility to motion.

The average of each individual parameter was tested from 5 to 8 dpf to assess if individual behavior was significantly stable along the days using Pearson coefficient of correlation.

### Inter-individual vs intra-individual differences

The eight behavioral parameters were also obtained from consecutive fragments of 30 s for each 20-min experiment for each larva. This was fitted to a two-dimensional Gaussian, but for clarity when representing many animals an isocontour of the Gaussian for each animal was used. An isocontour is an ellipse with principal axes given by the eigenvectors of the covariance matrix. We chose the isocontour with length of each semiaxis given by the square root of the eigenvalue of the covariance matrix, as this reduces to the standard deviation in each direction for cases with no correlation between the two variables. Intra-individual variation distribution was obtained using the coefficients of variation (CVs) and the standard deviations (only in the case of tortuosity and instant tortuosity, due to the presence of positive and negative values) separately. Inter-individual variation was calculated the same way but using fragments from different fish.

### Additional validation of the experimental setup

Several controls were performed for possible experimental artifacts affecting wells differently. Behavioral parameters (activity and radial index) were robust to 90 degrees counterclockwise rotations of the multi-well plate (Additional file 1: Figure S3A, left; *R* = 0.73, *P* < 0.001, and *R* = 0.68, *P* < 0.001; two-sided *t*-tests for R = 0) or to interchanging the larvae between outer and inner wells (Additional file 1: Figure S3A, right; *R* = 0.65, *P* < 0.001, and *R* = 0.61, *P* < 0.001; using the same test as before). Also, we found no correlation between the small differences in illumination across wells and behavior (Additional file 1: Figure S3B). We further corroborated using a significance test that the differences in behavior did not have an origin in systematic differences across wells. For this, we found that the average behavioral parameters obtained in 15 individual experiments were not different between wells (Additional file 1: Figure S3C, typically *P* = 0.4 and always *P* > 0.19 for both parameters, permutation tests).

### Stimulus response tests

We studied the influence that our free-swimming behavioral parameters could have on the performance of the individuals when they respond to three different stimuli.

#### Response to mechanical disturbance

We applied mechanical perturbations to each larva by pipetting the water content of the well up and down four times. Perturbations were applied at 6 dpf to previously recorded animals, and the 20-min recording was done at 7 dpf. The recording was performed in the usual setup.

#### Response to strong light pulse

In complete darkness, we applied three different light flashes to the larvae and studied their behavior in the 90 subsequent seconds. The flashes and the recording were performed in the usual setup. Pre-recording behavioral parameters were obtained the day before.

#### Novel tank with light/dark preference

In order to study the effect that a novel setup could have on the behavior of larvae we built a rectangular setup, which changed the geometry of the previous circular wells. The setup dimensions were 84 mm × 21 mm and it was built in transparent acrylic. To try to see if our parameters had any effect on the light–dark preference, half of the floor of the setup (42 mm × 21 mm) was white while the other half was black. The height of the setup was 5 mm. Larvae were placed in the center of the white part and recorded for 10 min. Activity was calculated as previously described and distance to the wall was represented by the average distance to the longest walls, normalized to 1 in the middle point of both walls and to 0 at the exact position of the walls.

The effect of our behavioral tests resulted in a decrease (increase) in mean activity (radial index), but maintaining the same individuality of the pre-recorded free-swimming experiments (Additional file 1: Figure S3D; *P* < 0.04 for changes in mean activity and radial index compared to control larvae of the same age, two-sided *t*-tests; *P* < 0.02 in two-sided *t*-tests for R = 0 between activity and radial index). In the case of the novel tank, radial index cannot be applied because the wells are elongated so was replaced by the minimum distance to the longer walls. We note, however, that this parameter showed no correlation with the radial index of pre-recorded experiments in the same animals.

### Comparing the behavioral variability between two animal groups

A simple visual method to characterize the variability in a population is to plot the bi-dimensional distribution of the activity and radial index of individuals (like in Fig. 1a’”). To do so, we used Gaussian kernel smoothing that consists in adding up Gaussians centered at the data points as:

$$ P\left(x,y\right)=\frac{1}{N}\frac{1}{2{\pi \sigma}_x{\sigma}_y}\sum \limits_{i=1}^N\exp \left(-\frac{1}{2}\left[\frac{{\left(x-{x}_i\right)}^2}{\sigma_x^2}+\frac{{\left(y-{y}_i\right)}^2}{\sigma_y^2}\right]\right) $$

with *x*_i_ and *y*_i_ the mean activity and radial index values of individual *i* of a total of *N* members of the population. An optimal smoothing uses standard deviations of each Gaussian given by *σ*_*x*_ = *N*^−1/6^*α*_*x*_ with *α*_*x*_ the standard deviation in the *x*_i_ data values, and similarly for *σ*_*y*_ using the *y*_i_ values (see B.E. Hansen, unpublished manuscript, http://www.ssc.wisc.edu/~bhansen/718/NonParametrics1.pdf). The volume under the probability surface has a value of 1, even when the values of the probability density are already up to 90. The probability surface sits on an area on the x–y plane of approximately 0.4 × 0.4, making the total volume under the surface 1.

While this distribution gives a visual and intuitive characterization of behavioral variability in two dimensions, for statistical tests we found it advantageous to reduce it to a single parameter measuring dispersion on the plane. The variance ${\sigma}_x^2\left({\sigma}_y^2\right)$ measures dispersion in *x*(*y*). In more dimensions, a measure of dispersion that takes into account the covariance structure is the hypervolume that the distribution occupies in space. This is measured by the generalized variance, which can be computed as the determinant of the covariance matrix, |Σ| [16]. To gain intuition, note that generalized variance reduces in two dimensions to ${\sigma}_x^2{\sigma}_y^2\left(1-{\rho}^2\right)$, with *ρ* the Pearson correlation. When the two variables have no correlation *ρ* = 0 and generalized variance is maximal. Correlation makes *ρ* closer to 1 and the two variables closer to a line, making dispersion on the plane and generalized variance smaller. For statistical tests, we use the generalized variance of the behavior (activity/radial index) of populations composed by at least 24 individuals (Additional file 2: Table S1), and additionally the standard deviation for each parameter (Additional file 2: Table S2).

### ChIP-seq, conventional ChIP, and reChIP analyses

Chromatin immunoprecipitation was obtained from pooled samples of at least four zebrafish larvae. Briefly, the samples were crosslinked with 1.8% formaldehyde for 30′ and then quenched with 1% glycine for 5′. Extracts were lysed using a SDS lysis buffer (50 mM Tris-HCl pH 8.1, 1% SDS, 10 mM EDTA) for 30′ at 4 °C, and then diluted with a dilution buffer (6.7 mM Tris-HCl pH 8.1, 0.01% SDS, 1.2 mM EDTA, 1.1% Triton X-100, 167 mM NaCl). Sodium butyrate (2 mM) was added to avoid histone deacetylation activity during the preparation. Then, the fish were sonicated with two pulses (30′′ ON/30′′ OFF) of 15′ each with the Diagenode Bioruptor. Before pre-clearing the samples with protein A/G beads, an input sample was obtained. Then, the extracts were immunoprecipitated overnight using 1 μg of anti-acetyl-Histone 4, anti-HDAC1, or anti-YY1 antibodies. Bound DNA was recovered with protein A/G beads, then washed with low-salt (120 mM Tris-HCl pH 8, 0.1% SDS, 2 mM EDTA, 1% Triton X-100, 150 mM NaCl), high-salt (120 mM Tris-HCl pH 8, 0.1% SDS, 2 mM EDTA, 1% Triton X-100, 500 mM NaCl), LiCl (10 mM Tris-HCl pH 10, 1 mM EDTA, 0.25 M LiCl, 1% NP40, 1% sodium deoxycholate) and two times with 1× TE (10 mM Tris-HCl pH 8, 1 mM EDTA) buffers, and recovered with elution buffer (1% SDS, 0.1 M NaHCO3). DNA purified samples were de-crosslinked using sodium chloride and cleared with Qiagen spin columns, and for reChIP, the samples were re-incubated with a second antibody after different elution [43], and another round of washes and elution was performed.

In the case of conventional ChIP, qPCR was used to detect differences between the samples in the target regions, using the unbound fraction (ChIP from the same sample but without antibody) as a control to normalize results.

In the case of the acH4 ChIP-seq, we prepared 12 samples (four control, four NaBu, and four random) composed of five larvae each, chosen by the following algorithm. For the control samples, we needed to obtain clusters of larvae with very similar behavior, so we performed hierarchical clustering (using Euclidean distance as the metric and average linkage criteria) of the behavior (activity and radial index) of a population of 72 larvae. The selection in the NaBu samples used the same algorithm, but using 48 larvae. Previous experiments indicated that the behavioral variability between the NaBu samples would be reduced compared to control samples (Fig. 3b). In the random experiment, fish were randomly selected from the population, obtaining samples composed of five random larvae without any behavioral consideration. We postulated that the variability across these random samples was not associated with behavior. In summary, we obtained four samples with high behavioral variability (control samples), four samples with low behavioral variability (NaBu samples), and four samples with low variability that is not associated with behavioral differences (random samples). Even when the use of pooled fish might introduce variation into the results, our previous results showing that acH4 levels are very similar between larvae with the same behavior (Additional file 1: Figure S5C) suggested that this effect would be minor.

The final samples were processed at the Genomics Unit at the Scientific Park of Madrid. Libraries were built and the samples were sequenced using an Illumina GAIIX. Reads were aligned to *Danio rerio* genome sequence (Zv9) with BWA, and the results were subjected to the MACS peak detection algorithm [44]. Afterwards, peaks from the different samples were merged and quantified separately as fragments per kilobase per million reads (FPKM) using the DiffBind package [45], obtaining 27,310 peaks in control samples and 33,649 peaks in NaBu samples, with 12,419 peaks shared by both conditions. Finally, EdgeR [46] was applied to detect differential binding between control and NaBu samples. The candidate peaks with higher histone H4 acetylation in NaBu compared to control (*P* < 0.001) were further filtered using the random samples. Specifically, we removed the regions with higher variability (measured with the coefficient of variation) in random samples, as this variability is not associated with behavior.

### Gene Ontology and transcription factor binding site analysis

Position of the candidate peaks obtained in the ChIP-seq was retrieved in order to study their position relative to near genes. Nearest genes were retrievedand analyzed for Gene Ontology using DAVID [47] as in the case of RNA-seq targets. In addition, the candidate regions located near the TSS of a gene were used to predict enriched DNA motifs and their potential biological activity with the MEME suite [29].

### Reagents and antibodies

Sodium butyrate (NaBu), trichostatin A (TSA), cambinol (Cmb), and 5-aza-2′-deoxycytidine (AZA) were purchased from Sigma-Aldrich (#303410, #T8552, #C0494, and #A3656) and dissolved in phosphate-buffered saline (PBS) to final concentrations of 2 mM, 0.1 μM, 0.2 μM, and 15 mM in the fishes’ water, respectively. PBS alone was used as vehicle control. The pharmacological treatment lasted for 24 h from 7 to 8dpf. Acetyl-histone 4 and acetyl-lysine antibodies were obtained from Millipore (#06-866 and #05-515), anti-HDAC1 and anti-YY1 from ActiveMotif (#39531 and #61780), anti-Sp1 and anti-H4 from Abcam (ab59257 and ab16483), and McrBC enzyme from New England Biolabs (M0272).

### Immunoprecipitation and western immunoblotting

Groups of 20 fish (control, NaBu-treated, *hdac1 +/−*, and *yy1 +/−*) were frozen at 8 dpf. They were lysed in a solution containing 100 mM Tris-HCl pH 7.5, 20 mM NaF, 2 mM DTT, 2 mM EGTA, 2 mM EDTA, 1 mM sodium orthovanadate, 0.54 M sucrose, 0.2 mM phenyl-methyl sulfonyl fluoride, 2% X-100 Triton, ß-mercaptoethanol, and 4 μg/ml complete protease inhibitor cocktail (Roche, #11836153001). For each condition/treatment an aliquot of 1 mg protein was incubated with 2 μg of anti-YY1 antibody and 30 μl of protein-A/G plus Sepharose beads overnight at 4 °C. Beads were then washed five times with washing buffer (20 mM Tris-HCl pH 7.4, 50 mM NaCl, and 4 μg/ml complete protease inhibitor cocktail). Immunoprecipitated proteins were analyzed by western blotting using anti-acetylated lysine antibody and YY1 antibody.

### RNA isolation, qPCR quantification, and RNA-seq

Total RNA was isolated using homogenized extracts from at least five fish per sample by RNeasy Mini purification (Qiagen, #74104). Retrotranscription was done with iScript (Bio-Rad, #1708891) following the manufacturer’s recommendations. Finally, quantification of the target genes was measured using qPCR with specific primers. Values were normalized using the GAPDH results obtained from the same sample, and *P* values obtained by using Student’s *t*-test. In the case of RNA-seq, three control and three NaBu samples were obtained using the same algorithm described for the ChIP-seq experiments. RNA samples were processed at the Genomics Unit at the Scientific Park of Madrid to generate libraries, and raw reads were obtained using Illumina GAII. Afterwards, the reads were aligned to the *Danio rerio* genome (Zv9) using BWA, and transcript counts were normalized to TMM (trimmed mean of M-values) scores in order to be able to compare the gene expression across samples. Then, the NoiseqBIO algorithm from Noiseq [32] was used to detect significantly (adjusted *P* value < 0.1) differentially expressed genes in the two groups with three biological replicates each.

### Quantification of histone acetylation levels

Eighteen clusters of five fish from a total population of 90 were obtained from the behavioral space (activity/radial index) using an ad hoc algorithm. First, 18 centroids were randomly chosen, and five individuals were assigned to the nearest (not occupied) centroid. Then, centroids were redefined using the average values of the new clusters, and a new round of assignment of the fish to the centroids was done. This iteration was repeated until the centroids were stable. Then, total histones were extracted using an Epigentek kit (OP-0006) and quantified by Pierce Coomasie Plus reagent (Thermofisher, #27236) in order to use the same amount of total histones in each sample. Acetyl-H4, acetyl-H3 and specific acH4 marks (H4K5, H4K8, H4K12, and H4K16) were quantified by ELISA following the manufacturer’s recommendations using the Epigentek kits P-4009, P-4008, and P-3102, respectively.

### Quantification of methylated DNA

DNA methylation was quantified using larval DNA digested by MCrBC enzyme as previously done [48] following the kit instructions.

### Statistical analysis

In the case of linear correlation between behavioral parameters or across days, the statistical tests assessed the null hypothesis that the correlation coefficient is equal to 0, using two-sided *t*-tests. In the case of correlation between bulk histone acetylation and behavioral parameters (distance to the average or angle to the average), a similar approach was performed.

When the differences between the inter-individual variability and the intra-individual variability of a behavioral parameter were assessed, the set of 30-s fragments of the trajectories of all the individuals were shuffled. Then, this random inter-individual and intra-individual variability was calculated (using the CV) and we compared these permutated values to the observed in the real dataset. Finally, by doing this process 1000 times, we were able to obtain a *P* value (the proportion of times in which the final values were higher in the case of permutation datasets) for testing the hypothesis that the inter-individual variability is equal to the intra-individual variability.

In the case of the analysis of changes in behavioral inter-individual variability between two groups, we compared the difference between the generalized variance of these groups and the difference of two groups obtained after permuting the datasets. Specifically, we shuffled the individuals between the two groups, generating random groups of individuals. Then, we obtained the difference between the generalized variance in these two random groups and compared it to the real value. By doing this 1000 times, we obtained a *P* value for testing the hypothesis that the generalized variance of the two groups is the same.

To test acetylated histone or methylated DNA differences between two groups, the levels of these groups were compared using two-sided *t*-tests. In the case of ChIP, reChIP, and gene expression analyses, the statistical tests to compare the difference between two conditions were conducted by calculating the representative parameter (fold change compared to a control, gene expression ratio between two groups), and *P* values were obtained using two-sided *t*-tests.

For motif finding, the MEME algorithm [29] was used to estimate an E-value of each motif, a parameter that quantifies the log-likelihood ratio of the motif depending on its size and the letter composition of the background.

In the case of ChIP-seq analysis, a *P* value was extracted using the EdgeR algorithm, which calculates an exact test using the quantile-adjusted conditional maximum likelihood following a negative binomial model on the normalized counts of the samples.

All the experiments (except ChIP-seq and RNA-seq) were done at least three times with different biological datasets, and *P* values were calculated using the three replicates. Figures related to behavior show a representative experiment of the triplicate, while molecular analyses show the average result with error bars representing the standard deviation. MATLAB and R were used for all the computations and the statistical analysis.

## Additional files

Additional file 1: **Figures S1–S6**. (PDF 18657 kb)

Additional file 2: **Tables S1–S7**. (DOCX 208 kb)
