# Supplementary material for: Histone H4 acetylation regulates behavioral inter-individual variability in zebrafish
Source: Genome Biol. 2018 Apr 25;19:55. doi: 10.1186/s13059-018-1428-y (PMC5922312; doi:10.1186/s13059-018-1428-y)
Supplement: Supplementary file 1 — Figures S1–S6. (PDF 18657 kb) [file 13059_2018_1428_MOESM1_ESM.pdf]

Figure S1. **Setup and tracking system. (A)** Scheme of the setup with an enlarged scheme of one of the wells. **(B)** Image of the wells. **(C)** Trajectory of each larva. **(D)** Strength of the peaks resulting from Hough transforms with different radii. The insets show the Hough transforms for 3 different radii. From this calculation we define rest = 58 pixels. **(E)** Hough transform of the frame, using radii in a small interval around rest (in this case, in the interval between 47 and 71 pixels). **(F)** Same as **E**, but after removing the region that corresponds to the highest maximum. **(G)** Final result of the detection of wells. **(H)** Original image of two adjacent wells. **(I)** Image of the same wells, after a Gaussian filter. **(J)** Difference between the filtered image of the two wells, and the static background. The two larvae are clearly visible as regions with low values. **(K)** Difference between the filtered image of the two wells, and the dynamic background. Only the larva on the right is visible, because the larva on the left is not moving in this specific frame.

Figure S2. Additional behavioral parameters. **(A-F)** Correlation of six behavioral parameters to activity or radial index (in the case of circularity). **(G-J)** Correlation of the eight behavioral parameters between 5 dpf and 6 dpf. **(K-M)** The same as **G-J** but using six behavioral parameters between 7 dpf and 8 dpf. **(N-P)** Median of intra-individual variability (dotted lines) and inter-individual variability using six parameters during 5-8 dpf. **(Q)** Smoothed histogram of the coefficient of variation of the activity (left) and the radial index (right) showing the intra-individual variability (red) and inter-individual variability (blue) at 5 dpf. **(R)** Same as **Q**, for the group at 6 dpf. **(S)** Same as **Q**, for the group at 7 dpf. **(T)** Same as **Q**, for the group at 8 dpf.

Figure S3. **Additional validation of activity and radial index. (A)** Changes in the behavior after 90° rotation of the plates (left) and after interchanging 24 larvae from external to internal wells (right). **(B)** Larval behavioral parameters (activity, blue; radial index, red) plotted against

the average light received by these 48 larvae. Min and max values are 0.1-0.5 and 0.3-0.7 for activity and radial index, respectively. **(C)** Average activity (left) and radial index (right) of a pool of different larvae recorded in the same wells for 15 experiments (>600 larvae) (top), compared to a single 48 larval population (bottom). **(D)** Activity (left) and radial index (right) plotted against the median size of the larva as detected by the tracking software. **(E)** Average activity (left) and radial index (right) of groups of ten larvae plotted against the gene expression fold change of *amigo1* normalized by *GAPDH* expression **(F)** Correlation between the behavior in different tests (Light Pulse, left; Mechanical Perturbation, center and Novel Tank with Light Preference, right) and our behavioral parameters in the usual conditions, using 16 larvae.

Figure S4. **Additional data for epigenetic treatments. (A)** Methylation levels comparing control (PBS) and AZA treatment. **(B)** ELISA results quantifying the levels of acetyl-H4 in the conditions shown in **Figure 3** and in groups of 20 fish from 5 to 9 dpf. **(C)** Probability density for 24 larvae 48h after treatment with NaBu only during the first 24h and then washed with PBS (PBS/PBS as control).

Figure S5. **Additional data for histone H4 acetylation and behavior. (A)** Relation between histone H4 acetylation levels and distance to average behavior: activity (left) and radial index (right). R coefficients are shown. (**B**) Same as **A**, but for H4K12 acetylation levels. (**C**) Differences in histone H3 (red) and H4 (blue) acetylation levels within extracts obtained in the same behavioral positions towards the average behavior of the population (left). Difference in histone H4 acetylation in clusters with different behavioral positions towards the average behavior of the population (right).

Figure S6. **Additional data for Histone H4 and YY1 acetylation.** **(A)** Plot showing acH4 normalized levels for each region in control samples (x-axis) versus random samples (y-axis), whose variability is not associated to behavioral differences **(B)** Histone H4 acetylation levels quantified in conventional ChIP as the fold change compared to non-bound fraction in eight selected regions in pools of 20 fish from 5 to 9 dpf. **(C)** Original blot from the **figure 6J** representing the acetyl-YY1 levels at different conditions.

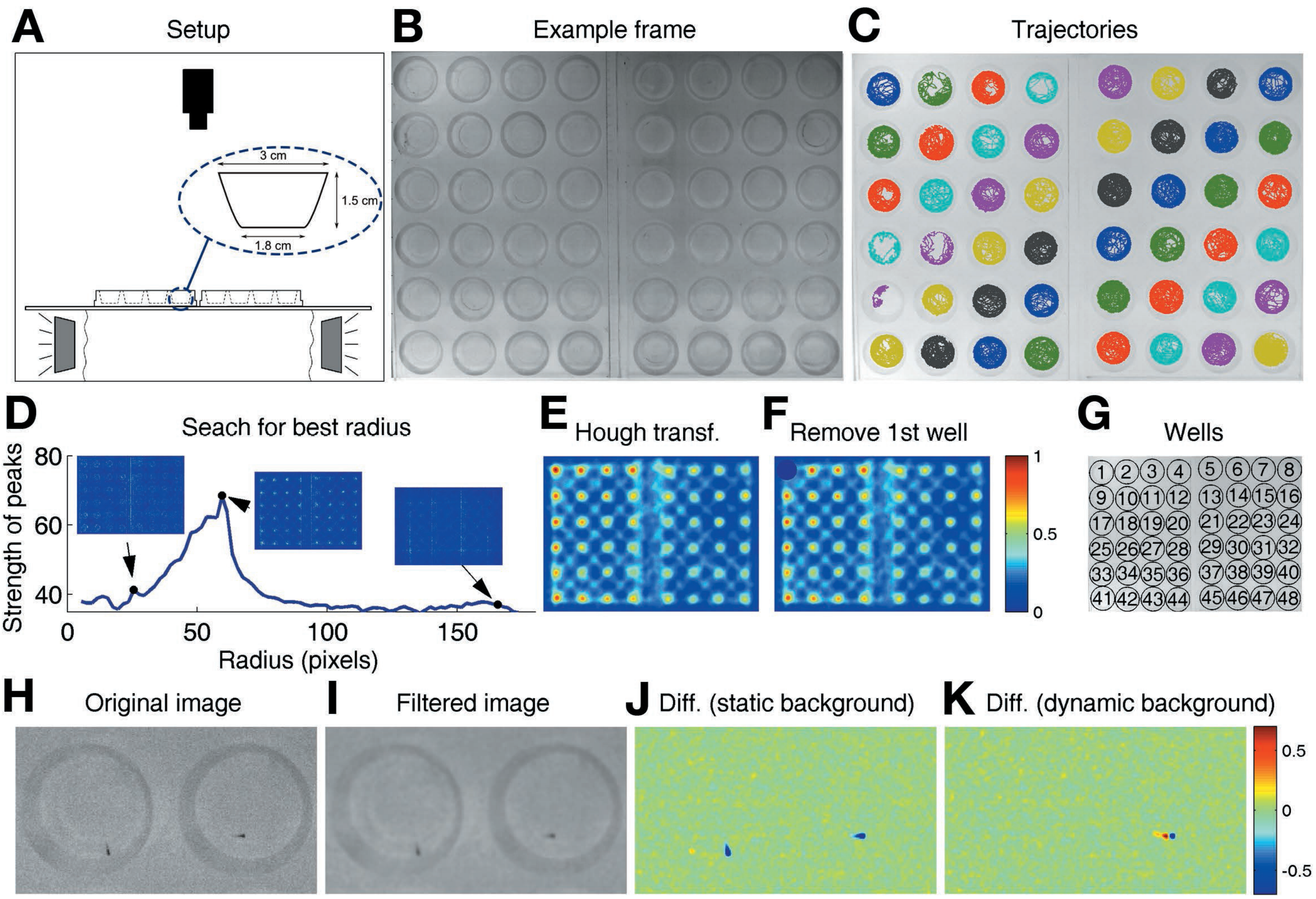
A
Setup
3 cm
1.5 cm
1.8 cm
B
Example frame
C
Trajectories
D
Seach for best radius
Strength of peaks
80
60
40
0
50
100
150
Radius (pixels)
E
Hough transf.
F
Remove 1st well
1
0.5
0
G
Wells
1 2 3 4 5 6 7 8
9 10 11 12 13 14 15 16
17 18 19 20 21 22 23 24
25 26 27 28 29 30 31 32
33 34 35 36 37 38 39 40
41 42 43 44 45 46 47 48
H
Original image
I
Filtered image
J
Diff. (static background)
K
Diff. (dynamic background)
0.5
0
-0.5

Figure S1

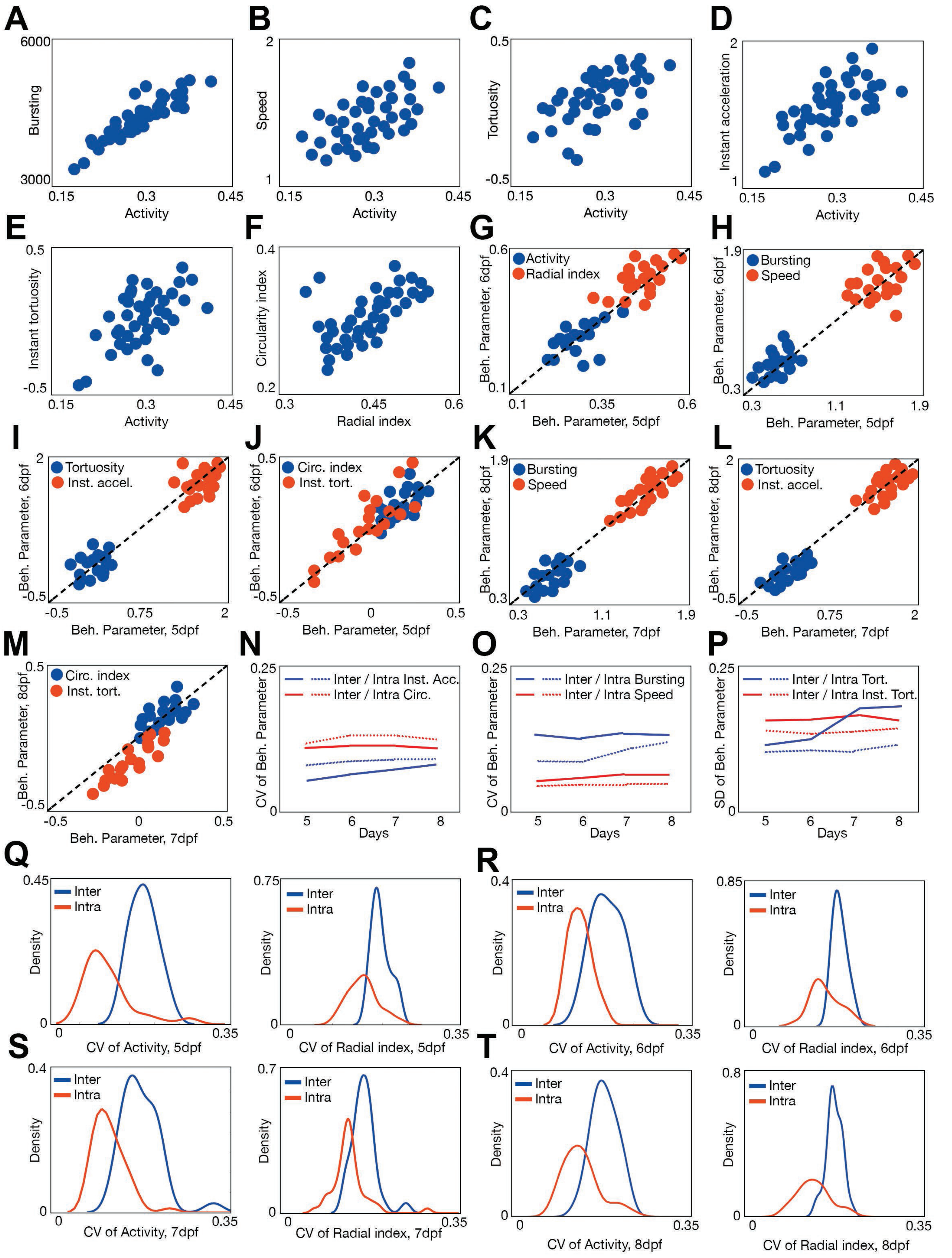

A
Bursting
Activity
B
Speed
Activity
C
Tortuosity
Activity
D
Instant acceleration
Activity
E
Instant tortuosity
Activity
F
Circularity index
Radial index
G
Activity
Radial index
Beh. Parameter, 6dpf
Beh. Parameter, 5dpf
H
Bursting
Speed
Beh. Parameter, 6dpf
Beh. Parameter, 5dpf
I
Tortuosity
Inst. accel.
Beh. Parameter, 6dpf
Beh. Parameter, 5dpf
J
Circ. index
Inst. tort.
Beh. Parameter, 6dpf
Beh. Parameter, 5dpf
K
Bursting
Speed
Beh. Parameter, 8dpf
Beh. Parameter, 7dpf
L
Tortuosity
Inst. accel.
Beh. Parameter, 8dpf
Beh. Parameter, 7dpf
M
Circ. index
Inst. tort.
Beh. Parameter, 8dpf
Beh. Parameter, 7dpf
N
Inter / Intra Inst. Acc.
Inter / Intra Circ.
CV of Beh. Parameter
Days
O
Inter / Intra Bursting
Inter / Intra Speed
CV of Beh. Parameter
Days
P
Inter / Intra Tort.
Inter / Intra Inst. Tort.
SD of Beh. Parameter
Days
Q
Inter
Intra
Density
CV of Activity, 5dpf
CV of Radial index, 5dpf
R
CV of Activity, 6dpf
CV of Radial index, 6dpf
S
CV of Activity, 7dpf
CV of Radial index, 7dpf
T
CV of Activity, 8dpf
CV of Radial index, 8dpf

Figure S2

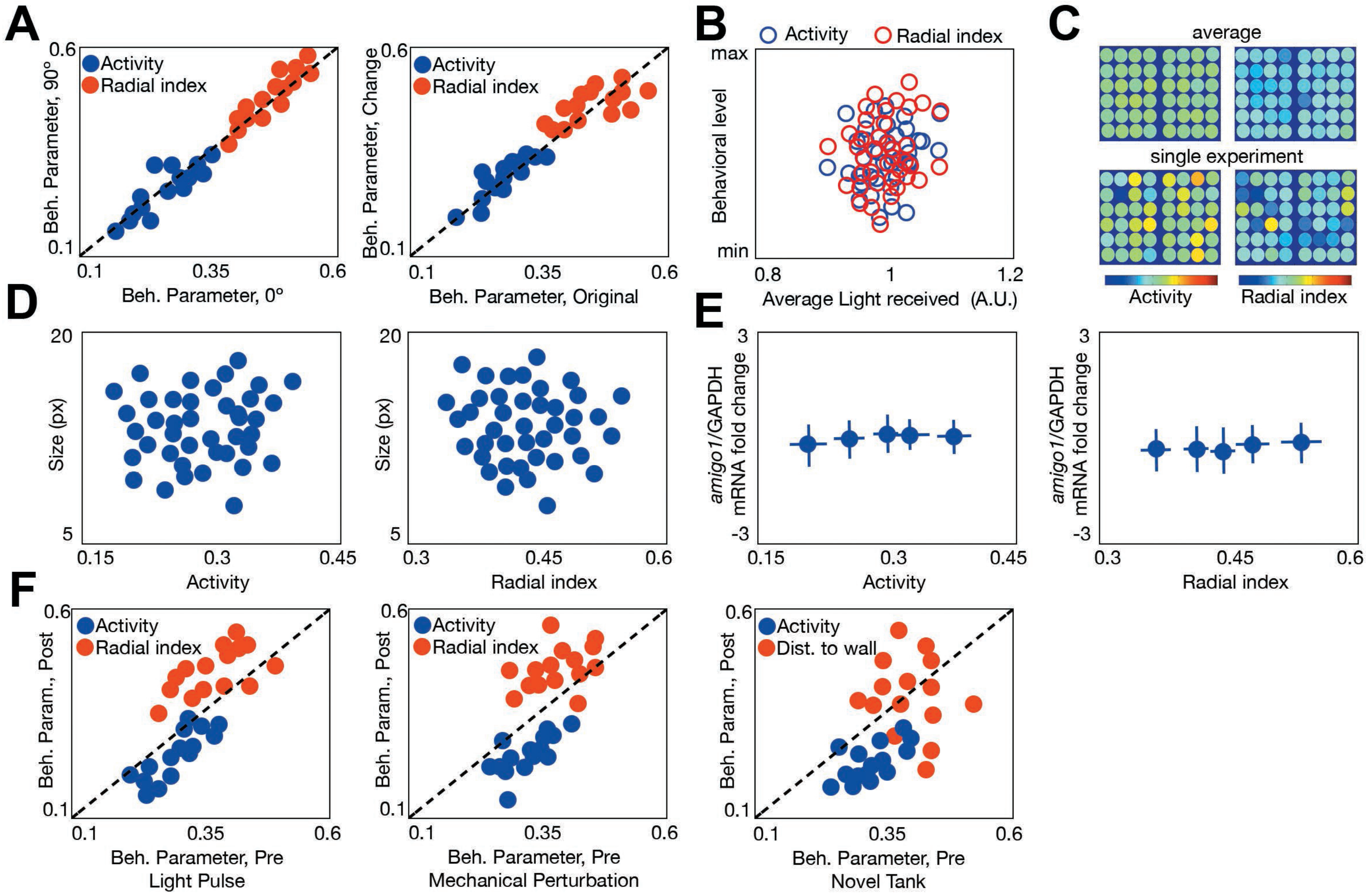
A
Activity
Radial index
Beh. Parameter, 90°
Beh. Parameter, 0°
Beh. Parameter, Change
Beh. Parameter, Original
B
Activity
Radial index
Behavioral level
max
min
Average Light received (A.U.)
C
average
single experiment
Activity
Radial index
D
Size (px)
Activity
Radial index
E
amigo1/GAPDH mRNA fold change
Activity
Radial index
F
Activity
Radial index
Dist. to wall
Beh. Param., Post
Beh. Parameter, Pre
Light Pulse
Mechanical Perturbation
Novel Tank

Figure S3

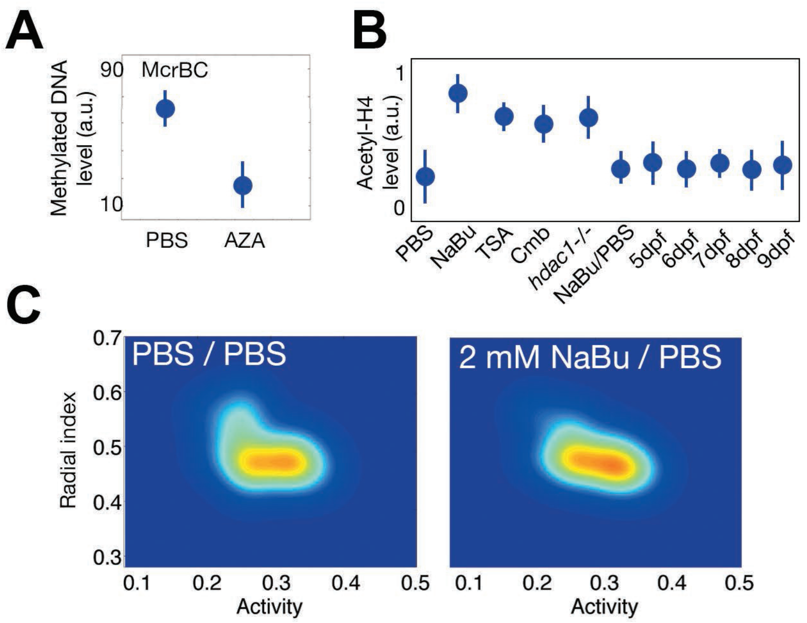
A
90
McrBC
Methylated DNA level (a.u.)
10
PBS
AZA
B
1
Acetyl-H4 level (a.u.)
0
PBS
NaBu
TSA
Cmb
hdac1-/-
NaBu/PBS
5dpf
6dpf
7dpf
8dpf
9dpf
C
0.7
0.6
0.5
0.4
0.3
Radial index
PBS / PBS
2 mM NaBu / PBS
0.1
0.2
0.3
0.4
0.5
Activity

Figure S4

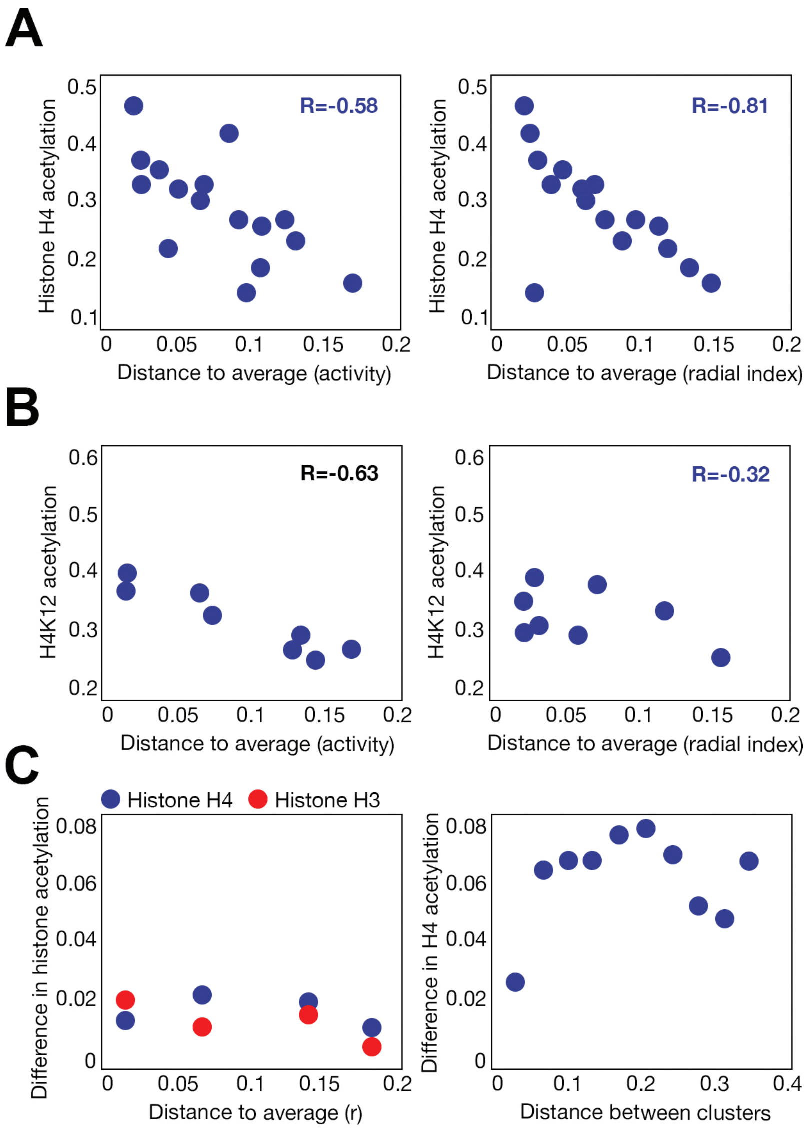
A
Histone H4 acetylation
R=-0.58
Distance to average (activity)
Histone H4 acetylation
R=-0.81
Distance to average (radial index)
B
H4K12 acetylation
R=-0.63
Distance to average (activity)
H4K12 acetylation
R=-0.32
Distance to average (radial index)
C
Histone H4
Histone H3
Difference in histone acetylation
Distance to average (r)
Difference in H4 acetylation
Distance between clusters

Figure S5

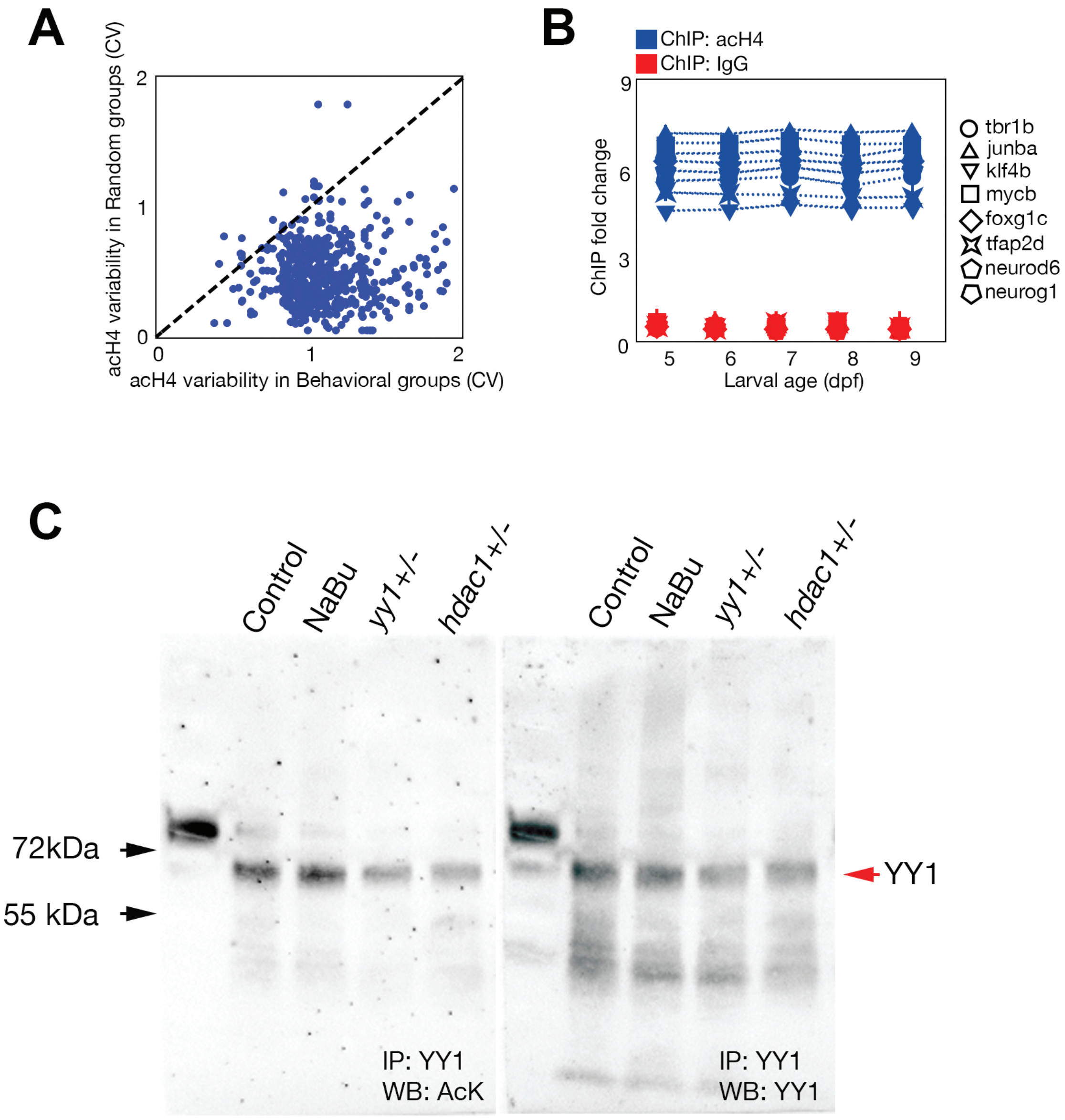
A
acH4 variability in Random groups (CV)
acH4 variability in Behavioral groups (CV)
B
ChIP: acH4
ChIP: IgG
ChIP fold change
Larval age (dpf)
tbr1b
junba
klf4b
mycb
foxg1c
tfap2d
neurod6
neurog1
C
Control
NaBu
yy1+/-
hdac1+/-
72kDa
55 kDa
YY1
IP: YY1
WB: AcK
IP: YY1
WB: YY1

Figure S6
