# Supplementary material for: Histone H4 acetylation regulates behavioral inter-individual variability in zebrafish
Source: Genome Biol. 2018 Apr 25;19:55. doi: 10.1186/s13059-018-1428-y (PMC5922312; doi:10.1186/s13059-018-1428-y)
Supplement: Supplementary file 2 — Tables S1–S7. (DOCX 208 kb) [file 13059_2018_1428_MOESM2_ESM.docx]

**Table S1.** Generalized Variance (GV) obtained for three replicas of each experimental condition.

| Sample | 10^5^GV  (rep1) | 10^5^ GV  (rep2) | 10^5^ GV  (rep3) |
| --- | --- | --- | --- |
| WIK F1 | 3.421 | 4.081 | 3.566 |
| WIK F3 | 3.123 | 3.444 | 3.765 |
| CG2 | 2.899 | 3.232 | 3.445 |
| LPS | 2.810 | 3.552 | 3.769 |
| No Food | 3.421 | 3.894 | 2.958 |
| No Water | 2.895 | 3.124 | 3.632 |
| PBS | 3.457 | 3.901 | 2.817 |
| AZA | 3.880 | 3.698 | 4.012 |
| NaBu | 0.714 | 0.532 | 0.410 |
| TSA | 1.141 | 1.012 | 0.878 |
| Cambinol | 2.581 | 2.467 | 3.412 |
| PBS/PBS (48h) | 2.981 | 3.244 | 3.665 |
| NaBu/PBS (48h) | 3.011 | 2.892 | 2.611 |
| *hdac1 +/+* | 3.235 | 4.002 | 4.025 |
| *hdac1 +/- (sa436)* | 0.600 | 0.491 | 0.450 |
| *hdac1 +/- (hi1612)* | 0.555 | 0.711 | 0.567 |
| *hdac1 +/- (hi1612*) NaBu | 0.681 | 0.540 | 0.701 |
| *yy1 +/+* | 4.221 | 3.981 | 3.347 |
| *yy1 +/-* | 0.711 | 1.010 | 0.991 |
| *yy1 +/-* NaBu | 0.812 | 0.981 | 1.102 |

**Table S2.** *P-*values obtained in the statistical comparisons of behavioral inter-individual variability using standard deviation (SD) and Coefficient of Variation (CV) of separated parameters.

| Experiment | SD *P*- value (activity) | SD *P*-value (radial index) | CV *P*-value (activity) | CV *P*-value (radial index) |
| --- | --- | --- | --- | --- |
| WIK F1 vs F3 | 0.73 | 0.13 | 0.43 | 0.24 |
| WIK F1 vs CG2 | 0.43 | 0.10 | 0.04 | 0.88 |
| WIK F1 vs LPS | 0.79 | 0.70 | 0.91 | 0.76 |
| Control Food vs No Food | 0.44 | 0.71 | 0.54 | 0.75 |
| Control Water vs No Water | 0.67 | 0.72 | 0.88 | 0.81 |
| PBS vs AZA | 0.31 | 0.55 | 0.42 | 0.52 |
| PBS vs NaBu | <0.001 | <0.001 | <0.001 | <0.001 |
| PBS vs NaBu (48h) | 0.73 | 0.77 | 0.71 | 0.61 |
| PBS vs TSA | 0.02 | 0.03 | 0.01 | 0.02 |
| PBS vs Cambinol | 0.56 | 0.52 | 0.81 | 0.75 |
| *hdac1 +/+* vs *hdac1 +/- (sa436)* | 0.54 | <0.001 | 0.68 | <0.001 |
| *hdac1 +/+* vs *hdac1 +/- (hi1612)* | 0.03 | <0.001 | 0.02 | <0.001 |
| *hdac1 +/-* vs *hdac1 +/- (hi1612)* 2 mM NaBu | 0.41 | 0.34 | 0.25 | 0.24 |
| *yy1 +/+* vs *yy1 +/-* | 0.02 | <0.001 | 0.011 | <0.001 |
| *yy1 +/-* vs *yy1 +/-* NaBu | 0.41 | 0.51 | 0.32 | 0.33 |

**Table S3.** Genomic coordinates of acetylated peaks obtained after comparison between control, NaBu-treated and random clusters of zebrafish larva. It also includes the nearest gene position for each region.

| **Chromosome** | **Origin** | **End** | **Nearest gene** | **Distance to TSS** |
| --- | --- | --- | --- | --- |
| 1 | 640727 | 640943 | ENSDARG00000070643 | -69312 |
| 1 | 8221437 | 8221634 | ENSDARG00000058464 | -395 |
| 1 | 11519800 | 11519987 | ENSDARG00000032808 | -27729 |
| 1 | 13398430 | 13398770 | ENSDARG00000039063 | -18993 |
| 1 | 13398891 | 13400141 | ENSDARG00000039063 | -17622 |
| 1 | 13482126 | 13482444 | ENSDARG00000088602 | -774 |
| 1 | 19872172 | 19872381 | ENSDARG00000095472 | 74938 |
| 1 | 20266211 | 20266489 | ENSDARG00000070173 | 73688 |
| 1 | 25510276 | 25510467 | ENSDARG00000079925 | 6636 |
| 1 | 28109739 | 28110038 | ENSDARG00000096648 | -4902 |
| 1 | 39982678 | 39982974 | ENSDARG00000005479 | -232274 |
| 1 | 40344941 | 40345149 | ENSDARG00000013042 | -31887 |
| 1 | 40469127 | 40469370 | ENSDARG00000093120 | -10126 |
| 1 | 40830171 | 40830504 | ENSDARG00000079552 | -81354 |
| 1 | 46396074 | 46396278 | ENSDARG00000016548 | -27 |
| 1 | 47146615 | 47146858 | ENSDARG00000036645 | -290 |
| 1 | 47160891 | 47161159 | ENSDARG00000036645 | 13743 |
| 1 | 47873499 | 47873821 | ENSDARG00000036687 | -44748 |
| 1 | 49860866 | 49861096 | ENSDARG00000008886 | -1567 |
| 1 | 51046345 | 51046576 | ENSDARG00000014098 | -423 |
| 1 | 51217949 | 51218287 | ENSDARG00000075564 | -81869 |
| 1 | 51989257 | 51989560 | ENSDARG00000002937 | -5323 |
| 1 | 52227873 | 52228181 | ENSDARG00000074378 | -295 |
| 1 | 54275140 | 54275451 | ENSDARG00000079973 | 32 |
| 1 | 60129914 | 60130172 | ENSDARG00000043471 | 61659 |
| 2 | 135455 | 135661 | ENSDARG00000088857 | -19516 |
| 2 | 14513319 | 14513634 | ENSDARG00000081482 | 15413 |
| 2 | 14531839 | 14533219 | ENSDARG00000080774 | 758 |
| 2 | 14555999 | 14556744 | ENSDARG00000085174 | 31316 |
| 2 | 18588503 | 18588674 | ENSDARG00000057253 | 23791 |
| 2 | 18594081 | 18594249 | ENSDARG00000057253 | 29369 |
| 2 | 18989859 | 18990110 | ENSDARG00000076183 | 6422 |
| 2 | 19004965 | 19005234 | ENSDARG00000076183 | 21528 |
| 2 | 19455798 | 19456081 | ENSDARG00000002412 | 167379 |
| 2 | 19612680 | 19613006 | ENSDARG00000089610 | 104553 |
| 2 | 21283455 | 21283746 | ENSDARG00000024759 | -403 |
| 2 | 21780112 | 21780414 | ENSDARG00000032317 | -676 |
| 2 | 21843979 | 21844305 | ENSDARG00000032317 | -64543 |
| 2 | 23481952 | 23482204 | ENSDARG00000003607 | -7637 |
| 2 | 24765613 | 24765953 | ENSDARG00000056262 | -77 |
| 2 | 30138298 | 30138630 | ENSDARG00000015863 | -38 |
| 2 | 31340370 | 31340564 | ENSDARG00000077219 | -10115 |
| 2 | 31735557 | 31735853 | ENSDARG00000007241 | -1426 |
| 2 | 32103592 | 32103929 | ENSDARG00000038780 | -1387 |
| 2 | 33045702 | 33045904 | ENSDARG00000038792 | -1305 |
| 2 | 35470956 | 35471243 | ENSDARG00000036257 | -16788 |
| 2 | 37565154 | 37565486 | ENSDARG00000079134 | -10397 |
| 2 | 38086597 | 38086871 | ENSDARG00000075445 | -10567 |
| 2 | 43733674 | 43733883 | ENSDARG00000060054 | -15697 |
| 2 | 49838945 | 49839168 | ENSDARG00000036626 | -36368 |
| 2 | 50712578 | 50712922 | ENSDARG00000052618 | -55644 |
| 2 | 51234691 | 51235028 | ENSDARG00000020794 | -1545 |
| 2 | 52051317 | 52051602 | ENSDARG00000094544 | 35837 |
| 2 | 53432550 | 53432778 | ENSDARG00000079556 | 55810 |
| 2 | 53760724 | 53760910 | ENSDARG00000087108 | 34361 |
| 2 | 56947911 | 56948168 | ENSDARG00000060763 | 2558 |
| 2 | 57776976 | 57777249 | ENSDARG00000005915 | -76496 |
| 2 | 57880039 | 57880346 | ENSDARG00000005915 | 26294 |
| 2 | 58759565 | 58759856 | ENSDARG00000079930 | -832 |
| 2 | 59136076 | 59136382 | ENSDARG00000068417 | 11015 |
| 2 | 59438798 | 59439048 | ENSDARG00000089523 | 40437 |
| 2 | 59522980 | 59523316 | ENSDARG00000067966 | -678 |
| 2 | 60081324 | 60081573 | ENSDARG00000089388 | -90330 |
| 2 | 60091133 | 60091472 | ENSDARG00000089388 | -80431 |
| 3 | 83548 | 83783 | ENSDARG00000059182 | -291 |
| 3 | 1982076 | 1982413 | ENSDARG00000080745 | 1687 |
| 3 | 5789023 | 5789253 | ENSDARG00000001014 | -37072 |
| 3 | 5806366 | 5806526 | ENSDARG00000001014 | -19799 |
| 3 | 9981802 | 9982105 | ENSDARG00000060976 | 97203 |
| 3 | 10695021 | 10695261 | ENSDARG00000058819 | -420 |
| 3 | 10826900 | 10827222 | ENSDARG00000087687 | -61 |
| 3 | 14464432 | 14464675 | ENSDARG00000061836 | -91375 |
| 3 | 15338421 | 15338704 | ENSDARG00000021242 | -420 |
| 3 | 22249172 | 22249479 | ENSDARG00000075428 | 35129 |
| 3 | 23145699 | 23145891 | ENSDARG00000038176 | 24921 |
| 3 | 24059847 | 24060140 | ENSDARG00000056012 | 124776 |
| 3 | 24065889 | 24066141 | ENSDARG00000056012 | 130818 |
| 3 | 25523827 | 25524143 | ENSDARG00000077361 | 1629 |
| 3 | 28250856 | 28251136 | ENSDARG00000014746 | -328181 |
| 3 | 31852326 | 31852632 | ENSDARG00000054362 | -58 |
| 3 | 36271865 | 36272562 | ENSDARG00000076128 | 34 |
| 3 | 36345156 | 36345489 | ENSDARG00000037924 | -18532 |
| 3 | 36944628 | 36944861 | ENSDARG00000074524 | 17379 |
| 3 | 40763006 | 40763328 | ENSDARG00000008383 | 27565 |
| 3 | 46708761 | 46709061 | ENSDARG00000032344 | -40838 |
| 3 | 46855003 | 46855280 | ENSDARG00000014420 | -689 |
| 3 | 46978411 | 46978732 | ENSDARG00000014420 | 122442 |
| 3 | 53813702 | 53813940 | ENSDARG00000078904 | -59516 |
| 3 | 57538058 | 57538382 | ENSDARG00000021846 | 14804 |
| 3 | 60951272 | 60951407 | ENSDARG00000019301 | -20784 |
| 3 | 61311012 | 61311280 | ENSDARG00000076968 | -26263 |
| 4 | 70385 | 70590 | ENSDARG00000045910 | -2300 |
| 4 | 1279795 | 1279990 | ENSDARG00000045798 | -278 |
| 4 | 2658761 | 2659041 | ENSDARG00000004227 | -80081 |
| 4 | 3181273 | 3181453 | ENSDARG00000074250 | 11878 |
| 4 | 9035420 | 9035599 | ENSDARG00000062968 | -61650 |
| 4 | 9035786 | 9036061 | ENSDARG00000062968 | -62016 |
| 4 | 9833104 | 9833467 | ENSDARG00000092922 | 183 |
| 4 | 13272037 | 13272226 | ENSDARG00000011661 | -51 |
| 4 | 14076847 | 14077177 | ENSDARG00000000019 | -114 |
| 4 | 14839878 | 14840122 | ENSDARG00000004336 | -168642 |
| 4 | 17660252 | 17660589 | ENSDARG00000005547 | 79518 |
| 4 | 19170941 | 19171169 | ENSDARG00000045482 | -425 |
| 4 | 21411186 | 21411700 | ENSDARG00000007523 | 2843 |
| 4 | 23806411 | 23806658 | ENSDARG00000045515 | 462047 |
| 4 | 27866624 | 27866814 | ENSDARG00000090824 | 122795 |
| 4 | 27867642 | 27867824 | ENSDARG00000090824 | 123813 |
| 4 | 27919363 | 27919565 | ENSDARG00000082539 | 81167 |
| 4 | 28001069 | 28003780 | ENSDARG00000090357 | 436 |
| 4 | 28007999 | 28009120 | ENSDARG00000090393 | 18 |
| 4 | 28010458 | 28010995 | ENSDARG00000091423 | 163 |
| 4 | 28011922 | 28012722 | ENSDARG00000087964 | 176 |
| 4 | 28013874 | 28017467 | ENSDARG00000086278 | 53991 |
| 4 | 29731641 | 29731906 | ENSDARG00000080902 | 7 |
| 4 | 30521452 | 30521634 | ENSDARG00000090209 | 104007 |
| 4 | 32423362 | 32423503 | ENSDARG00000089496 | 29 |
| 4 | 34960372 | 34960627 | ENSDARG00000096481 | 13555 |
| 4 | 35190190 | 35190513 | ENSDARG00000091953 | 28324 |
| 4 | 36627244 | 36627503 | ENSDARG00000079526 | 872 |
| 4 | 36789666 | 36789866 | ENSDARG00000086791 | -56997 |
| 4 | 39031234 | 39031920 | ENSDARG00000096075 | -9 |
| 4 | 40572332 | 40572564 | ENSDARG00000090166 | 42607 |
| 4 | 41319732 | 41319917 | ENSDARG00000094008 | 115089 |
| 4 | 41631020 | 41631289 | ENSDARG00000077886 | 33194 |
| 4 | 42663845 | 42664161 | ENSDARG00000091373 | 39402 |
| 4 | 42781585 | 42781752 | ENSDARG00000096441 | 9479 |
| 4 | 43268221 | 43268554 | ENSDARG00000079159 | -31126 |
| 4 | 43691169 | 43691372 | ENSDARG00000086984 | 22239 |
| 4 | 43892850 | 43893127 | ENSDARG00000090331 | 569 |
| 4 | 43901573 | 43903851 | ENSDARG00000090331 | 9292 |
| 4 | 43966440 | 43966710 | ENSDARG00000086606 | 18656 |
| 4 | 43972206 | 43972478 | ENSDARG00000086606 | 12888 |
| 4 | 44318109 | 44318331 | ENSDARG00000075856 | -9885 |
| 4 | 44662139 | 44662359 | ENSDARG00000086630 | 21078 |
| 4 | 45075391 | 45075588 | ENSDARG00000095526 | 49182 |
| 4 | 45562899 | 45563115 | ENSDARG00000087626 | 44248 |
| 4 | 45568444 | 45568651 | ENSDARG00000087626 | 49793 |
| 4 | 46219470 | 46219732 | ENSDARG00000096488 | 50827 |
| 4 | 46788001 | 46788200 | ENSDARG00000074067 | -212806 |
| 4 | 46819655 | 46819912 | ENSDARG00000080711 | 4306 |
| 4 | 48145612 | 48145734 | ENSDARG00000081707 | 262 |
| 4 | 49692764 | 49692973 | ENSDARG00000075315 | 8332 |
| 4 | 50855612 | 50855917 | ENSDARG00000090769 | 2946 |
| 4 | 52888317 | 52888520 | ENSDARG00000090160 | -93880 |
| 4 | 55932714 | 55932980 | ENSDARG00000091648 | -12609 |
| 4 | 56203112 | 56203361 | ENSDARG00000090400 | -11581 |
| 4 | 56437880 | 56438093 | ENSDARG00000096100 | -1294 |
| 4 | 57167325 | 57167619 | ENSDARG00000096206 | 16760 |
| 4 | 58105106 | 58105360 | ENSDARG00000092289 | 19462 |
| 4 | 59391963 | 59392141 | ENSDARG00000061751 | -5085 |
| 5 | 322979 | 323188 | ENSDARG00000089701 | -24745 |
| 5 | 588800 | 589027 | ENSDARG00000087450 | -24594 |
| 5 | 790395 | 790593 | ENSDARG00000059020 | 178 |
| 5 | 2723886 | 2724154 | ENSDARG00000089856 | -25571 |
| 5 | 3077292 | 3077478 | ENSDARG00000033143 | 27343 |
| 5 | 3352288 | 3352577 | ENSDARG00000060592 | 138325 |
| 5 | 3353008 | 3353243 | ENSDARG00000060592 | 137659 |
| 5 | 3368105 | 3368445 | ENSDARG00000060592 | 122457 |
| 5 | 4013701 | 4014001 | ENSDARG00000079244 | 128 |
| 5 | 4903471 | 4903777 | ENSDARG00000045983 | 84 |
| 5 | 7427286 | 7427512 | ENSDARG00000089882 | 16846 |
| 5 | 8817258 | 8817643 | ENSDARG00000084983 | 65183 |
| 5 | 12725283 | 12725489 | ENSDARG00000078272 | 125425 |
| 5 | 14555754 | 14556052 | ENSDARG00000027141 | -199 |
| 5 | 17121027 | 17121410 | ENSDARG00000042069 | 673 |
| 5 | 26189406 | 26189673 | ENSDARG00000035564 | -34 |
| 5 | 27394797 | 27395078 | ENSDARG00000035571 | -16474 |
| 5 | 27830037 | 27831019 | ENSDARG00000056477 | -627 |
| 5 | 31291368 | 31291572 | ENSDARG00000026048 | -365 |
| 5 | 32236086 | 32236318 | ENSDARG00000004851 | -592 |
| 5 | 33992249 | 33992450 | ENSDARG00000016319 | -9447 |
| 5 | 34533821 | 34534029 | ENSDARG00000035422 | -1602 |
| 5 | 37730303 | 37730533 | ENSDARG00000068731 | 158986 |
| 5 | 38039523 | 38039852 | ENSDARG00000094666 | 65784 |
| 5 | 38847513 | 38847847 | ENSDARG00000083229 | 2769 |
| 5 | 42943162 | 42943337 | ENSDARG00000067729 | -1797 |
| 5 | 42943381 | 42943604 | ENSDARG00000067729 | -2016 |
| 5 | 43387830 | 43388227 | ENSDARG00000059855 | 4721 |
| 5 | 44013622 | 44013944 | ENSDARG00000093543 | -6576 |
| 5 | 44101064 | 44101441 | ENSDARG00000089623 | 6949 |
| 5 | 44812468 | 44812612 | ENSDARG00000089337 | 861 |
| 5 | 44869942 | 44870202 | ENSDARG00000091494 | 863 |
| 5 | 47356060 | 47356207 | ENSDARG00000089310 | 52964 |
| 5 | 48885361 | 48885527 | ENSDARG00000093413 | 377713 |
| 5 | 49199787 | 49200074 | ENSDARG00000035535 | 445353 |
| 5 | 50263244 | 50263480 | ENSDARG00000081238 | 72397 |
| 5 | 51556255 | 51556538 | ENSDARG00000052697 | -265010 |
| 5 | 60067760 | 60067946 | ENSDARG00000052387 | -48796 |
| 5 | 60096637 | 60096915 | ENSDARG00000004864 | 50829 |
| 5 | 66575256 | 66575498 | ENSDARG00000093999 | 154 |
| 5 | 67371717 | 67371917 | ENSDARG00000059858 | -140 |
| 5 | 68442221 | 68442515 | ENSDARG00000028949 | -342 |
| 6 | 55069 | 55402 | ENSDARG00000052190 | 28912 |
| 6 | 75784 | 76120 | ENSDARG00000052192 | -3746 |
| 6 | 1574514 | 1574830 | ENSDARG00000078641 | 41144 |
| 6 | 1575698 | 1575861 | ENSDARG00000078641 | 42328 |
| 6 | 3321602 | 3321848 | ENSDARG00000059128 | -222 |
| 6 | 6269597 | 6269692 | ENSDARG00000078440 | 6791 |
| 6 | 6274461 | 6274722 | ENSDARG00000078440 | 11655 |
| 6 | 6275966 | 6277056 | ENSDARG00000078440 | 13160 |
| 6 | 6280957 | 6282087 | ENSDARG00000078440 | 18151 |
| 6 | 6282379 | 6283350 | ENSDARG00000078440 | 19573 |
| 6 | 6540422 | 6540637 | ENSDARG00000058874 | 38657 |
| 6 | 7166298 | 7166587 | ENSDARG00000071217 | 41364 |
| 6 | 7470002 | 7470244 | ENSDARG00000000103 | -102 |
| 6 | 14344638 | 14344976 | ENSDARG00000070995 | 180398 |
| 6 | 14830881 | 14831199 | ENSDARG00000044872 | 18811 |
| 6 | 17921237 | 17921467 | ENSDARG00000087317 | 30832 |
| 6 | 21846474 | 21846780 | ENSDARG00000026767 | 258306 |
| 6 | 24850620 | 24850882 | ENSDARG00000086914 | 32931 |
| 6 | 27962550 | 27962790 | ENSDARG00000070864 | 31757 |
| 6 | 28872873 | 28873767 | ENSDARG00000061362 | 15646 |
| 6 | 30194442 | 30194620 | ENSDARG00000061654 | -10945 |
| 6 | 30552458 | 30552777 | ENSDARG00000032868 | -322249 |
| 6 | 35467873 | 35468201 | ENSDARG00000070013 | 131788 |
| 6 | 35495776 | 35496017 | ENSDARG00000070013 | 103972 |
| 6 | 43023216 | 43023413 | ENSDARG00000060115 | -62 |
| 6 | 43535668 | 43535987 | ENSDARG00000014181 | -285775 |
| 6 | 44380850 | 44381033 | ENSDARG00000073869 | 47568 |
| 6 | 46368616 | 46368934 | ENSDARG00000044182 | 104 |
| 6 | 49210410 | 49211682 | ENSDARG00000014050 | 53983 |
| 6 | 49225658 | 49226269 | ENSDARG00000014050 | 69231 |
| 6 | 50692100 | 50692254 | ENSDARG00000075530 | 10048 |
| 6 | 53095684 | 53096012 | ENSDARG00000017294 | -355 |
| 6 | 53335131 | 53335469 | ENSDARG00000031855 | -24134 |
| 6 | 56113125 | 56113436 | ENSDARG00000040608 | 37877 |
| 6 | 59650543 | 59650852 | ENSDARG00000092148 | -556 |
| 6 | 59811151 | 59811406 | ENSDARG00000059775 | -387 |
| 7 | 518201 | 518576 | ENSDARG00000063150 | 21856 |
| 7 | 649511 | 649771 | ENSDARG00000021907 | 28009 |
| 7 | 1523246 | 1523493 | ENSDARG00000092488 | -17167 |
| 7 | 1592302 | 1592606 | ENSDARG00000089426 | 5877 |
| 7 | 1751762 | 1752070 | ENSDARG00000079949 | -62305 |
| 7 | 4182870 | 4183091 | ENSDARG00000011170 | -26667 |
| 7 | 4334589 | 4334900 | ENSDARG00000090645 | 16076 |
| 7 | 7251782 | 7252055 | ENSDARG00000088562 | 3913 |
| 7 | 7271170 | 7271434 | ENSDARG00000074012 | -23 |
| 7 | 10124814 | 10124994 | ENSDARG00000079027 | -17958 |
| 7 | 10827644 | 10827865 | ENSDARG00000075555 | 100096 |
| 7 | 11465164 | 11465407 | ENSDARG00000058449 | -81996 |
| 7 | 15246817 | 15247056 | ENSDARG00000016307 | 166396 |
| 7 | 15413510 | 15413809 | ENSDARG00000016307 | -58 |
| 7 | 18641304 | 18641583 | ENSDARG00000028333 | 14571 |
| 7 | 19942413 | 19942645 | ENSDARG00000036678 | 15145 |
| 7 | 22466954 | 22467138 | ENSDARG00000036445 | -25609 |
| 7 | 29589431 | 29589686 | ENSDARG00000094528 | 4191 |
| 7 | 29983752 | 29983939 | ENSDARG00000054423 | -304 |
| 7 | 31828308 | 31828477 | ENSDARG00000017431 | 1715 |
| 7 | 33370635 | 33370931 | ENSDARG00000089236 | -13850 |
| 7 | 35509952 | 35510262 | ENSDARG00000036096 | 15997 |
| 7 | 35510481 | 35510657 | ENSDARG00000036096 | 16526 |
| 7 | 35921465 | 35923110 | ENSDARG00000077584 | 19331 |
| 7 | 36714112 | 36714393 | ENSDARG00000053049 | -13835 |
| 7 | 38002616 | 38003011 | ENSDARG00000073957 | 288676 |
| 7 | 38371718 | 38372042 | ENSDARG00000074319 | 397395 |
| 7 | 45598772 | 45598949 | ENSDARG00000008811 | -2601 |
| 7 | 47883765 | 47883949 | ENSDARG00000089284 | 47882 |
| 7 | 54454289 | 54454570 | ENSDARG00000035852 | 31961 |
| 7 | 54482108 | 54482270 | ENSDARG00000035852 | 59780 |
| 7 | 54484556 | 54484801 | ENSDARG00000035852 | 62228 |
| 7 | 56781930 | 56782216 | ENSDARG00000051854 | -148 |
| 7 | 60872894 | 60873143 | ENSDARG00000051955 | -183 |
| 7 | 64241003 | 64241211 | ENSDARG00000056389 | 55652 |
| 7 | 64247933 | 64251332 | ENSDARG00000056389 | 45531 |
| 7 | 65188934 | 65189158 | ENSDARG00000041337 | 170372 |
| 7 | 65237956 | 65238194 | ENSDARG00000041337 | 121336 |
| 7 | 72069374 | 72069694 | ENSDARG00000079268 | 4634 |
| 7 | 74871956 | 74872128 | ENSDARG00000089847 | -31262 |
| 7 | 75774608 | 75774838 | ENSDARG00000090326 | 19239 |
| 8 | 263052 | 263244 | ENSDARG00000043028 | -21911 |
| 8 | 458375 | 458639 | ENSDARG00000013375 | -10088 |
| 8 | 1080883 | 1081210 | ENSDARG00000058553 | 52917 |
| 8 | 2142414 | 2142650 | ENSDARG00000080000 | 3353 |
| 8 | 8304784 | 8305066 | ENSDARG00000076494 | 3549 |
| 8 | 10149204 | 10149591 | ENSDARG00000007172 | 33860 |
| 8 | 10638886 | 10639101 | ENSDARG00000058944 | 45994 |
| 8 | 11504977 | 11505253 | ENSDARG00000074168 | -278 |
| 8 | 16930502 | 16930707 | ENSDARG00000057610 | -3218 |
| 8 | 19741853 | 19742100 | ENSDARG00000095763 | 224 |
| 8 | 20314782 | 20314952 | ENSDARG00000062889 | 127144 |
| 8 | 22072619 | 22072913 | ENSDARG00000038655 | 54080 |
| 8 | 24633182 | 24633441 | ENSDARG00000042899 | 8483 |
| 8 | 26431445 | 26431719 | ENSDARG00000075793 | 8582 |
| 8 | 34725377 | 34725554 | ENSDARG00000089760 | 93781 |
| 8 | 35160470 | 35160695 | ENSDARG00000091039 | 103588 |
| 8 | 35467958 | 35469812 | ENSDARG00000094733 | 3150 |
| 8 | 35925810 | 35926146 | ENSDARG00000077244 | 27667 |
| 8 | 36384500 | 36384627 | ENSDARG00000055441 | 97502 |
| 8 | 36401930 | 36402146 | ENSDARG00000055441 | 114932 |
| 8 | 36424598 | 36424949 | ENSDARG00000075277 | 123066 |
| 8 | 36433961 | 36434272 | ENSDARG00000075277 | 113743 |
| 8 | 36439108 | 36439386 | ENSDARG00000075277 | 108629 |
| 8 | 41101281 | 41101778 | ENSDARG00000082688 | 67673 |
| 8 | 45074836 | 45075083 | ENSDARG00000029865 | -35461 |
| 8 | 45700950 | 45701200 | ENSDARG00000041705 | 57366 |
| 8 | 45708744 | 45709141 | ENSDARG00000041705 | 49425 |
| 8 | 45715401 | 45715600 | ENSDARG00000041705 | 42966 |
| 8 | 45718047 | 45718651 | ENSDARG00000041705 | 39915 |
| 8 | 45745698 | 45745863 | ENSDARG00000041705 | 12703 |
| 8 | 47164208 | 47164357 | ENSDARG00000053345 | 25140 |
| 8 | 51529427 | 51529664 | ENSDARG00000073996 | -35782 |
| 8 | 51565185 | 51565376 | ENSDARG00000073996 | -70 |
| 8 | 51883965 | 51884193 | ENSDARG00000059885 | -78555 |
| 8 | 54764327 | 54764495 | ENSDARG00000018030 | 17224 |
| 8 | 56044495 | 56044774 | ENSDARG00000014495 | -127726 |
| 8 | 56068856 | 56069171 | ENSDARG00000014495 | -152087 |
| 9 | 875525 | 875804 | ENSDARG00000075641 | 3786 |
| 9 | 3279033 | 3279271 | ENSDARG00000034916 | -90 |
| 9 | 3668210 | 3668527 | ENSDARG00000093411 | 18501 |
| 9 | 10684038 | 10684228 | ENSDARG00000079375 | 88633 |
| 9 | 15172794 | 15173095 | ENSDARG00000034555 | -311266 |
| 9 | 20852548 | 20852837 | ENSDARG00000093419 | 1965 |
| 9 | 21707967 | 21708247 | ENSDARG00000041728 | -120442 |
| 9 | 22376563 | 22376778 | ENSDARG00000010281 | -36318 |
| 9 | 22522020 | 22522286 | ENSDARG00000005576 | -35 |
| 9 | 25411363 | 25411549 | ENSDARG00000051824 | -34804 |
| 9 | 26447056 | 26447373 | ENSDARG00000011188 | -29066 |
| 9 | 26688055 | 26688295 | ENSDARG00000062338 | 120866 |
| 9 | 28084796 | 28084989 | ENSDARG00000093542 | 249233 |
| 9 | 28901894 | 28902195 | ENSDARG00000056923 | 69 |
| 9 | 32740937 | 32741271 | ENSDARG00000001835 | -39143 |
| 9 | 33391352 | 33391657 | ENSDARG00000092179 | -316 |
| 9 | 33966165 | 33966353 | ENSDARG00000069383 | -2235 |
| 9 | 34261514 | 34261715 | ENSDARG00000055833 | 8437 |
| 9 | 35193425 | 35193754 | ENSDARG00000024478 | -124 |
| 9 | 38807334 | 38807635 | ENSDARG00000069242 | -183 |
| 9 | 40128038 | 40128216 | ENSDARG00000020028 | -48606 |
| 9 | 40794819 | 40795123 | ENSDARG00000078874 | 176211 |
| 9 | 42150773 | 42150986 | ENSDARG00000009267 | 2086 |
| 9 | 45237983 | 45238297 | ENSDARG00000079397 | -117276 |
| 9 | 45248404 | 45248681 | ENSDARG00000079397 | -106892 |
| 9 | 45899702 | 45900000 | ENSDARG00000018383 | -287 |
| 9 | 52494733 | 52495025 | ENSDARG00000018553 | -97 |
| 9 | 52868767 | 52868967 | ENSDARG00000004712 | 85841 |
| 9 | 55584821 | 55585052 | ENSDARG00000074153 | 62786 |
| 9 | 57597232 | 57597523 | ENSDARG00000060401 | -9302 |
| 9 | 58115249 | 58115535 | ENSDARG00000042939 | -266 |
| 10 | 227692 | 227932 | ENSDARG00000018308 | -84634 |
| 10 | 468776 | 469003 | ENSDARG00000074329 | -268 |
| 10 | 2488513 | 2488833 | ENSDARG00000090886 | 67004 |
| 10 | 3435743 | 3435906 | ENSDARG00000020334 | -47 |
| 10 | 4208825 | 4209051 | ENSDARG00000010729 | 243041 |
| 10 | 5029140 | 5029422 | ENSDARG00000026198 | -42 |
| 10 | 6275832 | 6275985 | ENSDARG00000095948 | -27091 |
| 10 | 15788975 | 15789228 | ENSDARG00000052935 | 5522 |
| 10 | 16749051 | 16749396 | ENSDARG00000079729 | 65286 |
| 10 | 16751230 | 16752665 | ENSDARG00000079729 | 62017 |
| 10 | 19030148 | 19030341 | ENSDARG00000027638 | -6458 |
| 10 | 29606467 | 29606701 | ENSDARG00000084058 | 1053 |
| 10 | 33982987 | 33983206 | ENSDARG00000078459 | -128374 |
| 10 | 34046547 | 34046759 | ENSDARG00000078459 | -64821 |
| 10 | 37055740 | 37055999 | ENSDARG00000007856 | 97998 |
| 10 | 38755127 | 38755644 | ENSDARG00000095693 | 64597 |
| 10 | 38756175 | 38756333 | ENSDARG00000095693 | 65645 |
| 10 | 38807684 | 38807974 | ENSDARG00000033901 | 25487 |
| 10 | 38900974 | 38901192 | ENSDARG00000033901 | -67513 |
| 10 | 39892624 | 39892960 | ENSDARG00000085673 | 21400 |
| 10 | 40265146 | 40265410 | ENSDARG00000024865 | -149205 |
| 10 | 40495002 | 40495327 | ENSDARG00000077180 | -316 |
| 10 | 41486286 | 41486534 | ENSDARG00000075383 | 46907 |
| 10 | 42815094 | 42815316 | ENSDARG00000033411 | 161 |
| 10 | 45109596 | 45109915 | ENSDARG00000029764 | -65714 |
| 10 | 45557529 | 45557725 | ENSDARG00000068507 | 27998 |
| 11 | 427188 | 427465 | ENSDARG00000059404 | -118 |
| 11 | 2575234 | 2575394 | ENSDARG00000078076 | 2875 |
| 11 | 4152242 | 4152504 | ENSDARG00000029474 | -36787 |
| 11 | 5480044 | 5480258 | ENSDARG00000045006 | -441727 |
| 11 | 6158986 | 6159274 | ENSDARG00000003165 | -8755 |
| 11 | 7376248 | 7376531 | ENSDARG00000074848 | 2049 |
| 11 | 8368440 | 8368637 | ENSDARG00000058683 | -1582 |
| 11 | 11961539 | 11961832 | ENSDARG00000058663 | 6955 |
| 11 | 12521536 | 12521871 | ENSDARG00000095491 | 17305 |
| 11 | 12630910 | 12631174 | ENSDARG00000090197 | 1005 |
| 11 | 12640218 | 12640556 | ENSDARG00000088474 | 2877 |
| 11 | 12652750 | 12653058 | ENSDARG00000094884 | -1225 |
| 11 | 12674577 | 12674829 | ENSDARG00000091337 | 11008 |
| 11 | 12736400 | 12736678 | ENSDARG00000086624 | 986 |
| 11 | 21292594 | 21292929 | ENSDARG00000074853 | 4978 |
| 11 | 23860911 | 23861137 | ENSDARG00000044902 | -154212 |
| 11 | 31239940 | 31240267 | ENSDARG00000055976 | -34 |
| 11 | 32003248 | 32003630 | ENSDARG00000075030 | 11703 |
| 11 | 33499907 | 33500106 | ENSDARG00000027041 | -35395 |
| 11 | 35541054 | 35541227 | ENSDARG00000055736 | 6977 |
| 11 | 36602175 | 36602440 | ENSDARG00000004060 | -1672 |
| 11 | 39788571 | 39788809 | ENSDARG00000078473 | 287 |
| 12 | 463900 | 464153 | ENSDARG00000074997 | 13422 |
| 12 | 2618528 | 2618735 | ENSDARG00000042197 | 5069 |
| 12 | 3724577 | 3724844 | ENSDARG00000035809 | -386 |
| 12 | 5163255 | 5164150 | ENSDARG00000075022 | -3356 |
| 12 | 5164151 | 5165074 | ENSDARG00000075022 | -2432 |
| 12 | 5165186 | 5165772 | ENSDARG00000075022 | -1734 |
| 12 | 8193427 | 8193662 | ENSDARG00000096601 | -51190 |
| 12 | 11520436 | 11520685 | ENSDARG00000071691 | -62 |
| 12 | 12387729 | 12387972 | ENSDARG00000024398 | -24838 |
| 12 | 15330825 | 15330986 | ENSDARG00000055679 | -10026 |
| 12 | 17420511 | 17420737 | ENSDARG00000061194 | -31704 |
| 12 | 23235539 | 23235821 | ENSDARG00000052654 | 46144 |
| 12 | 27224210 | 27224541 | ENSDARG00000003008 | -56078 |
| 12 | 27771078 | 27771407 | ENSDARG00000077749 | -118060 |
| 12 | 28420613 | 28420888 | ENSDARG00000013207 | -1869 |
| 12 | 28487681 | 28487908 | ENSDARG00000061311 | -39269 |
| 12 | 30297523 | 30297742 | ENSDARG00000004473 | -173 |
| 12 | 32680346 | 32680686 | ENSDARG00000004415 | -109731 |
| 12 | 36687820 | 36688141 | ENSDARG00000080009 | -3054 |
| 12 | 42237515 | 42237835 | ENSDARG00000074386 | -122007 |
| 12 | 43373686 | 43373916 | ENSDARG00000025241 | -46630 |
| 12 | 45972264 | 45972543 | ENSDARG00000039667 | 109 |
| 12 | 50658814 | 50659040 | ENSDARG00000075883 | 331 |
| 13 | 3382714 | 3382919 | ENSDARG00000002026 | -63632 |
| 13 | 5654822 | 5655081 | ENSDARG00000070076 | -417 |
| 13 | 5718618 | 5718834 | ENSDARG00000030804 | 45411 |
| 13 | 10685165 | 10685411 | ENSDARG00000078947 | -46654 |
| 13 | 10708480 | 10708707 | ENSDARG00000078947 | -69969 |
| 13 | 10769874 | 10770157 | ENSDARG00000063400 | -305517 |
| 13 | 13522194 | 13522464 | ENSDARG00000056598 | -1173 |
| 13 | 13531400 | 13531662 | ENSDARG00000056616 | -1396 |
| 13 | 17311517 | 17311749 | ENSDARG00000057166 | -201878 |
| 13 | 19163931 | 19164398 | ENSDARG00000076088 | -11356 |
| 13 | 19215244 | 19215562 | ENSDARG00000054611 | 5237 |
| 13 | 19223143 | 19223404 | ENSDARG00000054611 | 13136 |
| 13 | 19224915 | 19225063 | ENSDARG00000054611 | 14908 |
| 13 | 22696031 | 22696192 | ENSDARG00000019728 | -28 |
| 13 | 25306402 | 25306609 | ENSDARG00000039429 | -116541 |
| 13 | 26000668 | 26000890 | ENSDARG00000019845 | 2595 |
| 13 | 26124854 | 26125094 | ENSDARG00000061352 | -47919 |
| 13 | 28009513 | 28009776 | ENSDARG00000083500 | 9244 |
| 13 | 28900063 | 28900320 | ENSDARG00000011862 | -775 |
| 13 | 29647091 | 29647395 | ENSDARG00000006356 | -661 |
| 13 | 30563426 | 30563645 | ENSDARG00000074502 | 81429 |
| 13 | 31870136 | 31870315 | ENSDARG00000004695 | -610 |
| 13 | 32901216 | 32901470 | ENSDARG00000012563 | -48310 |
| 13 | 45432383 | 45432720 | ENSDARG00000005010 | -23299 |
| 13 | 46542392 | 46542641 | ENSDARG00000052623 | 4929 |
| 13 | 48578512 | 48578636 | ENSDARG00000088229 | 65907 |
| 14 | 789996 | 790281 | ENSDARG00000091277 | -485 |
| 14 | 3103384 | 3103683 | ENSDARG00000062379 | 5793 |
| 14 | 3570362 | 3570635 | ENSDARG00000034011 | 41280 |
| 14 | 4171770 | 4172052 | ENSDARG00000088264 | -50264 |
| 14 | 6912862 | 6913160 | ENSDARG00000089372 | 2724 |
| 14 | 19504681 | 19504964 | ENSDARG00000095422 | 135172 |
| 14 | 21106088 | 21107907 | ENSDARG00000037433 | 49837 |
| 14 | 24592129 | 24592437 | ENSDARG00000018989 | -214 |
| 14 | 25562319 | 25562564 | ENSDARG00000034268 | -198261 |
| 14 | 26963462 | 26963762 | ENSDARG00000019353 | -2651 |
| 14 | 27713334 | 27713625 | ENSDARG00000056130 | -303 |
| 14 | 28998284 | 28998477 | ENSDARG00000034871 | 362032 |
| 14 | 29002420 | 29002635 | ENSDARG00000034871 | 357874 |
| 14 | 29825318 | 29825532 | ENSDARG00000034610 | 237044 |
| 14 | 47607760 | 47608086 | ENSDARG00000030638 | -48011 |
| 14 | 50880239 | 50880513 | ENSDARG00000005482 | -86422 |
| 14 | 51395525 | 51395755 | ENSDARG00000060018 | -606 |
| 15 | 1802783 | 1802974 | ENSDARG00000059029 | -8158 |
| 15 | 3515414 | 3515624 | ENSDARG00000078683 | 107874 |
| 15 | 4492405 | 4492577 | ENSDARG00000042252 | 16677 |
| 15 | 5636115 | 5636443 | ENSDARG00000087043 | 21720 |
| 15 | 6199876 | 6200105 | ENSDARG00000028118 | -102695 |
| 15 | 8009981 | 8010283 | ENSDARG00000062633 | 54532 |
| 15 | 9137227 | 9137440 | ENSDARG00000012968 | -33 |
| 15 | 9709859 | 9710180 | ENSDARG00000091042 | 195794 |
| 15 | 13424512 | 13424643 | ENSDARG00000034058 | 17789 |
| 15 | 13448660 | 13448931 | ENSDARG00000034058 | -6228 |
| 15 | 19844694 | 19844995 | ENSDARG00000028259 | 43289 |
| 15 | 20333888 | 20334178 | ENSDARG00000081501 | 65097 |
| 15 | 22540835 | 22541145 | ENSDARG00000026753 | -320113 |
| 15 | 23118779 | 23119029 | ENSDARG00000084048 | 2880 |
| 15 | 26385667 | 26385883 | ENSDARG00000090764 | -108847 |
| 15 | 26427428 | 26427700 | ENSDARG00000090764 | -150608 |
| 15 | 28905326 | 28905618 | ENSDARG00000040984 | -233 |
| 15 | 29016156 | 29016383 | ENSDARG00000068965 | -4661 |
| 15 | 31094992 | 31095259 | ENSDARG00000013921 | -41962 |
| 15 | 33130731 | 33131026 | ENSDARG00000061211 | 54534 |
| 15 | 33239305 | 33239571 | ENSDARG00000062183 | 8650 |
| 15 | 36573477 | 36573768 | ENSDARG00000012381 | 24097 |
| 15 | 44376239 | 44376553 | ENSDARG00000088574 | -14514 |
| 15 | 45784572 | 45784907 | ENSDARG00000017217 | 71 |
| 15 | 47184847 | 47185072 | ENSDARG00000091508 | 67124 |
| 16 | 43517 | 43768 | ENSDARG00000022122 | -9150 |
| 16 | 352999 | 353262 | ENSDARG00000009510 | 467379 |
| 16 | 3798340 | 3799085 | ENSDARG00000080370 | 12573 |
| 16 | 7257479 | 7257703 | ENSDARG00000004176 | -99340 |
| 16 | 7436450 | 7436749 | ENSDARG00000014571 | -22238 |
| 16 | 9762116 | 9762451 | ENSDARG00000035308 | -17675 |
| 16 | 11739747 | 11739987 | ENSDARG00000074974 | -88527 |
| 16 | 11791293 | 11791651 | ENSDARG00000074974 | -36863 |
| 16 | 14975971 | 14976205 | ENSDARG00000070620 | -164037 |
| 16 | 15635350 | 15635512 | ENSDARG00000069545 | -57 |
| 16 | 26332847 | 26333029 | ENSDARG00000090190 | -41837 |
| 16 | 26920520 | 26920733 | ENSDARG00000040287 | -50 |
| 16 | 27484892 | 27485174 | ENSDARG00000040278 | 414 |
| 16 | 27606635 | 27606842 | ENSDARG00000073705 | -487 |
| 16 | 27983551 | 27983861 | ENSDARG00000087832 | 4939 |
| 16 | 28183109 | 28183281 | ENSDARG00000095944 | 47099 |
| 16 | 30992368 | 30992603 | ENSDARG00000086126 | -107 |
| 16 | 31788335 | 31788560 | ENSDARG00000095888 | -2093 |
| 16 | 31818892 | 31819228 | ENSDARG00000061644 | -71 |
| 16 | 34647154 | 34647414 | ENSDARG00000074691 | 27904 |
| 16 | 42028503 | 42028728 | ENSDARG00000040027 | -90735 |
| 16 | 46270418 | 46270609 | ENSDARG00000060489 | 9193 |
| 16 | 47877709 | 47877925 | ENSDARG00000039913 | 385 |
| 16 | 57182685 | 57183053 | ENSDARG00000078486 | 50336 |
| 16 | 58215281 | 58215450 | ENSDARG00000052717 | 26023 |
| 16 | 58334475 | 58334741 | ENSDARG00000021891 | -205 |
| 17 | 759198 | 759506 | ENSDARG00000037840 | -6998 |
| 17 | 12248228 | 12248567 | ENSDARG00000058117 | -59 |
| 17 | 14990062 | 14990369 | ENSDARG00000070453 | -145 |
| 17 | 17965523 | 17965735 | ENSDARG00000062510 | -18012 |
| 17 | 19456197 | 19456475 | ENSDARG00000017569 | -35 |
| 17 | 22952733 | 22952947 | ENSDARG00000077864 | -598 |
| 17 | 25796757 | 25796991 | ENSDARG00000091139 | 90648 |
| 17 | 27182426 | 27182680 | ENSDARG00000055270 | -59022 |
| 17 | 29961857 | 29962161 | ENSDARG00000004861 | 560 |
| 17 | 31777902 | 31778130 | ENSDARG00000036775 | -51 |
| 17 | 34970987 | 34972046 | ENSDARG00000055283 | 981 |
| 17 | 36361051 | 36361386 | ENSDARG00000077811 | 302282 |
| 17 | 37361865 | 37362081 | ENSDARG00000076501 | -458 |
| 17 | 37684059 | 37684343 | ENSDARG00000007198 | -5449 |
| 17 | 40118976 | 40119093 | ENSDARG00000013134 | 70176 |
| 17 | 40120163 | 40120422 | ENSDARG00000013134 | 71363 |
| 17 | 40125813 | 40125947 | ENSDARG00000013134 | 77013 |
| 17 | 40126453 | 40126606 | ENSDARG00000013134 | 77653 |
| 17 | 40127044 | 40127327 | ENSDARG00000013134 | 78244 |
| 17 | 42073546 | 42073774 | ENSDARG00000079753 | 34069 |
| 17 | 46607461 | 46607722 | ENSDARG00000090064 | 62659 |
| 17 | 46609107 | 46609397 | ENSDARG00000090064 | 60984 |
| 17 | 46611801 | 46611983 | ENSDARG00000090064 | 58398 |
| 17 | 46616339 | 46616652 | ENSDARG00000090064 | 53729 |
| 17 | 46621414 | 46621641 | ENSDARG00000090064 | 48740 |
| 17 | 46627293 | 46627442 | ENSDARG00000090064 | 42939 |
| 17 | 46628029 | 46628185 | ENSDARG00000090064 | 42196 |
| 17 | 46630135 | 46630441 | ENSDARG00000090064 | 39940 |
| 17 | 46633670 | 46633874 | ENSDARG00000090064 | 36507 |
| 17 | 46634822 | 46635074 | ENSDARG00000090064 | 35307 |
| 17 | 46636187 | 46636289 | ENSDARG00000090064 | 34092 |
| 17 | 46636517 | 46636788 | ENSDARG00000090064 | 33593 |
| 17 | 46642762 | 46642927 | ENSDARG00000090064 | 27454 |
| 17 | 46919642 | 46919936 | ENSDARG00000087539 | 33373 |
| 17 | 46920215 | 46920422 | ENSDARG00000087539 | 33946 |
| 17 | 47990852 | 47991193 | ENSDARG00000033426 | 20040 |
| 17 | 47998287 | 47999180 | ENSDARG00000033426 | 27475 |
| 17 | 48008450 | 48008757 | ENSDARG00000033426 | 37638 |
| 17 | 53981942 | 53982210 | ENSDARG00000091001 | 29626 |
| 18 | 41102 | 42308 | ENSDARG00000074354 | 24782 |
| 18 | 138120 | 138351 | ENSDARG00000036232 | -23639 |
| 18 | 943741 | 943959 | ENSDARG00000061685 | 56 |
| 18 | 1197683 | 1198011 | ENSDARG00000018020 | 9639 |
| 18 | 12215988 | 12216273 | ENSDARG00000002249 | -111674 |
| 18 | 12608613 | 12608874 | ENSDARG00000058857 | -1935 |
| 18 | 15289056 | 15289378 | ENSDARG00000057728 | 5865 |
| 18 | 16314635 | 16314917 | ENSDARG00000057167 | 60115 |
| 18 | 20417061 | 20417372 | ENSDARG00000083973 | 2427 |
| 18 | 20667115 | 20667270 | ENSDARG00000027423 | -161021 |
| 18 | 20705828 | 20706016 | ENSDARG00000027423 | -122275 |
| 18 | 20899407 | 20899654 | ENSDARG00000041081 | -5480 |
| 18 | 23285806 | 23286039 | ENSDARG00000093393 | 315215 |
| 18 | 23660968 | 23661167 | ENSDARG00000040926 | -145541 |
| 18 | 29816405 | 29816673 | ENSDARG00000043640 | 31771 |
| 18 | 29996164 | 29996437 | ENSDARG00000007976 | 25196 |
| 18 | 30011481 | 30011750 | ENSDARG00000007976 | 40513 |
| 18 | 30055695 | 30055978 | ENSDARG00000095204 | 18071 |
| 18 | 33103107 | 33103313 | ENSDARG00000087263 | 205473 |
| 18 | 38259746 | 38260078 | ENSDARG00000090322 | 60785 |
| 18 | 39198103 | 39198419 | ENSDARG00000068380 | -379 |
| 18 | 44778685 | 44778846 | ENSDARG00000005468 | 716 |
| 18 | 48957073 | 48957325 | ENSDARG00000076973 | 18813 |
| 19 | 606646 | 606918 | ENSDARG00000042637 | -43 |
| 19 | 2544808 | 2545055 | ENSDARG00000011870 | -1156 |
| 19 | 3081258 | 3081524 | ENSDARG00000026762 | -55206 |
| 19 | 4037352 | 4037591 | ENSDARG00000079528 | -21987 |
| 19 | 4132103 | 4132250 | ENSDARG00000071205 | -12528 |
| 19 | 6199295 | 6199549 | ENSDARG00000059067 | -96377 |
| 19 | 6912228 | 6912448 | ENSDARG00000036816 | -12945 |
| 19 | 8072661 | 8072883 | ENSDARG00000071026 | -316 |
| 19 | 11017694 | 11017964 | ENSDARG00000079847 | -255 |
| 19 | 12478685 | 12479010 | ENSDARG00000075405 | -43 |
| 19 | 13271995 | 13272266 | ENSDARG00000070553 | -109 |
| 19 | 20613304 | 20613658 | ENSDARG00000036222 | 16858 |
| 19 | 30853619 | 30853910 | ENSDARG00000060073 | -54862 |
| 19 | 36535737 | 36535969 | ENSDARG00000032849 | 7540 |
| 19 | 36899088 | 36899320 | ENSDARG00000028533 | 10701 |
| 19 | 43323700 | 43323914 | ENSDARG00000061231 | -18752 |
| 19 | 43608158 | 43608402 | ENSDARG00000070151 | -531 |
| 19 | 44607350 | 44607655 | ENSDARG00000023160 | -47 |
| 19 | 46606019 | 46606220 | ENSDARG00000089491 | 124894 |
| 19 | 47521374 | 47521603 | ENSDARG00000068028 | -27241 |
| 19 | 49300736 | 49301069 | ENSDARG00000031511 | 11175 |
| 20 | 3120061 | 3120381 | ENSDARG00000005416 | -79 |
| 20 | 3578761 | 3579342 | ENSDARG00000058801 | 137336 |
| 20 | 4645302 | 4645677 | ENSDARG00000037066 | 189559 |
| 20 | 5025724 | 5026043 | ENSDARG00000092503 | -68463 |
| 20 | 7558368 | 7558626 | ENSDARG00000074185 | 15224 |
| 20 | 10254201 | 10254504 | ENSDARG00000070786 | 1985 |
| 20 | 15921836 | 15922082 | ENSDARG00000008573 | -40819 |
| 20 | 17060870 | 17061035 | ENSDARG00000062750 | 48055 |
| 20 | 17634298 | 17634598 | ENSDARG00000018693 | 151422 |
| 20 | 19973544 | 19973702 | ENSDARG00000039577 | 37253 |
| 20 | 19993426 | 19993550 | ENSDARG00000039577 | 57135 |
| 20 | 20427697 | 20427957 | ENSDARG00000029569 | 30745 |
| 20 | 20914207 | 20914473 | ENSDARG00000043257 | 20258 |
| 20 | 21035805 | 21036104 | ENSDARG00000005002 | -61854 |
| 20 | 24307024 | 24307811 | ENSDARG00000090703 | 2095 |
| 20 | 25741630 | 25741879 | ENSDARG00000002991 | -305 |
| 20 | 26922916 | 26923165 | ENSDARG00000055431 | 6187 |
| 20 | 28435997 | 28436204 | ENSDARG00000038348 | 1928 |
| 20 | 29812879 | 29813103 | ENSDARG00000029544 | -15 |
| 20 | 32577962 | 32578234 | ENSDARG00000017266 | -15505 |
| 20 | 33470227 | 33470429 | ENSDARG00000095565 | 12310 |
| 20 | 34620662 | 34620885 | ENSDARG00000054723 | -52689 |
| 20 | 34987058 | 34987398 | ENSDARG00000004735 | 95844 |
| 20 | 35907799 | 35908126 | ENSDARG00000015224 | -25792 |
| 20 | 43213417 | 43213660 | ENSDARG00000029472 | -163912 |
| 20 | 47198815 | 47199073 | ENSDARG00000083952 | 40820 |
| 20 | 48410507 | 48410844 | ENSDARG00000023272 | -1009 |
| 20 | 48788053 | 48788281 | ENSDARG00000091756 | -855 |
| 20 | 52034097 | 52034338 | ENSDARG00000095420 | 13145 |
| 20 | 54392355 | 54392680 | ENSDARG00000041239 | -66 |
| 20 | 54887493 | 54887818 | ENSDARG00000068912 | -264 |
| 20 | 55063703 | 55064030 | ENSDARG00000079437 | -9462 |
| 20 | 55477876 | 55478286 | ENSDARG00000087034 | 28407 |
| 21 | 3707107 | 3707304 | ENSDARG00000016858 | 17738 |
| 21 | 5747728 | 5747841 | ENSDARG00000079472 | 14924 |
| 21 | 7525176 | 7525490 | ENSDARG00000014201 | -3434 |
| 21 | 16084552 | 16084821 | ENSDARG00000011459 | -29946 |
| 21 | 19093402 | 19093739 | ENSDARG00000011671 | 90594 |
| 21 | 20013664 | 20014060 | ENSDARG00000057089 | 7038 |
| 21 | 23760136 | 23760389 | ENSDARG00000078435 | 53565 |
| 21 | 24459418 | 24459631 | ENSDARG00000056248 | 5310 |
| 21 | 24944992 | 24945296 | ENSDARG00000089836 | -154 |
| 21 | 28013777 | 28013990 | ENSDARG00000077818 | -117725 |
| 21 | 30468093 | 30468358 | ENSDARG00000011171 | 136846 |
| 21 | 34865229 | 34865540 | ENSDARG00000090470 | 2331 |
| 21 | 43709392 | 43711147 | ENSDARG00000075038 | -20049 |
| 21 | 43724218 | 43726019 | ENSDARG00000075038 | -5177 |
| 21 | 43729614 | 43732697 | ENSDARG00000076417 | 36069 |
| 22 | 757360 | 757670 | ENSDARG00000091131 | -11695 |
| 22 | 986760 | 986996 | ENSDARG00000044525 | -8732 |
| 22 | 1490316 | 1490618 | ENSDARG00000092088 | -27 |
| 22 | 6468859 | 6469243 | ENSDARG00000095952 | 24746 |
| 22 | 11973497 | 11973751 | ENSDARG00000062865 | 3963 |
| 22 | 15452207 | 15452519 | ENSDARG00000094089 | 3192 |
| 22 | 16664857 | 16665175 | ENSDARG00000071458 | 6605 |
| 22 | 17152683 | 17152997 | ENSDARG00000062420 | -121356 |
| 22 | 18078417 | 18078743 | ENSDARG00000016188 | 94010 |
| 22 | 18179990 | 18180133 | ENSDARG00000005783 | -66376 |
| 22 | 18339279 | 18339558 | ENSDARG00000056162 | 40529 |
| 22 | 20282168 | 20282432 | ENSDARG00000071375 | -47583 |
| 22 | 21565938 | 21566197 | ENSDARG00000056530 | 10922 |
| 22 | 22779854 | 22780090 | ENSDARG00000062173 | 131 |
| 22 | 23289248 | 23289483 | ENSDARG00000082557 | 152665 |
| 22 | 23290165 | 23290415 | ENSDARG00000082557 | 151733 |
| 22 | 25757309 | 25757644 | ENSDARG00000087583 | 4186 |
| 22 | 31470813 | 31471170 | ENSDARG00000093790 | 17239 |
| 22 | 35218282 | 35218495 | ENSDARG00000024276 | -159282 |
| 22 | 37552651 | 37552943 | ENSDARG00000018944 | 8342 |
| 22 | 38568551 | 38568846 | ENSDARG00000086824 | 48819 |
| 22 | 41849015 | 41849246 | ENSDARG00000070898 | 2243 |
| 22 | 42208182 | 42208516 | ENSDARG00000087502 | 51021 |
| 22 | 42253385 | 42253498 | ENSDARG00000087502 | 6039 |
| 22 | 42253514 | 42255013 | ENSDARG00000087502 | 4524 |
| 23 | 3860285 | 3860527 | ENSDARG00000078369 | 46107 |
| 23 | 7748117 | 7748431 | ENSDARG00000008953 | 17176 |
| 23 | 10139186 | 10139453 | ENSDARG00000075821 | -104758 |
| 23 | 10165974 | 10166205 | ENSDARG00000075821 | -78006 |
| 23 | 14526849 | 14527043 | ENSDARG00000095139 | -160 |
| 23 | 15147504 | 15147723 | ENSDARG00000038737 | -110 |
| 23 | 19270293 | 19270535 | ENSDARG00000008434 | -514 |
| 23 | 21757527 | 21757772 | ENSDARG00000009822 | -158 |
| 23 | 22365341 | 22365556 | ENSDARG00000056695 | -42041 |
| 23 | 22817174 | 22817459 | ENSDARG00000095377 | 52907 |
| 23 | 24200375 | 24200668 | ENSDARG00000012340 | 4636 |
| 23 | 25438443 | 25438756 | ENSDARG00000023648 | 15999 |
| 23 | 27309358 | 27309573 | ENSDARG00000037285 | -496 |
| 23 | 27981833 | 27982085 | ENSDARG00000037062 | -604 |
| 23 | 29143680 | 29143914 | ENSDARG00000028322 | -91786 |
| 23 | 31483971 | 31484272 | ENSDARG00000036878 | 159804 |
| 23 | 31585050 | 31585237 | ENSDARG00000015970 | 84 |
| 23 | 31892396 | 31892620 | ENSDARG00000025522 | -39268 |
| 23 | 33436084 | 33436350 | ENSDARG00000080504 | 3285 |
| 23 | 34359346 | 34359591 | ENSDARG00000003206 | -411 |
| 23 | 36245035 | 36245238 | ENSDARG00000093797 | -56297 |
| 23 | 36293376 | 36293590 | ENSDARG00000093797 | -7945 |
| 23 | 36313918 | 36314240 | ENSDARG00000093797 | 12383 |
| 23 | 44402357 | 44403176 | ENSDARG00000060597 | 2836 |
| 23 | 45863203 | 45863521 | ENSDARG00000095817 | -31 |
| 23 | 46147183 | 46147365 | ENSDARG00000043046 | -10314 |
| 24 | 3649894 | 3650147 | ENSDARG00000071823 | -210831 |
| 24 | 3701407 | 3701630 | ENSDARG00000071823 | -159348 |
| 24 | 4771580 | 4771805 | ENSDARG00000045444 | 243624 |
| 24 | 5770547 | 5770868 | ENSDARG00000055629 | -26310 |
| 24 | 12301044 | 12301276 | ENSDARG00000053943 | 74401 |
| 24 | 12550657 | 12550943 | ENSDARG00000005220 | -40597 |
| 24 | 13684935 | 13685242 | ENSDARG00000014259 | 143957 |
| 24 | 16166418 | 16167242 | ENSDARG00000093743 | 187170 |
| 24 | 16819834 | 16820217 | ENSDARG00000006331 | 56043 |
| 24 | 16821466 | 16821661 | ENSDARG00000006331 | 57675 |
| 24 | 16822144 | 16822414 | ENSDARG00000006331 | 58353 |
| 24 | 16826069 | 16826225 | ENSDARG00000006331 | 62278 |
| 24 | 16828196 | 16828414 | ENSDARG00000006331 | 64405 |
| 24 | 16828435 | 16828591 | ENSDARG00000006331 | 64644 |
| 24 | 22282709 | 22282912 | ENSDARG00000029177 | -32 |
| 24 | 23149342 | 23149659 | ENSDARG00000062415 | -108509 |
| 24 | 32591296 | 32591497 | ENSDARG00000091489 | 72778 |
| 24 | 43044219 | 43044408 | ENSDARG00000043260 | 45121 |
| 24 | 43089524 | 43089827 | ENSDARG00000061417 | 6063 |
| 25 | 238315 | 238586 | ENSDARG00000046157 | 5443 |
| 25 | 3801480 | 3801788 | ENSDARG00000028539 | -21 |
| 25 | 5148178 | 5148518 | ENSDARG00000014311 | 53933 |
| 25 | 7039630 | 7039938 | ENSDARG00000078227 | 57193 |
| 25 | 8777315 | 8777536 | ENSDARG00000080594 | 17948 |
| 25 | 10758519 | 10758828 | ENSDARG00000046030 | -148 |
| 25 | 10849953 | 10850293 | ENSDARG00000013379 | 8899 |
| 25 | 12262819 | 12263058 | ENSDARG00000077228 | -87087 |
| 25 | 12693726 | 12694020 | ENSDARG00000087569 | 54778 |
| 25 | 12738246 | 12738565 | ENSDARG00000087569 | 10233 |
| 25 | 14639169 | 14639487 | ENSDARG00000033596 | -25 |
| 25 | 15091547 | 15091770 | ENSDARG00000023927 | 38950 |
| 25 | 17529028 | 17529226 | ENSDARG00000051748 | -66612 |
| 25 | 17601372 | 17601667 | ENSDARG00000018559 | -31 |
| 25 | 21098774 | 21099052 | ENSDARG00000091807 | 68562 |
| 25 | 22114397 | 22114597 | ENSDARG00000004925 | -63442 |
| 25 | 22169112 | 22169322 | ENSDARG00000004925 | -8717 |
| 25 | 22386907 | 22387122 | ENSDARG00000025346 | -94 |
| 25 | 25948911 | 25949247 | ENSDARG00000040854 | 80790 |
| 25 | 25997369 | 25998006 | ENSDARG00000040854 | 129248 |
| 25 | 31162173 | 31162511 | ENSDARG00000082458 | 3592 |
| 25 | 33089103 | 33089316 | ENSDARG00000079374 | 47184 |
| 25 | 35543858 | 35544038 | ENSDARG00000086667 | -68 |
| 25 | 35600486 | 35600711 | ENSDARG00000045556 | -9399 |
| 25 | 37867513 | 37867732 | ENSDARG00000082003 | 3800 |
| 25 | 38408632 | 38408782 | ENSDARG00000009563 | -34100 |

**Table S4**. Top (1,000) up-regulated transcripts after NaBu treatment

| **Transcript ID** | **Control TMM mean** | **NaBu TMM mean** | **Noiseq probability** |
| --- | --- | --- | --- |
| "ENSDART00000063272" | 439.720133244289 | 1228.40309996996 | 1 |
| "ENSDART00000081039" | 494.847988829128 | 1485.52252142729 | 1 |
| "ENSDART00000151384" | 208.668743674141 | 542.722835374951 | 0.999999999999996 |
| "ENSDART00000034549" | 3940.94559032737 | 8568.59445428911 | 0.999999999999978 |
| "ENSDART00000053382" | 184.64124489636 | 483.24820583253 | 0.999999999999957 |
| "ENSDART00000091158" | 114.127744357428 | 903.663252761797 | 0.999999999999955 |
| "ENSDART00000136995" | 338.266819267461 | 850.60320139908 | 0.999999999999946 |
| "ENSDART00000042061" | 718.20408250092 | 1111.75926437469 | 0.999999999999943 |
| "ENSDART00000022139" | 4530.87958811026 | 5820.92843079452 | 0.998781624534592 |
| "ENSDART00000102474" | 117.068518261684 | 298.147396573344 | 0.997270748638578 |
| "ENSDART00000008893" | 326.478443337549 | 519.785997309986 | 0.986033013691634 |
| "ENSDART00000024558" | 7091.85171124689 | 11530.1065601771 | 0.983375251042619 |
| "ENSDART00000136313" | 324.31922539934 | 767.754278937086 | 0.964803170429033 |
| "ENSDART00000074463" | 2041.1595655866 | 2604.12300683451 | 0.956581104695291 |
| "ENSDART00000146616" | 32.1241860329662 | 75.9924373614258 | 0.955620668345276 |
| "ENSDART00000135364" | 822.792396778794 | 1188.83231458432 | 0.951183453280671 |
| "ENSDART00000082457" | 68.4705030238843 | 132.937540033553 | 0.94852856940446 |
| "ENSDART00000137554" | 153.875647732371 | 634.793699346207 | 0.946237111590033 |
| "ENSDART00000115190" | 15.0540660047175 | 22.6684903392305 | 0.930025273560465 |
| "ENSDART00000109722" | 10.4117170073089 | 17.0810879116566 | 0.930025226706717 |
| "ENSDART00000144220" | 63.8051611299393 | 85.3091567452957 | 0.930023002403552 |
| "ENSDART00000147385" | 189.465025015557 | 229.517952337678 | 0.930017966606106 |
| "ENSDART00000073907" | 36.5209490170969 | 60.3689188297897 | 0.930017686205221 |
| "ENSDART00000115260" | 324.649579209864 | 387.558675183501 | 0.93001711420425 |
| "ENSDART00000156678" | 10.3969969790923 | 17.0887326000662 | 0.930015761369186 |
| "ENSDART00000051271" | 141.137146019488 | 172.783331920391 | 0.930015482937815 |
| "ENSDART00000126328" | 10.1178839976172 | 16.7832318014328 | 0.930015385135598 |
| "ENSDART00000115071" | 301.668902554527 | 377.907719868009 | 0.93001299791978 |
| "ENSDART00000112014" | 121.667378030937 | 151.938048567966 | 0.93001284770689 |
| "ENSDART00000109327" | 11.1332710234911 | 18.0097717945312 | 0.930012294631353 |
| "ENSDART00000125630" | 465.226139018269 | 529.683850740675 | 0.930011881211341 |
| "ENSDART00000136816" | 51.2250140831015 | 78.7443347334433 | 0.930011666162607 |
| "ENSDART00000108899" | 8.2874470524298 | 17.0751157020346 | 0.93001062798892 |
| "ENSDART00000122113" | 20.8494580157811 | 31.3988146698501 | 0.930000501261739 |
| "ENSDART00000111986" | 200.42592521621 | 236.725556294932 | 0.929998432606256 |
| "ENSDART00000137948" | 38.1205860070843 | 53.7379121207903 | 0.929995306691218 |
| "ENSDART00000077734" | 178.192591216643 | 213.483095959493 | 0.929993178542817 |
| "ENSDART00000143007" | 89.9030891667807 | 116.938213919503 | 0.929992722649969 |
| "ENSDART00000086248" | 28.9547370600676 | 43.1581017805613 | 0.929991075267192 |
| "ENSDART00000099105" | 7950.81675829872 | 8299.27080946148 | 0.929971842224369 |
| "ENSDART00000086018" | 206.035799193722 | 246.040362881115 | 0.929970873397159 |
| "ENSDART00000148871" | 89.7627540100849 | 113.757305642139 | 0.929966720357149 |
| "ENSDART00000155401" | 21.8723810236195 | 31.2836604547379 | 0.929965779539294 |
| "ENSDART00000036630" | 24.3795080289146 | 35.0141692520231 | 0.929941952273204 |
| "ENSDART00000132734" | 8.35833500966764 | 15.4749839178066 | 0.929933289198105 |
| "ENSDART00000134624" | 40.3372960579577 | 55.5345997658918 | 0.929912207942369 |
| "ENSDART00000157143" | 10.4192529892734 | 15.8019866217806 | 0.929905087728806 |
| "ENSDART00000092290" | 80.7722739756024 | 107.408213886883 | 0.92989922814443 |
| "ENSDART00000131846" | 20.8977380271255 | 29.1649875420254 | 0.929886897642689 |
| "ENSDART00000123521" | 11.6092720413261 | 20.4196109023168 | 0.929884976007414 |
| "ENSDART00000135252" | 20.548089024125 | 31.2664582802317 | 0.929874651706454 |
| "ENSDART00000091835" | 104.648250197878 | 133.985374868777 | 0.929862378830629 |
| "ENSDART00000055810" | 689.181807509023 | 762.45708405377 | 0.929859253684255 |
| "ENSDART00000152473" | 407.030066478969 | 482.491069559257 | 0.929852291951987 |
| "ENSDART00000155247" | 132.375673246713 | 167.98563299679 | 0.92983318850832 |
| "ENSDART00000146965" | 792.200732026621 | 926.437427813521 | 0.929792644966378 |
| "ENSDART00000084355" | 43.1646320098201 | 57.6661751222761 | 0.92978838915465 |
| "ENSDART00000007829" | 17.1447759764688 | 26.6290045731426 | 0.929781878441323 |
| "ENSDART00000113926" | 936.027368935828 | 1042.98283915693 | 0.929727933185993 |
| "ENSDART00000103010" | 46.705634060188 | 61.0699215344865 | 0.929720178375185 |
| "ENSDART00000144941" | 9.35900200732494 | 15.542357204774 | 0.929710778606894 |
| "ENSDART00000045474" | 10.8356700228172 | 16.4536018454122 | 0.929691229081083 |
| "ENSDART00000010626" | 258.522842123112 | 307.570835350183 | 0.929669563366358 |
| "ENSDART00000150058" | 822.526077028562 | 944.270986599321 | 0.929659074180469 |
| "ENSDART00000126726" | 8.13165498116218 | 15.1955250742472 | 0.929628824211083 |
| "ENSDART00000049885" | 325.222745077021 | 389.149012413902 | 0.929626240762115 |
| "ENSDART00000127473" | 26.2691770226808 | 36.3236122278074 | 0.929613596890471 |
| "ENSDART00000123544" | 602.367548202459 | 695.036035868113 | 0.929604523275156 |
| "ENSDART00000156522" | 32.7827069946568 | 47.6875685743183 | 0.929596079309226 |
| "ENSDART00000149736" | 306.884951958803 | 360.226926249837 | 0.929595703839958 |
| "ENSDART00000073583" | 2389.63597648792 | 2650.63470694911 | 0.929574957405565 |
| "ENSDART00000006867" | 1659.79418698256 | 1773.63665513588 | 0.92957065021819 |
| "ENSDART00000079293" | 11834.0559669982 | 13649.3697695651 | 0.929552781606633 |
| "ENSDART00000110389" | 591.402578619386 | 663.973593990626 | 0.929521483552514 |
| "ENSDART00000064274" | 50.1103878656242 | 74.5669513231375 | 0.929509979720112 |
| "ENSDART00000155575" | 25.0493659784826 | 37.5702219663579 | 0.92950619260327 |
| "ENSDART00000134054" | 378.522308345877 | 427.689654553452 | 0.929410360534445 |
| "ENSDART00000139258" | 9.32544202419714 | 14.5857185691385 | 0.929400190633786 |
| "ENSDART00000099425" | 12300.4748632883 | 14397.6426895798 | 0.929384018859207 |
| "ENSDART00000155264" | 4396.18357580704 | 5103.18105546059 | 0.929378837399991 |
| "ENSDART00000147609" | 385.251280403273 | 439.98850406019 | 0.92935644082924 |
| "ENSDART00000130274" | 14.9612739730109 | 23.900047768692 | 0.929331144597717 |
| "ENSDART00000137152" | 14.0051189957158 | 21.7560506064342 | 0.929327553727896 |
| "ENSDART00000010543" | 629.668829363127 | 710.962730939933 | 0.929301696040967 |
| "ENSDART00000034534" | 134.685879200718 | 165.70378787309 | 0.929278828142284 |
| "ENSDART00000145223" | 75.7836590155325 | 97.0263948185848 | 0.929264002055229 |
| "ENSDART00000153500" | 10.7760860385261 | 17.6538628157063 | 0.929237744644548 |
| "ENSDART00000156545" | 63.0658559809136 | 83.4771099779228 | 0.929216327982476 |
| "ENSDART00000129096" | 16.5047100190464 | 24.863136000579 | 0.929204757204904 |
| "ENSDART00000129593" | 151.793327203076 | 183.327546070618 | 0.929182310035485 |
| "ENSDART00000143676" | 26.7715869923221 | 39.0685761146061 | 0.929147162242076 |
| "ENSDART00000136142" | 24.9856290525662 | 37.8281789045768 | 0.929129039761214 |
| "ENSDART00000153793" | 16.8023110197204 | 26.6837157353374 | 0.929121925954652 |
| "ENSDART00000151167" | 76.3596361113262 | 97.3873212337728 | 0.929098624311237 |
| "ENSDART00000122909" | 65.7325430484585 | 85.5864657234379 | 0.929058185449614 |
| "ENSDART00000101640" | 9.71241900130775 | 15.2335114485652 | 0.929047456045833 |
| "ENSDART00000133274" | 22.6904950624906 | 32.2775677591627 | 0.929043866794879 |
| "ENSDART00000148829" | 47.9590050875141 | 64.2271780410926 | 0.929017116028754 |
| "ENSDART00000064332" | 14.6263449982269 | 22.657978079875 | 0.928971505485631 |
| "ENSDART00000156224" | 82.2074610753594 | 107.070224788093 | 0.928954309234106 |
| "ENSDART00000138468" | 8.14637500937879 | 16.420392588558 | 0.928950745504929 |
| "ENSDART00000061481" | 9040.9423883274 | 10435.2143733318 | 0.928915721399604 |
| "ENSDART00000084009" | 43.4920250026396 | 60.8055117488472 | 0.928896957575639 |
| "ENSDART00000123153" | 1527.39597181982 | 1641.92688622655 | 0.928893467085922 |
| "ENSDART00000003338" | 109.205728096187 | 134.68620643161 | 0.928880697578385 |
| "ENSDART00000145007" | 18.997116996125 | 26.676308114658 | 0.92887378227464 |
| "ENSDART00000008199" | 27.764333057372 | 42.4620473351914 | 0.928855045936602 |
| "ENSDART00000081494" | 127.648035184632 | 164.911282724039 | 0.928805074743015 |
| "ENSDART00000156873" | 54.8033441075795 | 74.1897775067023 | 0.928775308760386 |
| "ENSDART00000026576" | 1282.0908263934 | 1478.0231966059 | 0.928724405339498 |
| "ENSDART00000074883" | 127.124458026877 | 154.14989640382 | 0.928699789434517 |
| "ENSDART00000099049" | 463.478663904552 | 541.994002640502 | 0.928693067056381 |
| "ENSDART00000123458" | 159.013306212375 | 194.951434827733 | 0.928683888063177 |
| "ENSDART00000144405" | 52.602090971278 | 72.3527133029664 | 0.928683113224584 |
| "ENSDART00000042478" | 38.0870260239565 | 51.1730228770565 | 0.928650688311978 |
| "ENSDART00000083605" | 146.575001109874 | 180.675757106962 | 0.928646453007396 |
| "ENSDART00000155031" | 29.0889769925788 | 40.6073068214887 | 0.928603310744409 |
| "ENSDART00000007798" | 227.926921127461 | 272.353117328538 | 0.928596312896448 |
| "ENSDART00000136136" | 31.5625440147462 | 43.0869061493891 | 0.928559917791639 |
| "ENSDART00000022348" | 94.1633510150591 | 119.086033425966 | 0.928544943826034 |
| "ENSDART00000105541" | 419.568682065797 | 478.525942847406 | 0.928534086555282 |
| "ENSDART00000109181" | 55.9608589836414 | 84.9126968148043 | 0.928495327205547 |
| "ENSDART00000136542" | 13.6777260028963 | 19.2310572835364 | 0.928493477194654 |
| "ENSDART00000149640" | 18.5358360065066 | 32.9602391230625 | 0.928486531614454 |
| "ENSDART00000023265" | 9.99153198278282 | 16.4101206481017 | 0.928446253098354 |
| "ENSDART00000109829" | 512.99219339212 | 598.075095036471 | 0.92839793089706 |
| "ENSDART00000114215" | 131.211964101675 | 167.35385045088 | 0.928362271819228 |
| "ENSDART00000137244" | 10.292900974439 | 17.9940050310824 | 0.928353012998864 |
| "ENSDART00000088465" | 217.34166091604 | 265.972732365283 | 0.928340312111109 |
| "ENSDART00000081387" | 20.8049459954189 | 30.4438485130022 | 0.928298787579352 |
| "ENSDART00000046297" | 233.547329176633 | 290.182643338141 | 0.928251348681126 |
| "ENSDART00000063337" | 25.8078960330625 | 40.2986981330102 | 0.928184449821345 |
| "ENSDART00000140531" | 124.817987048924 | 153.766272991134 | 0.928157955840957 |
| "ENSDART00000138093" | 29.2036730987542 | 45.1747599895637 | 0.92814929723062 |
| "ENSDART00000144471" | 8.22444701286877 | 14.2235970617923 | 0.928140437169091 |
| "ENSDART00000114384" | 379.594314281276 | 433.933515247989 | 0.9281258829693 |
| "ENSDART00000062893" | 11.6055040503439 | 19.5375128554288 | 0.928085359578001 |
| "ENSDART00000064150" | 56.5998691339267 | 76.6722444055486 | 0.928084225293515 |
| "ENSDART00000144241" | 465.262494744448 | 552.40294378653 | 0.928007310200208 |
| "ENSDART00000152921" | 13.6219100095874 | 19.5172060422465 | 0.927977238948328 |
| "ENSDART00000008328" | 461.639652699607 | 525.973240546979 | 0.927906740057417 |
| "ENSDART00000156494" | 23.8250500569467 | 34.7672019597892 | 0.927843756944573 |
| "ENSDART00000032498" | 681.968143599899 | 750.250553410574 | 0.927807109497125 |
| "ENSDART00000146348" | 88.4135928613559 | 128.275957169077 | 0.927775102429163 |
| "ENSDART00000150555" | 14.8492570357502 | 23.8978979032748 | 0.92775765303703 |
| "ENSDART00000143883" | 15.4225549626293 | 24.1900188716069 | 0.927757295631243 |
| "ENSDART00000053518" | 214.437524386753 | 260.881714130023 | 0.927753196130271 |
| "ENSDART00000149275" | 134.36222118395 | 164.390999939731 | 0.927752268022747 |
| "ENSDART00000148681" | 20.4963929575107 | 31.3431487343964 | 0.927731938188352 |
| "ENSDART00000011568" | 813.023323809272 | 936.91531891253 | 0.927715651746501 |
| "ENSDART00000136684" | 20.4179690183083 | 28.8320126284295 | 0.927703861893325 |
| "ENSDART00000137093" | 299.356738832378 | 369.096362811093 | 0.927702389192759 |
| "ENSDART00000134790" | 31.6666400193995 | 43.4557175718859 | 0.927621409800872 |
| "ENSDART00000125709" | 84.1522421910098 | 113.346751426987 | 0.927556163989643 |
| "ENSDART00000048753" | 964.155809118276 | 1063.83798949138 | 0.927542797224053 |
| "ENSDART00000054651" | 12.6137070299656 | 19.5341678978535 | 0.927536806286038 |
| "ENSDART00000101633" | 221.931225024767 | 268.501544781217 | 0.927507749784521 |
| "ENSDART00000103703" | 18.7070519774155 | 29.8390594442565 | 0.927474317002896 |
| "ENSDART00000089050" | 126.920418959196 | 162.500179347059 | 0.927467909623298 |
| "ENSDART00000153177" | 49.306289983545 | 68.9231652545811 | 0.92738919932577 |
| "ENSDART00000142736" | 9.39974603670488 | 16.4729538853355 | 0.927378170577452 |
| "ENSDART00000080441" | 73.8491260656918 | 94.8415437110632 | 0.927326171772005 |
| "ENSDART00000075598" | 36.6103580084642 | 54.7222651902875 | 0.927305775511379 |
| "ENSDART00000153826" | 119.733197016808 | 158.719091227719 | 0.927305202043823 |
| "ENSDART00000144106" | 8.96107299297996 | 15.1785632186402 | 0.927278121543927 |
| "ENSDART00000078675" | 297.82398644707 | 356.656485024863 | 0.927276668882318 |
| "ENSDART00000149077" | 12.8852840294762 | 19.8396686964869 | 0.927231293221377 |
| "ENSDART00000100722" | 30.6886139825662 | 45.0257447379358 | 0.927193138423526 |
| "ENSDART00000088986" | 29.096161038831 | 38.7504131912 | 0.927174119281248 |
| "ENSDART00000150301" | 9.96927597260173 | 17.0681854679847 | 0.927150082879045 |
| "ENSDART00000111101" | 85.8724879073594 | 113.558836983579 | 0.927115543053298 |
| "ENSDART00000156396" | 15.8687639883187 | 25.7029479423147 | 0.927097544188503 |
| "ENSDART00000137620" | 1320.0340661467 | 1529.72757139599 | 0.927063045251663 |
| "ENSDART00000151072" | 15.894787989482 | 28.6407112716492 | 0.927033501862797 |
| "ENSDART00000136818" | 45.091661992627 | 61.1415945522882 | 0.927001433199433 |
| "ENSDART00000127721" | 23.1778000532723 | 40.3111264412215 | 0.926977163239004 |
| "ENSDART00000155728" | 9.35523401634271 | 14.2396041441403 | 0.926943309402987 |
| "ENSDART00000077217" | 32.8645800040596 | 46.1968591144798 | 0.926930509297557 |
| "ENSDART00000132912" | 15.6975480174097 | 24.1904962582364 | 0.926873582292424 |
| "ENSDART00000037709" | 289.184244237655 | 336.04687307392 | 0.926819725419973 |
| "ENSDART00000138667" | 30.8300379613296 | 43.7590685051022 | 0.926772036130081 |
| "ENSDART00000074425" | 8.97956101217881 | 14.5350700700478 | 0.926728504318319 |
| "ENSDART00000082333" | 65.3417980803657 | 84.6331720453777 | 0.926707616337737 |
| "ENSDART00000080770" | 569.663634612061 | 636.946460259373 | 0.926705101721169 |
| "ENSDART00000152704" | 8.70798401266822 | 15.8652972456439 | 0.926694258009439 |
| "ENSDART00000134592" | 485.633272573479 | 537.682827429521 | 0.926692790894383 |
| "ENSDART00000137154" | 19.2693978670602 | 106.303587201083 | 0.926675200739012 |
| "ENSDART00000142401" | 64.0329629954431 | 85.9798836899514 | 0.926631094169859 |
| "ENSDART00000156760" | 47.3867959827027 | 63.2822467569707 | 0.926609191810275 |
| "ENSDART00000132617" | 307.133100566859 | 386.730428988037 | 0.926595100777442 |
| "ENSDART00000139486" | 185.33903611472 | 220.663462869515 | 0.926591787982325 |
| "ENSDART00000081449" | 57.9132090961869 | 75.3721185969615 | 0.926568876695131 |
| "ENSDART00000077809" | 188.467070201745 | 235.711648422091 | 0.926550661213714 |
| "ENSDART00000149105" | 18.2680269979782 | 27.2392884648809 | 0.926465905235451 |
| "ENSDART00000026516" | 103.995608841443 | 142.149788603529 | 0.926382046394416 |
| "ENSDART00000154543" | 25.5695270809674 | 37.1985429729153 | 0.926362337200222 |
| "ENSDART00000152272" | 54.6769920927451 | 72.0006266683463 | 0.926256058038394 |
| "ENSDART00000122953" | 592.565841040982 | 676.281121889575 | 0.926250015851423 |
| "ENSDART00000124919" | 22.2110779893857 | 32.5644342234016 | 0.926220149721632 |
| "ENSDART00000139789" | 21.0618029667129 | 31.994763958028 | 0.926173179776888 |
| "ENSDART00000066648" | 20.648417037796 | 31.6462593487135 | 0.926125250992567 |
| "ENSDART00000132330" | 14.0794230082235 | 22.6756576410107 | 0.926051658934515 |
| "ENSDART00000111703" | 22.2110779893857 | 30.7113629373177 | 0.925935940874468 |
| "ENSDART00000066961" | 357.690198982952 | 415.03513313562 | 0.925886556459699 |
| "ENSDART00000081475" | 28.1852549682533 | 41.5555830631859 | 0.925841247852975 |
| "ENSDART00000099366" | 147.542778980898 | 180.571592537835 | 0.925748601776848 |
| "ENSDART00000132382" | 22.3631020696711 | 32.8923917006345 | 0.925644192310443 |
| "ENSDART00000113192" | 13.3092370449845 | 22.3933312265054 | 0.925597293090624 |
| "ENSDART00000062982" | 1113.17025872777 | 1208.00964501554 | 0.925550263981548 |
| "ENSDART00000138239" | 52.3893610697033 | 73.3002718391349 | 0.925501397161606 |
| "ENSDART00000129934" | 22.2703100379645 | 30.7173351469397 | 0.92541778615399 |
| "ENSDART00000054781" | 43.6372499873158 | 59.2534044628322 | 0.925402445625048 |
| "ENSDART00000110942" | 19.9830639655656 | 30.7278474062952 | 0.925381967182579 |
| "ENSDART00000022060" | 158.957875169709 | 206.097162266167 | 0.925372978021378 |
| "ENSDART00000064573" | 92.6735533181972 | 123.460753077372 | 0.925325883797475 |
| "ENSDART00000060764" | 937.993704403538 | 1028.84251724845 | 0.925287783789809 |
| "ENSDART00000156240" | 10.4377410084722 | 17.4123903464649 | 0.925256364237532 |
| "ENSDART00000109577" | 9.60832299665445 | 15.4998307807225 | 0.92521774519813 |
| "ENSDART00000005105" | 102.566516965871 | 134.816412955812 | 0.925199464420142 |
| "ENSDART00000128806" | 154.929920064272 | 194.501152582439 | 0.925157890048591 |
| "ENSDART00000047680" | 22.4826549888963 | 33.8141486006282 | 0.925126505379205 |
| "ENSDART00000113951" | 21.5562920037467 | 31.678750900039 | 0.925087554859201 |
| "ENSDART00000123546" | 79.1643641172952 | 113.560753032435 | 0.925067781130968 |
| "ENSDART00000108972" | 1459.04560679914 | 1969.97647232293 | 0.92506095212697 |
| "ENSDART00000139596" | 32.8426759295909 | 49.3545930077153 | 0.924959371568109 |
| "ENSDART00000130639" | 10.0545320223438 | 14.8857245447794 | 0.924907548845236 |
| "ENSDART00000135691" | 52.9657231161399 | 71.7235547579343 | 0.924860692851756 |
| "ENSDART00000112384" | 227.391017204553 | 268.854586189096 | 0.924829914550913 |
| "ENSDART00000007777" | 526.239096996976 | 613.505705658406 | 0.924777534083514 |
| "ENSDART00000028236" | 160.952377054484 | 193.802000000731 | 0.924743192053325 |
| "ENSDART00000132065" | 23.647001975498 | 34.1404335990735 | 0.924631598845781 |
| "ENSDART00000099446" | 143.503937868087 | 182.065646955249 | 0.924603533309732 |
| "ENSDART00000146115" | 47.6704138415152 | 71.2821155443769 | 0.924603190152283 |
| "ENSDART00000121648" | 37.1798879443611 | 54.3489137180572 | 0.924594268874686 |
| "ENSDART00000134393" | 27.1178199400528 | 41.2488871723943 | 0.924594125571121 |
| "ENSDART00000155763" | 31.9454010651622 | 43.7502287245344 | 0.924512316022668 |
| "ENSDART00000152585" | 19.0118370243416 | 25.7507288704595 | 0.92450604636917 |
| "ENSDART00000015777" | 1716.40712402506 | 1880.30684772169 | 0.9245036527694 |
| "ENSDART00000155100" | 67.3808119855027 | 87.5200465567224 | 0.924450929792903 |
| "ENSDART00000035305" | 16.2478530477525 | 25.0881240008429 | 0.924442096369354 |
| "ENSDART00000125411" | 11.4795039712218 | 18.5827870174801 | 0.924418851593343 |
| "ENSDART00000032331" | 119.586597304213 | 154.606387926467 | 0.924379502413218 |
| "ENSDART00000114658" | 76.3603729976815 | 100.239554328375 | 0.924374354592789 |
| "ENSDART00000109397" | 28.7020000154682 | 39.4311750085818 | 0.924364136346799 |
| "ENSDART00000130412" | 210.51855511395 | 247.486633119215 | 0.92430767813029 |
| "ENSDART00000113297" | 298.895941838194 | 370.516630384248 | 0.924229552942734 |
| "ENSDART00000098969" | 22.7734238790304 | 40.0596158480852 | 0.924222615462772 |
| "ENSDART00000127209" | 222.462371179417 | 268.986942578715 | 0.924181420714536 |
| "ENSDART00000146747" | 107.118401164775 | 138.454224216584 | 0.924176094881529 |
| "ENSDART00000112789" | 189.400199267573 | 223.688600054233 | 0.924118814399999 |
| "ENSDART00000058036" | 170.22506720493 | 205.98791433481 | 0.924107636290158 |
| "ENSDART00000085087" | 92.19522506716 | 114.147378651077 | 0.923978668519689 |
| "ENSDART00000142001" | 71.5385351610442 | 92.402080622898 | 0.923880612673842 |
| "ENSDART00000110735" | 11.8846170318189 | 17.9713080335837 | 0.923853012678351 |
| "ENSDART00000123220" | 24.5318840449124 | 34.7655294810015 | 0.923834996041222 |
| "ENSDART00000114678" | 280.56154928966 | 322.168913050497 | 0.923785470820151 |
| "ENSDART00000020961" | 354.263123137473 | 408.136914995902 | 0.923781304040732 |
| "ENSDART00000017171" | 8.946001029051 | 14.9507076474304 | 0.923773636962396 |
| "ENSDART00000135330" | 112.230018114271 | 138.593444914385 | 0.92377357509717 |
| "ENSDART00000128383" | 242.436693766814 | 284.663677437959 | 0.923773219003927 |
| "ENSDART00000127571" | 113.848044078665 | 140.230608299672 | 0.923772792018181 |
| "ENSDART00000133062" | 18.2865150171771 | 24.4728226727417 | 0.923772551376182 |
| "ENSDART00000140411" | 157.517765227041 | 188.688702731072 | 0.923772463889432 |
| "ENSDART00000098285" | 343.795088612076 | 398.218991819761 | 0.923772399963972 |
| "ENSDART00000013211" | 191.277794387106 | 231.762597969624 | 0.923772186988429 |
| "ENSDART00000059028" | 251.196490420233 | 285.358773858901 | 0.92377185265694 |
| "ENSDART00000151311" | 52.0736570004734 | 65.0982864419956 | 0.923771820600651 |
| "ENSDART00000024283" | 33.5714139920252 | 45.6379414273609 | 0.923771728285781 |
| "ENSDART00000087639" | 15.6307799868665 | 23.5885747604365 | 0.923771675056318 |
| "ENSDART00000062552" | 519.029838920397 | 572.037019063974 | 0.923771588874624 |
| "ENSDART00000113722" | 1530.29590469366 | 1684.447197839 | 0.923771509903784 |
| "ENSDART00000104731" | 45.5861510296608 | 56.4802487555423 | 0.923771456340886 |
| "ENSDART00000147267" | 12.0486819714063 | 19.2289074181192 | 0.923771292828547 |
| "ENSDART00000124957" | 90.1825540839681 | 112.645439226355 | 0.923771292082078 |
| "ENSDART00000126403" | 16.5978539864654 | 25.1949158220168 | 0.923771132095942 |
| "ENSDART00000144227" | 38.3401150042682 | 50.8567695001683 | 0.92377073505175 |
| "ENSDART00000112802" | 14.0349109878613 | 20.1351346223944 | 0.923769451126837 |
| "ENSDART00000127266" | 33.184403999984 | 45.3264684191055 | 0.923769023852855 |
| "ENSDART00000015190" | 47.7734210241009 | 59.9088420306686 | 0.923768395103753 |
| "ENSDART00000157018" | 156.967845147341 | 182.647024572136 | 0.923767936515593 |
| "ENSDART00000112568" | 9.91687603456274 | 17.6576851599111 | 0.923767646513949 |
| "ENSDART00000134913" | 178.070946425792 | 222.96483442617 | 0.923766838910474 |
| "ENSDART00000101282" | 383.272151873864 | 439.35558909682 | 0.923766585717317 |
| "ENSDART00000129984" | 30.4574290767232 | 41.6126811585281 | 0.923765543207105 |
| "ENSDART00000128412" | 58.1368580200654 | 76.7967157878581 | 0.923765277041862 |
| "ENSDART00000004782" | 539.736751120417 | 596.962280349765 | 0.923764843973896 |
| "ENSDART00000136701" | 11.5534560480172 | 17.3892159623367 | 0.923763913381147 |
| "ENSDART00000133603" | 27.7092539504184 | 40.975640857356 | 0.923763003240068 |
| "ENSDART00000150104" | 151.104948219379 | 176.120900137792 | 0.923762674351198 |
| "ENSDART00000083906" | 11.0551990200011 | 16.430427461284 | 0.923761844904849 |
| "ENSDART00000036371" | 125.773757075576 | 152.080199511411 | 0.92376173260568 |
| "ENSDART00000131861" | 352.680834800342 | 412.015955155848 | 0.923761358310422 |
| "ENSDART00000149013" | 20.8196660236356 | 27.9150328458996 | 0.923760934028803 |
| "ENSDART00000148311" | 46.5834019720482 | 59.2538818494617 | 0.923760382674383 |
| "ENSDART00000124966" | 213.459113399277 | 245.951250621077 | 0.923760342364424 |
| "ENSDART00000005090" | 265.676020086937 | 307.444754163784 | 0.923759962894118 |
| "ENSDART00000055360" | 728.077370262535 | 788.896384069546 | 0.923759647613069 |
| "ENSDART00000141358" | 443.837178420312 | 518.665936390958 | 0.923759615625861 |
| "ENSDART00000044837" | 44.2844999909903 | 58.0139620260619 | 0.923759554185892 |
| "ENSDART00000018475" | 80.032550861003 | 102.638881176805 | 0.92375937911021 |
| "ENSDART00000127236" | 377.138062897034 | 431.319343739186 | 0.923758748893644 |
| "ENSDART00000105862" | 45.2097411391416 | 60.4969063115376 | 0.923756771158614 |
| "ENSDART00000151621" | 58.6656439265823 | 76.0433261794157 | 0.923756350127474 |
| "ENSDART00000140634" | 54.5283840677296 | 67.2946045821317 | 0.923756349281554 |
| "ENSDART00000130053" | 301.308787553486 | 347.469669171597 | 0.923755615907156 |
| "ENSDART00000121884" | 28.5047270284654 | 38.4291456291177 | 0.923753985212893 |
| "ENSDART00000103513" | 538.11348339233 | 585.992798707853 | 0.923753575288058 |
| "ENSDART00000146825" | 34.0176230177146 | 45.58800738263 | 0.923753206724012 |
| "ENSDART00000002684" | 52.5832510163668 | 67.2845697094057 | 0.923752671588607 |
| "ENSDART00000113209" | 44.4029310732173 | 59.9697624702156 | 0.923749359799313 |
| "ENSDART00000007981" | 29.6992509578556 | 40.0426539924782 | 0.923747360385808 |
| "ENSDART00000149417" | 44.7423649253388 | 59.8825630078643 | 0.923745325568323 |
| "ENSDART00000081396" | 36.2309170133181 | 45.8743964602394 | 0.923744460981624 |
| "ENSDART00000122249" | 29.0071369981066 | 39.7873243063059 | 0.923743350496422 |
| "ENSDART00000112170" | 159.679429185891 | 185.187544339583 | 0.923741474940096 |
| "ENSDART00000053756" | 19.0118370243416 | 25.4569354233397 | 0.923737843672673 |
| "ENSDART00000102993" | 67.8194850292276 | 82.782250624711 | 0.923735913946782 |
| "ENSDART00000041304" | 402.37376735463 | 443.967418811935 | 0.923735836866498 |
| "ENSDART00000087120" | 1200.68122264393 | 1263.04253040565 | 0.923735636858233 |
| "ENSDART00000114825" | 82.9482399970958 | 103.665997738084 | 0.923735098493611 |
| "ENSDART00000133593" | 111.400534072592 | 133.472328563285 | 0.923731658928444 |
| "ENSDART00000061477" | 85.5779180113125 | 104.251675085806 | 0.923730666964369 |
| "ENSDART00000000280" | 8.26177498697881 | 14.0256057898614 | 0.923729716415823 |
| "ENSDART00000122041" | 41.8961890185651 | 53.6234756112068 | 0.923729631223303 |
| "ENSDART00000133067" | 15.7797399475943 | 23.8443818332383 | 0.92372707884965 |
| "ENSDART00000128012" | 145.878701193569 | 176.800229795286 | 0.923724270635562 |
| "ENSDART00000141918" | 12.4500270410212 | 20.5161307830344 | 0.923722500378507 |
| "ENSDART00000044505" | 68.7941280257216 | 85.2647175235572 | 0.92372059868145 |
| "ENSDART00000102014" | 155.67302621921 | 182.620742298163 | 0.923719453219674 |
| "ENSDART00000091818" | 146.349024952793 | 175.330544854158 | 0.92371796498841 |
| "ENSDART00000130643" | 14.0386789788436 | 22.6761350276402 | 0.923716885208015 |
| "ENSDART00000112414" | 551.883231525647 | 608.760090644953 | 0.923716223428112 |
| "ENSDART00000121826" | 13.1979569940791 | 18.9410861806214 | 0.923710675152081 |
| "ENSDART00000145484" | 86.451881058423 | 103.570195562895 | 0.923706571928727 |
| "ENSDART00000115747" | 11.9034569867301 | 18.2553069268767 | 0.923702106604583 |
| "ENSDART00000004514" | 97.4330949866982 | 118.527593125476 | 0.923700086965386 |
| "ENSDART00000102703" | 29.0889769925788 | 37.5171832829509 | 0.923699590028462 |
| "ENSDART00000109541" | 11.8883850228012 | 15.8146487465533 | 0.923698635471994 |
| "ENSDART00000062676" | 534.473946021783 | 588.740152778968 | 0.923694770109401 |
| "ENSDART00000126435" | 36.6740949343805 | 51.8884261111809 | 0.923693170185659 |
| "ENSDART00000135431" | 37.2945840505364 | 49.2915227027512 | 0.923692695935332 |
| "ENSDART00000018868" | 215.798290899866 | 255.638207084994 | 0.923692672175769 |
| "ENSDART00000048640" | 15.4629470562969 | 22.9942979510463 | 0.923688849864571 |
| "ENSDART00000136146" | 21.0802909859118 | 28.5480137351365 | 0.923687174875025 |
| "ENSDART00000108597" | 43.3995849066454 | 62.0807906945184 | 0.923685753794667 |
| "ENSDART00000132280" | 102.43231004829 | 124.910848910846 | 0.923684820280692 |
| "ENSDART00000133822" | 69.9019221326591 | 87.1598378470632 | 0.923684765328156 |
| "ENSDART00000081389" | 25.7633840127003 | 35.1051943097481 | 0.923684126464848 |
| "ENSDART00000125632" | 151.117341014393 | 180.634359849201 | 0.923683991345477 |
| "ENSDART00000039372" | 39.5308709426763 | 54.940800343131 | 0.923682179212472 |
| "ENSDART00000067152" | 476.5584932516 | 525.233158340633 | 0.923681828109961 |
| "ENSDART00000156705" | 377.664891035406 | 431.605805245 | 0.923678975575857 |
| "ENSDART00000130930" | 17.6132410123393 | 23.2653944006674 | 0.923678129803288 |
| "ENSDART00000130100" | 61.1912589510763 | 78.7350110639081 | 0.923671491955299 |
| "ENSDART00000156120" | 28.006118064737 | 37.2195674916263 | 0.923671219076566 |
| "ENSDART00000133417" | 13.3835410574923 | 19.8826725071679 | 0.923670260734028 |
| "ENSDART00000132241" | 30.9937509652046 | 47.3987925635616 | 0.923668730869209 |
| "ENSDART00000034595" | 14.3366319152298 | 24.2533294954702 | 0.923666930714235 |
| "ENSDART00000099885" | 22.9884479988768 | 30.4323814803877 | 0.923666461375604 |
| "ENSDART00000023421" | 1674.14174200761 | 1871.178493573 | 0.923666283339039 |
| "ENSDART00000154512" | 81.5571469521274 | 103.752420071377 | 0.923664690439344 |
| "ENSDART00000113889" | 69.3070720670236 | 96.9932514875978 | 0.923664461890624 |
| "ENSDART00000148535" | 212.981170098883 | 243.23423823587 | 0.923664443802386 |
| "ENSDART00000145285" | 157.366060067537 | 190.833243205827 | 0.923663134989096 |
| "ENSDART00000133562" | 35.3155060654029 | 45.305921287024 | 0.923659097326624 |
| "ENSDART00000098504" | 62.7233910241651 | 76.9302013437675 | 0.923656974808848 |
| "ENSDART00000106389" | 95.6279781712494 | 121.814101383025 | 0.923656641145381 |
| "ENSDART00000077089" | 682.222673337991 | 768.083262989396 | 0.923656085089169 |
| "ENSDART00000155769" | 82.8071679540448 | 106.180719120525 | 0.923654745971838 |
| "ENSDART00000155002" | 86.6042570744207 | 105.867333314583 | 0.923652496874913 |
| "ENSDART00000115026" | 161.208497139423 | 186.948158408054 | 0.923650724870128 |
| "ENSDART00000141317" | 14.7081849926992 | 19.8325013947068 | 0.923650469705502 |
| "ENSDART00000100000" | 139.611494121226 | 166.588750239755 | 0.92365008986021 |
| "ENSDART00000124176" | 66.4661379239428 | 89.0511325751954 | 0.92364631596647 |
| "ENSDART00000059170" | 20.0162720129811 | 27.3037941809024 | 0.923644715156984 |
| "ENSDART00000101620" | 28.1037669094934 | 41.2548593820163 | 0.923641134126266 |
| "ENSDART00000124963" | 76.7983091550511 | 96.4438253607083 | 0.923640911526794 |
| "ENSDART00000043126" | 15.4448109728103 | 22.0388544075689 | 0.92364076259538 |
| "ENSDART00000097320" | 13.1346050188057 | 23.6205889251325 | 0.923637870470162 |
| "ENSDART00000054293" | 12.0154739239908 | 21.1092125002664 | 0.923635142913607 |
| "ENSDART00000032502" | 32.6748429990213 | 42.8263219591236 | 0.923634006591827 |
| "ENSDART00000123292" | 55.0273449671703 | 77.3432116691035 | 0.923633795093784 |
| "ENSDART00000112288" | 298.703191257873 | 336.104277414029 | 0.923632424174463 |
| "ENSDART00000145069" | 529.940314480502 | 583.922084367487 | 0.923630815008411 |
| "ENSDART00000141769" | 26.4931778822717 | 39.9501967748646 | 0.923630456851653 |
| "ENSDART00000023959" | 47.178603973396 | 61.7662130475868 | 0.923626523786818 |
| "ENSDART00000132103" | 23.3196089826785 | 30.7185302390978 | 0.923626073187724 |
| "ENSDART00000155646" | 16.4043820053754 | 23.2484325450604 | 0.923625695295759 |
| "ENSDART00000081840" | 79.0572039930844 | 98.7457402298601 | 0.923625657523982 |
| "ENSDART00000021609" | 1813.69064162276 | 2066.15583137535 | 0.923622811670731 |
| "ENSDART00000074552" | 15.8276680232264 | 20.4802942741335 | 0.923619767868789 |
| "ENSDART00000144860" | 14.9982500114086 | 22.9837856916908 | 0.923618888480083 |
| "ENSDART00000129498" | 197.577807026877 | 228.746471707337 | 0.923617567467211 |
| "ENSDART00000138831" | 23.2302330062419 | 31.6689563462122 | 0.923614634867801 |
| "ENSDART00000151440" | 23.8212820659645 | 33.2253666142305 | 0.923614609690607 |
| "ENSDART00000114131" | 23.7284900342579 | 34.1141545762691 | 0.923613721528385 |
| "ENSDART00000041036" | 325.266971191532 | 367.814561840966 | 0.923613472687054 |
| "ENSDART00000097669" | 12.9821959878774 | 18.6207733917981 | 0.923608242109591 |
| "ENSDART00000132096" | 101.435795992258 | 126.34500033667 | 0.923604522381512 |
| "ENSDART00000156946" | 93.9657260923439 | 115.210331721257 | 0.923604307477732 |
| "ENSDART00000047419" | 43.5184339544459 | 58.9674927718524 | 0.923603068259135 |
| "ENSDART00000138726" | 60.6998010186694 | 75.4191850707466 | 0.923599964130867 |
| "ENSDART00000112131" | 211.041278811213 | 252.891817540645 | 0.923598658336807 |
| "ENSDART00000104031" | 719.00558477329 | 775.699195617011 | 0.92359762777869 |
| "ENSDART00000059918" | 186.320126271111 | 215.585047335498 | 0.923593326352266 |
| "ENSDART00000045245" | 830.660142802133 | 907.176943976922 | 0.923580303063068 |
| "ENSDART00000022233" | 121.827389073691 | 152.164294335086 | 0.923579890551259 |
| "ENSDART00000131620" | 17.1779840238843 | 25.1261103751609 | 0.923577967252931 |
| "ENSDART00000148254" | 202.118321222973 | 235.254676261646 | 0.92357141644865 |
| "ENSDART00000137573" | 118.929869036015 | 149.088268332378 | 0.923569502631501 |
| "ENSDART00000138026" | 57.5269690054316 | 73.8403181241288 | 0.923569287753126 |
| "ENSDART00000074116" | 37.3051841520585 | 51.7567874270912 | 0.92356285754688 |
| "ENSDART00000126780" | 401.973242730576 | 471.284971496111 | 0.92356210654808 |
| "ENSDART00000099559" | 28.7389760538659 | 37.5508699264346 | 0.923560054707951 |
| "ENSDART00000142155" | 82.5499590470385 | 106.7948221053 | 0.923552858787824 |
| "ENSDART00000149971" | 20.1796000662132 | 28.4813581536978 | 0.923552504342065 |
| "ENSDART00000153478" | 7.94568596710604 | 16.2001849569268 | 0.923550294865725 |
| "ENSDART00000005345" | 309.591496580305 | 365.048441583721 | 0.923550210921342 |
| "ENSDART00000148889" | 151.938805078673 | 184.210598890765 | 0.923549753948772 |
| "ENSDART00000129924" | 1237.44869265024 | 1320.2776041807 | 0.923548354870009 |
| "ENSDART00000101550" | 17.2820800285376 | 22.9302696216543 | 0.923546946242274 |
| "ENSDART00000131235" | 41.9407010389273 | 53.0174914503028 | 0.923541739653083 |
| "ENSDART00000136301" | 11.8065450283289 | 17.0385614876051 | 0.923540068439528 |
| "ENSDART00000081317" | 52.668122115466 | 69.4438562484828 | 0.923539340052827 |
| "ENSDART00000042874" | 43.4584650195118 | 55.4856237455888 | 0.92353875839847 |
| "ENSDART00000131330" | 44.4557159618992 | 60.623290491534 | 0.923537930253974 |
| "ENSDART00000140709" | 16.534502011192 | 22.3684843635895 | 0.923536877682016 |
| "ENSDART00000040383" | 75.6165629713182 | 92.7393552673282 | 0.923536306111141 |
| "ENSDART00000146795" | 81.2516910487072 | 101.717601663441 | 0.923534734694412 |
| "ENSDART00000137766" | 210.885955249794 | 242.999630074811 | 0.92353351363407 |
| "ENSDART00000153877" | 12.2678260179473 | 18.5694071871788 | 0.923531765288176 |
| "ENSDART00000127814" | 121.641738980416 | 149.792375675751 | 0.923526792392121 |
| "ENSDART00000044085" | 263.966528318238 | 353.776869400232 | 0.923525381362533 |
| "ENSDART00000151418" | 287.709116024735 | 330.776981790091 | 0.923517734199909 |
| "ENSDART00000118422" | 75.7542189590993 | 98.4949473422525 | 0.923514745692413 |
| "ENSDART00000033608" | 22.18470205251 | 31.0233133322026 | 0.923512417606452 |
| "ENSDART00000006787" | 240.16949304553 | 274.378677992807 | 0.923508790015977 |
| "ENSDART00000133304" | 466.048592859824 | 525.24642970377 | 0.923499844919082 |
| "ENSDART00000023449" | 426.080406071997 | 499.461167883732 | 0.923496504439484 |
| "ENSDART00000151186" | 33.7166059617708 | 44.7099719988461 | 0.923495542437038 |
| "ENSDART00000152074" | 18.4501950061215 | 26.6691408128778 | 0.923490593479012 |
| "ENSDART00000147100" | 11.9404330251278 | 17.3462121516557 | 0.923489330265611 |
| "ENSDART00000045036" | 570.693103824589 | 635.554838508769 | 0.923484853669808 |
| "ENSDART00000090413" | 177.193046055984 | 207.023867425488 | 0.923481661617015 |
| "ENSDART00000062682" | 388.026568352809 | 428.513525338707 | 0.923479159202101 |
| "ENSDART00000135397" | 17.2968000567542 | 24.5296837003535 | 0.923476466814229 |
| "ENSDART00000148545" | 17.1968239787954 | 23.5885747604365 | 0.923475803184209 |
| "ENSDART00000078266" | 1656.11399544266 | 1912.59803210278 | 0.923475171182016 |
| "ENSDART00000059930" | 24.1633950870006 | 33.4795012082445 | 0.923472826134561 |
| "ENSDART00000080042" | 34.2672959427565 | 47.1974563340553 | 0.92346832040901 |
| "ENSDART00000132703" | 114.945689064585 | 138.351732126245 | 0.923467611796325 |
| "ENSDART00000055745" | 191.662510160876 | 220.09659424922 | 0.923455516449102 |
| "ENSDART00000090306" | 543.556947667707 | 625.655480489987 | 0.923449018846411 |
| "ENSDART00000019743" | 1599.79197483561 | 1675.20567019715 | 0.923447996456339 |
| "ENSDART00000115330" | 128.103638915639 | 175.629108916404 | 0.923444926024557 |
| "ENSDART00000111125" | 34.8840170679301 | 46.4765582769384 | 0.92343308780678 |
| "ENSDART00000099665" | 793.164737649601 | 853.345929361194 | 0.923426978418064 |
| "ENSDART00000155221" | 11.1072470223278 | 15.5284999878431 | 0.923426909974246 |
| "ENSDART00000106086" | 22.099061052125 | 32.2964424124566 | 0.923425557351085 |
| "ENSDART00000098347" | 12.1267539748963 | 19.5664223814487 | 0.923416860860074 |
| "ENSDART00000062671" | 1988.33999767292 | 2190.17569694237 | 0.923414260332704 |
| "ENSDART00000149752" | 39.2363010466293 | 52.3759110993973 | 0.923410812842136 |
| "ENSDART00000136709" | 109.169137008432 | 135.742946973269 | 0.923397541329343 |
| "ENSDART00000067446" | 2494.67561851068 | 2693.70901364842 | 0.92339640465279 |
| "ENSDART00000137218" | 16.8841510141926 | 21.0922506446594 | 0.923396322887581 |
| "ENSDART00000154296" | 19.4433260218144 | 28.5907772269183 | 0.923387667831356 |
| "ENSDART00000128271" | 158.390203957166 | 193.343184219635 | 0.923386477744097 |
| "ENSDART00000112263" | 39.9431350345949 | 52.1817421716712 | 0.923385738020505 |
| "ENSDART00000136617" | 10.3596690049822 | 14.8625501606512 | 0.923380028746331 |
| "ENSDART00000143541" | 11.8585930306556 | 17.3423898074508 | 0.923375786393899 |
| "ENSDART00000134066" | 12.6099390389834 | 17.382526047186 | 0.923374813565105 |
| "ENSDART00000106035" | 23.2750969623164 | 31.9171187306043 | 0.92337218285233 |
| "ENSDART00000091982" | 545.563358352061 | 629.893435553775 | 0.923372145740776 |
| "ENSDART00000151822" | 389.102628185041 | 426.566561423952 | 0.923367421811605 |
| "ENSDART00000122709" | 106.371593048715 | 129.042897749684 | 0.92336605301907 |
| "ENSDART00000051065" | 290.013376343622 | 327.759663686815 | 0.923365360833648 |
| "ENSDART00000135686" | 371.516198604496 | 430.477754102666 | 0.92336413434345 |
| "ENSDART00000080777" | 306.131076370627 | 342.713232402853 | 0.923362433671137 |
| "ENSDART00000005305" | 47.5470929313078 | 62.9227525016712 | 0.923360562597356 |
| "ENSDART00000153063" | 20.9049220733777 | 28.8198278902863 | 0.923339676775296 |
| "ENSDART00000154170" | 17.6505689864493 | 25.4633850195912 | 0.923332557486153 |
| "ENSDART00000022135" | 224.279561869098 | 273.143535286871 | 0.923329584239223 |
| "ENSDART00000147648" | 30.684845991584 | 44.6330412257822 | 0.923321147895378 |
| "ENSDART00000100207" | 68.2167101721479 | 86.2323458051778 | 0.923316862364819 |
| "ENSDART00000111203" | 123.547249839397 | 153.561832122951 | 0.923304194402837 |
| "ENSDART00000026276" | 29.6434349645467 | 43.4019611829502 | 0.923293256607812 |
| "ENSDART00000056913" | 12.9301479855507 | 21.4452953037074 | 0.923292062954554 |
| "ENSDART00000088358" | 66.4616660615359 | 87.6160890508105 | 0.923278129550723 |
| "ENSDART00000079563" | 11.7993609820768 | 17.7126333898361 | 0.923277153831666 |
| "ENSDART00000145963" | 22.020989048635 | 29.7757488203931 | 0.923276452876914 |
| "ENSDART00000114785" | 11.4384080061295 | 17.011564759272 | 0.923274654673578 |
| "ENSDART00000153873" | 18.9337650208516 | 26.675353341399 | 0.923267596328052 |
| "ENSDART00000110020" | 88.1662141545857 | 109.096437232023 | 0.923266561544277 |
| "ENSDART00000132926" | 77.3154061379784 | 106.596600267976 | 0.923264750308177 |
| "ENSDART00000085402" | 73.8744461954304 | 94.0674325775072 | 0.923258846140761 |
| "ENSDART00000014573" | 117.460319036914 | 150.936559249829 | 0.923258770214993 |
| "ENSDART00000104812" | 148.406526877129 | 180.514968577953 | 0.923253240740051 |
| "ENSDART00000012855" | 715.320831510957 | 770.00397091563 | 0.923246740545662 |
| "ENSDART00000103724" | 1287.35321573976 | 1402.64781613556 | 0.923240991101452 |
| "ENSDART00000131227" | 193.179570270036 | 230.3148263946 | 0.923240467863264 |
| "ENSDART00000058504" | 99.8790146459246 | 133.242563445557 | 0.923239358319792 |
| "ENSDART00000152205" | 44.8382210766029 | 56.7597075991017 | 0.923230361349135 |
| "ENSDART00000121434" | 65.0935659131038 | 83.0789116427766 | 0.923229718264441 |
| "ENSDART00000132484" | 37.3949120642075 | 54.592773120446 | 0.923218168478778 |
| "ENSDART00000064860" | 598.765824845216 | 649.069890142282 | 0.923217801609324 |
| "ENSDART00000127845" | 210.234519289564 | 255.634937806793 | 0.92321577547243 |
| "ENSDART00000013148" | 427.422510977135 | 500.054309024494 | 0.923214404868302 |
| "ENSDART00000145830" | 49.5257859657984 | 63.3104385774619 | 0.923207635674556 |
| "ENSDART00000061639" | 666.349419957921 | 739.456576825372 | 0.923193469482807 |
| "ENSDART00000022379" | 280.606380230804 | 315.18803874933 | 0.92319271275397 |
| "ENSDART00000122752" | 31.0790070149467 | 41.2433923494018 | 0.923192646476779 |
| "ENSDART00000028072" | 28.1558149118201 | 40.6276136346711 | 0.923190805906766 |
| "ENSDART00000052091" | 316.795915373179 | 360.992428168218 | 0.923189330269888 |
| "ENSDART00000140155" | 1239.56242853346 | 1487.11546514995 | 0.923185746908742 |
| "ENSDART00000136650" | 21.2918990504883 | 28.5171946625987 | 0.923184778166139 |
| "ENSDART00000110004" | 210.054561940486 | 243.840222396774 | 0.923172013945634 |
| "ENSDART00000139397" | 55.432491042698 | 72.0008637360766 | 0.923160789930834 |
| "ENSDART00000135599" | 35.8182679707565 | 50.5610632553617 | 0.923159634756432 |
| "ENSDART00000109396" | 385.274724280313 | 435.374702123859 | 0.923150856250701 |
| "ENSDART00000141693" | 108.871821913609 | 143.332735680983 | 0.923146057554165 |
| "ENSDART00000133831" | 11.5164800096195 | 15.8277882579554 | 0.923143329501572 |
| "ENSDART00000143172" | 44.1992109263176 | 59.7855657405172 | 0.923142697695631 |
| "ENSDART00000090922" | 97.4074559361778 | 118.842411091307 | 0.923140468588007 |
| "ENSDART00000076399" | 441.134738239918 | 488.586912316722 | 0.923131790513381 |
| "ENSDART00000066308" | 182.921955942356 | 220.711000227592 | 0.923124601402231 |
| "ENSDART00000133537" | 16.4341739975209 | 23.541030900022 | 0.923120757768479 |
| "ENSDART00000127068" | 54.8482410785846 | 71.0948768507007 | 0.92311728515302 |
| "ENSDART00000138235" | 17.1968239787954 | 24.5406733463385 | 0.923113102643834 |
| "ENSDART00000038990" | 621.333735883854 | 699.140195880057 | 0.923112265292851 |
| "ENSDART00000081643" | 211.298956228068 | 240.456542478847 | 0.923108649185026 |
| "ENSDART00000110158" | 408.996432748307 | 464.72921657905 | 0.923108037637033 |
| "ENSDART00000114613" | 143.227438025666 | 171.494199008958 | 0.923103505321561 |
| "ENSDART00000108659" | 37.2205989588104 | 51.8306103103101 | 0.923101337005235 |
| "ENSDART00000152942" | 331.082441568641 | 373.904201823416 | 0.923100371817063 |
| "ENSDART00000052222" | 500.253724694167 | 563.421137745276 | 0.923099653722693 |
| "ENSDART00000002778" | 294.055600496772 | 337.431877337578 | 0.923098110688249 |
| "ENSDART00000099872" | 620.289694188419 | 689.359337033962 | 0.923094918045347 |
| "ENSDART00000052183" | 2122.80185831515 | 2212.28573307395 | 0.923093237531053 |
| "ENSDART00000002866" | 14.4588640033697 | 20.7791051576163 | 0.923091540137792 |
| "ENSDART00000135394" | 34.5528560841284 | 45.8364100859213 | 0.923086902915811 |
| "ENSDART00000133574" | 350.638624715925 | 401.002593860539 | 0.923080103917189 |
| "ENSDART00000099735" | 92.5818501085583 | 110.45485622811 | 0.923071190539891 |
| "ENSDART00000064558" | 1040.08365279693 | 1126.92838182557 | 0.923069916462844 |
| "ENSDART00000141072" | 9.95420400867278 | 15.8433179536739 | 0.92306969541492 |
| "ENSDART00000113353" | 81.7567471723325 | 100.147337429661 | 0.923062946977542 |
| "ENSDART00000141068" | 319.163829058776 | 373.668932129189 | 0.923059253211588 |
| "ENSDART00000133758" | 14.7862569961891 | 21.0862784350374 | 0.923057035098065 |
| "ENSDART00000137253" | 8.56691196961721 | 15.5466569356083 | 0.923054528753932 |
| "ENSDART00000114741" | 257.918095827379 | 300.55842427095 | 0.923043555880928 |
| "ENSDART00000147867" | 283.431923489174 | 330.872072762089 | 0.92303658502941 |
| "ENSDART00000017180" | 119.03770001672 | 143.515615220296 | 0.923032438747354 |
| "ENSDART00000087036" | 23.249072961153 | 31.9154462518166 | 0.923021421209397 |
| "ENSDART00000138028" | 9.70111502836104 | 15.2246716679974 | 0.923020666511505 |
| "ENSDART00000034850" | 660.739310618833 | 726.414698610799 | 0.923013865397737 |
| "ENSDART00000134189" | 190.496506797566 | 224.932822859636 | 0.923010821011904 |
| "ENSDART00000043751" | 47.784021125623 | 63.260504532731 | 0.92300962391786 |
| "ENSDART00000023676" | 710.707687208406 | 774.087590297831 | 0.9230088541715 |
| "ENSDART00000113792" | 115.428841113741 | 136.182651033341 | 0.923008107348083 |
| "ENSDART00000126290" | 43.9012579899313 | 56.7570803470551 | 0.92300796908737 |
| "ENSDART00000062654" | 127.544676066334 | 157.019809059558 | 0.923007344199297 |
| "ENSDART00000156124" | 116.991117114688 | 145.041032022744 | 0.92300692069209 |
| "ENSDART00000153683" | 9.71995498327223 | 14.5814188383041 | 0.923002542832388 |
| "ENSDART00000156621" | 177.165581297041 | 207.637259207071 | 0.922989476308331 |
| "ENSDART00000122535" | 131.737366967852 | 157.569890217278 | 0.922985458970267 |
| "ENSDART00000131116" | 860.675249631588 | 987.966431733456 | 0.922983772101555 |
| "ENSDART00000128433" | 436.691619997658 | 488.971964638128 | 0.922982595652407 |
| "ENSDART00000150626" | 13.357517056329 | 20.7812550230334 | 0.922981526696863 |
| "ENSDART00000135010" | 18.0187060086487 | 25.1746090088344 | 0.922979848248132 |
| "ENSDART00000141170" | 229.014032041719 | 268.726226536715 | 0.922972749543656 |
| "ENSDART00000154554" | 121.967691215456 | 145.144890347105 | 0.922972062577969 |
| "ENSDART00000153857" | 9.29600196776391 | 15.2440237079207 | 0.922968029265329 |
| "ENSDART00000141130" | 117.360727909598 | 149.963676464116 | 0.922965269035897 |
| "ENSDART00000034935" | 138.089929134729 | 162.781073601676 | 0.922962765641776 |
| "ENSDART00000145665" | 39.5859500496299 | 51.1073220688768 | 0.922962750996333 |
| "ENSDART00000075617" | 226.643857152781 | 262.094330980323 | 0.92296028244569 |
| "ENSDART00000109026" | 38.6897640072688 | 49.3178017255555 | 0.922954111269728 |
| "ENSDART00000142972" | 23.6843299496081 | 35.6999485057678 | 0.922953536080683 |
| "ENSDART00000142740" | 20.0683200153078 | 28.5687979349483 | 0.922951853789638 |
| "ENSDART00000102651" | 420.860755795151 | 474.680516902739 | 0.922948140100344 |
| "ENSDART00000134788" | 29.929347041631 | 41.8985928495079 | 0.922946790238107 |
| "ENSDART00000017202" | 27.1510279874682 | 37.9125140471512 | 0.922934935387419 |
| "ENSDART00000153604" | 11.4904560084562 | 17.3746410398771 | 0.92293284214427 |
| "ENSDART00000147845" | 324.832836040075 | 369.441387359929 | 0.922931528511006 |
| "ENSDART00000156479" | 76.4218992645319 | 99.6505352742468 | 0.92292571373809 |
| "ENSDART00000106620" | 25.6962640464447 | 36.980244887802 | 0.922922256172793 |
| "ENSDART00000140814" | 45.642318958682 | 60.1890185797567 | 0.922917206168582 |
| "ENSDART00000151727" | 106.379447951462 | 135.351682123342 | 0.922915101142433 |
| "ENSDART00000078781" | 302.529720434682 | 343.514649760678 | 0.922913288227317 |
| "ENSDART00000137211" | 39.5750310273261 | 54.6145153446858 | 0.922911371749111 |
| "ENSDART00000123032" | 98.2961059965744 | 117.23367984541 | 0.922891897078422 |
| "ENSDART00000063135" | 647.100133471837 | 700.695101559982 | 0.922887226816751 |
| "ENSDART00000130804" | 70.3416509835211 | 89.6360922173882 | 0.922883885373715 |
| "ENSDART00000129353" | 140.754255954141 | 172.07802948486 | 0.922882688109832 |
| "ENSDART00000128733" | 49.2014570925364 | 64.7622036385545 | 0.92288106708302 |
| "ENSDART00000146484" | 40.7725860613433 | 54.6780662874483 | 0.922880718337042 |
| "ENSDART00000144252" | 32.8943719962052 | 45.6776002804666 | 0.92286787829652 |
| "ENSDART00000120252" | 550.609463211493 | 603.346415229019 | 0.922867080531325 |
| "ENSDART00000151254" | 14.2578560403152 | 22.3481775504072 | 0.922863773617703 |
| "ENSDART00000019078" | 32.2912820771805 | 42.8526009819279 | 0.922859786879218 |
| "ENSDART00000111504" | 14.689345037788 | 21.4395601618157 | 0.922858093615277 |
| "ENSDART00000122584" | 67.0047540306958 | 99.8091739378385 | 0.92284910938723 |
| "ENSDART00000037855" | 288.68943627984 | 326.507381481071 | 0.922845640986279 |
| "ENSDART00000114712" | 489.44256762781 | 548.052639403877 | 0.922844932832728 |
| "ENSDART00000051799" | 1218.54492196818 | 1340.20702066104 | 0.922841810231968 |
| "ENSDART00000124187" | 10.1250680438694 | 15.548329414396 | 0.922835628606639 |
| "ENSDART00000004769" | 156.469588119325 | 182.317394616115 | 0.922834528979338 |
| "ENSDART00000114304" | 1828.27333640569 | 1926.21105908728 | 0.92282805107379 |
| "ENSDART00000011166" | 17.2190799889765 | 22.9732734323353 | 0.922826885220352 |
| "ENSDART00000022045" | 232.893346107211 | 265.569513342605 | 0.922825872974229 |
| "ENSDART00000129564" | 11.0774550301822 | 16.4540792320417 | 0.922814362107243 |
| "ENSDART00000010918" | 522.994246174281 | 634.116212959079 | 0.922812506004137 |
| "ENSDART00000155075" | 21.6268280252722 | 31.3734904203048 | 0.922810191583244 |
| "ENSDART00000067048" | 136.758068138339 | 161.673747235625 | 0.922803287021865 |
| "ENSDART00000103261" | 75.7123861076517 | 98.7710677305744 | 0.922802162329893 |
| "ENSDART00000155652" | 19.1268850662292 | 27.9346252047222 | 0.922797566374201 |
| "ENSDART00000147473" | 167.776318787722 | 209.29705366399 | 0.922786203031056 |
| "ENSDART00000153130" | 36.6586380198086 | 45.5827528785367 | 0.922780258768032 |
| "ENSDART00000103406" | 83.9484890291795 | 112.878549301658 | 0.922775107836446 |
| "ENSDART00000132444" | 16.7615669903404 | 24.8249093073618 | 0.922773928196116 |
| "ENSDART00000128064" | 27.7794380362315 | 41.2524724488688 | 0.922768861582596 |
| "ENSDART00000146605" | 29.2074410897364 | 40.3082556191067 | 0.922757840207142 |
| "ENSDART00000140712" | 12.8373559538441 | 22.330020602642 | 0.92275471706657 |
| "ENSDART00000152240" | 207.223424982711 | 244.744293233294 | 0.922754303532602 |
| "ENSDART00000031322" | 26.9798120165593 | 40.0507760675173 | 0.922747106709595 |
| "ENSDART00000142260" | 31.8974729895301 | 43.8092396175634 | 0.922723732032093 |
| "ENSDART00000137358" | 37.4216729517261 | 52.8374200584068 | 0.922720000440423 |
| "ENSDART00000137641" | 38.7900920209399 | 48.9759870313917 | 0.922712141534123 |
| "ENSDART00000122746" | 110.883437089664 | 130.867777215276 | 0.922710765025553 |
| "ENSDART00000027443" | 230.313120485612 | 266.869398832293 | 0.92270576741523 |
| "ENSDART00000093306" | 16.0543810666625 | 26.3074966921612 | 0.922705126236642 |
| "ENSDART00000054691" | 2178.97778991916 | 2272.46872714928 | 0.922704394312235 |
| "ENSDART00000152115" | 11.1370390144734 | 17.7513374696828 | 0.922700439915391 |
| "ENSDART00000131510" | 91.7045040211084 | 112.541755295026 | 0.922697116331361 |
| "ENSDART00000049593" | 7.88233399183268 | 14.0365954358464 | 0.922695957741276 |
| "ENSDART00000138596" | 140.18492836489 | 182.259815882975 | 0.922691750372463 |
| "ENSDART00000005727" | 77.4870070595302 | 96.7524307980179 | 0.922689565594489 |
| "ENSDART00000083068" | 245.227940155701 | 280.399809019661 | 0.922689244942595 |
| "ENSDART00000131240" | 153.753532218368 | 182.545790248653 | 0.922686418153256 |
| "ENSDART00000132753" | 67.8006450743164 | 83.1030408001638 | 0.92267311585667 |
| "ENSDART00000077053" | 183.803938966865 | 220.71309066948 | 0.922671557744428 |
| "ENSDART00000155928" | 47.4648679861926 | 62.8993410498128 | 0.92267151810519 |
| "ENSDART00000139478" | 72.4539461089594 | 89.6356148307587 | 0.922671030544717 |
| "ENSDART00000073970" | 170.266449075873 | 216.843907738059 | 0.922670320923265 |
| "ENSDART00000109628" | 76.8552469853582 | 97.6362013236936 | 0.922669648979692 |
| "ENSDART00000152622" | 184.032110297426 | 220.916883009169 | 0.922667918189064 |
| "ENSDART00000098132" | 175.569679283105 | 210.286723912278 | 0.922662489816569 |
| "ENSDART00000154238" | 24.6103409990453 | 34.4244324923664 | 0.922648379196506 |
| "ENSDART00000098292" | 24.7661330703129 | 34.1497507662708 | 0.922648326478782 |
| "ENSDART00000151248" | 281.694311590624 | 321.229779582965 | 0.922645259973099 |
| "ENSDART00000075935" | 274.474468898109 | 324.824040469391 | 0.922634377287531 |
| "ENSDART00000145535" | 48.3648550344665 | 65.1052134248765 | 0.922633153781308 |
| "ENSDART00000108469" | 23.2343529329364 | 33.5053028444194 | 0.922630613756672 |
| "ENSDART00000020908" | 860.871902306372 | 952.991852157502 | 0.922629682663256 |
| "ENSDART00000134625" | 31.5919510562488 | 47.2031914759471 | 0.922627935665051 |
| "ENSDART00000151994" | 20.354617043035 | 28.8606818355501 | 0.922622653340916 |
| "ENSDART00000075486" | 160.692071012989 | 188.313741454752 | 0.922617767213181 |
| "ENSDART00000012064" | 15.4780190202258 | 23.197066340441 | 0.922616197778736 |
| "ENSDART00000133326" | 26.4694811143106 | 38.8110965630167 | 0.922615819428398 |
| "ENSDART00000007249" | 37.0414950702247 | 48.6434895044252 | 0.922614326243753 |
| "ENSDART00000145572" | 4231.15354141707 | 4764.87221608345 | 0.92261136930671 |
| "ENSDART00000087450" | 50.1654999875084 | 67.251837839181 | 0.922611035509331 |
| "ENSDART00000122138" | 1041.21326520583 | 1101.60069815317 | 0.922609758694716 |
| "ENSDART00000065212" | 163.701959923638 | 201.326568538064 | 0.92260167472635 |
| "ENSDART00000102183" | 62.8421740421044 | 82.2608419252807 | 0.922590861588488 |
| "ENSDART00000010370" | 14.0458630250957 | 19.8671428114494 | 0.922584272305995 |
| "ENSDART00000080348" | 52.6572030931622 | 68.2564977218605 | 0.922571402380439 |
| "ENSDART00000082517" | 42.49100606927 | 57.0785882280366 | 0.92255944391791 |
| "ENSDART00000030687" | 35.7213560123553 | 48.4027347407125 | 0.922555715762039 |
| "ENSDART00000132958" | 459.719474569988 | 538.537862822209 | 0.92254681031216 |
| "ENSDART00000127513" | 296.191944325883 | 340.139335487858 | 0.922540187040848 |
| "ENSDART00000017176" | 781.378224700104 | 854.474270492449 | 0.922526375031173 |
| "ENSDART00000061376" | 124.60532317721 | 149.199537528587 | 0.922522938879802 |
| "ENSDART00000148131" | 40.8958739566201 | 55.5515648726677 | 0.922520222219252 |
| "ENSDART00000113179" | 569.916690577443 | 613.993430965522 | 0.922516825736614 |
| "ENSDART00000127900" | 466.060248768483 | 526.428401874564 | 0.922513673603137 |
| "ENSDART00000112579" | 1691.85269602036 | 1863.57144264086 | 0.922507587016764 |
| "ENSDART00000022729" | 14.9759940012275 | 23.0344341907815 | 0.922499497607824 |
| "ENSDART00000103236" | 12.2825460461639 | 19.8810000283803 | 0.922494164975822 |
| "ENSDART00000132672" | 23.3120730007141 | 31.6794686055677 | 0.922488655056213 |
| "ENSDART00000143810" | 99.5974050995325 | 123.035317694994 | 0.922482847091815 |
| "ENSDART00000056306" | 68.7303910998052 | 84.0964707179591 | 0.922468405776184 |
| "ENSDART00000123666" | 154.739831123521 | 182.195550485852 | 0.922463612745449 |
| "ENSDART00000021657" | 67.8424449108334 | 85.9538417348773 | 0.922462984212548 |
| "ENSDART00000081457" | 22.0062690204184 | 29.1456355021021 | 0.922462490581113 |
| "ENSDART00000123062" | 346.758587036361 | 424.543519544566 | 0.922461448240554 |
| "ENSDART00000130977" | 97.5036970380849 | 122.843538951584 | 0.922457323561129 |
| "ENSDART00000144146" | 11.5606400942693 | 20.147796747167 | 0.922453515972092 |
| "ENSDART00000108841" | 35.6990669872436 | 48.4163516387441 | 0.922453489806922 |
| "ENSDART00000052419" | 22.2555900097479 | 29.8149302868693 | 0.922451916723596 |
| "ENSDART00000154052" | 12.5431710084401 | 17.6856366615031 | 0.922450813521888 |
| "ENSDART00000122782" | 54.4801040563852 | 71.6717144178544 | 0.922442182635417 |
| "ENSDART00000153667" | 2804.32791061029 | 3243.85538916527 | 0.922441778949975 |
| "ENSDART00000114987" | 45.8727999931004 | 63.3326549371622 | 0.922439695435529 |
| "ENSDART00000075601" | 46.0731040847301 | 58.5943816185213 | 0.922437823767265 |
| "ENSDART00000049551" | 327.877072364482 | 363.460326646942 | 0.922437308044394 |
| "ENSDART00000144804" | 84.9971171173995 | 104.829227107319 | 0.922433735441556 |
| "ENSDART00000074710" | 48.1977589902522 | 64.7399872788542 | 0.922423823498867 |
| "ENSDART00000113586" | 41.8886530366006 | 53.9729349937802 | 0.922418579615802 |
| "ENSDART00000015801" | 188.477285352624 | 220.50721439024 | 0.922407149081988 |
| "ENSDART00000124159" | 28.8102159468161 | 42.6304805526098 | 0.922401517361998 |
| "ENSDART00000128778" | 25.5438550155164 | 32.8646772667728 | 0.9223946248237 |
| "ENSDART00000138467" | 11.8623610216378 | 17.9940050310824 | 0.92238930554907 |
| "ENSDART00000034745" | 29.8957870585032 | 42.5903443128746 | 0.922387306098486 |
| "ENSDART00000086984" | 38.5702110880436 | 49.6216300454013 | 0.922384236675537 |
| "ENSDART00000112106" | 41.2006590035462 | 52.7733917290148 | 0.922382877419823 |
| "ENSDART00000144807" | 268.880267454098 | 309.380899574417 | 0.922366762584576 |
| "ENSDART00000153547" | 69.675594039866 | 87.670734287138 | 0.922358640007577 |
| "ENSDART00000025466" | 25.852759989137 | 33.479978594874 | 0.922345250971926 |
| "ENSDART00000137018" | 10.831902031835 | 17.0837151637032 | 0.922340284677387 |
| "ENSDART00000130032" | 355.073316243737 | 401.361136593749 | 0.922338781711964 |
| "ENSDART00000126075" | 16.8618950040115 | 22.6634729028675 | 0.922321334660229 |
| "ENSDART00000148434" | 791.4315337969 | 895.2712094431 | 0.922305874609834 |
| "ENSDART00000121647" | 153.278971958313 | 182.925762458998 | 0.922292520997124 |
| "ENSDART00000125045" | 184.686108852435 | 219.629656393085 | 0.922289958599036 |
| "ENSDART00000012018" | 210.53592129615 | 247.81363257202 | 0.922281730560985 |
| "ENSDART00000112643" | 15.8721800435886 | 22.9489039560489 | 0.922274841492658 |
| "ENSDART00000154031" | 130.432332888843 | 160.406593616162 | 0.922266016727613 |
| "ENSDART00000080997" | 19.7556470507049 | 26.9658018309434 | 0.922262257370578 |
| "ENSDART00000143480" | 211.036388983232 | 244.971914987942 | 0.922262014532694 |
| "ENSDART00000009200" | 153.7587079522 | 186.100398784311 | 0.922247983195538 |
| "ENSDART00000105903" | 45.6125269665365 | 64.529570949881 | 0.922235803290294 |
| "ENSDART00000112098" | 310.937171386842 | 349.856333895255 | 0.922234479720855 |
| "ENSDART00000139637" | 9.26997796660059 | 15.4558721967825 | 0.922230894258938 |
| "ENSDART00000059327" | 107.441322295188 | 132.754535144844 | 0.92222475477339 |
| "ENSDART00000074602" | 85.3799411528849 | 105.036775865346 | 0.922221942052969 |
| "ENSDART00000144113" | 112.146924223078 | 155.797634723161 | 0.922212881166657 |
| "ENSDART00000131319" | 192.866329750792 | 236.404766119479 | 0.922210839979913 |
| "ENSDART00000080536" | 134.515367101233 | 161.826821899188 | 0.922204567661102 |
| "ENSDART00000104906" | 18.3830750398659 | 26.7348416210575 | 0.922204158157274 |
| "ENSDART00000128283" | 428.38669223265 | 505.905785456319 | 0.922200294318146 |
| "ENSDART00000104493" | 604.924864486727 | 679.190014983018 | 0.922196664758114 |
| "ENSDART00000126255" | 12.1935220054396 | 17.9543461779767 | 0.922195539757585 |
| "ENSDART00000114004" | 78.5284511014981 | 99.0371434926636 | 0.922192517379433 |
| "ENSDART00000083670" | 27.1434920055037 | 37.5040437715487 | 0.922187022731785 |
| "ENSDART00000149660" | 177.164342885906 | 212.546046431512 | 0.922186340307276 |
| "ENSDART00000014007" | 675.812504697616 | 770.516849330428 | 0.922183681179246 |
| "ENSDART00000123608" | 255.089671053449 | 290.92084878576 | 0.922181090156258 |
| "ENSDART00000123076" | 337.545749246713 | 377.274561334572 | 0.922178932960442 |
| "ENSDART00000145541" | 35.4607310500791 | 45.6013872129314 | 0.922177056161905 |
| "ENSDART00000112597" | 32.0351289773112 | 50.459358047587 | 0.922172276898182 |
| "ENSDART00000125208" | 432.825134222099 | 478.516079116543 | 0.922169718887647 |
| "ENSDART00000038942" | 327.280027125366 | 376.468850748251 | 0.922161169516021 |
| "ENSDART00000111758" | 109.868753935215 | 134.018584125631 | 0.922159282295249 |
| "ENSDART00000146883" | 63.088463926807 | 81.9441144129319 | 0.922152492809467 |
| "ENSDART00000155866" | 155.625065128648 | 183.521540605313 | 0.92215120142385 |
| "ENSDART00000129211" | 375.181643402069 | 419.259398873172 | 0.922139355757994 |
| "ENSDART00000150928" | 9.71241900130775 | 15.1470264405737 | 0.922136339360407 |
| "ENSDART00000127930" | 110.021547916786 | 134.931501245057 | 0.922130338777193 |
| "ENSDART00000080604" | 208.960014089055 | 237.224267996863 | 0.922129220145284 |
| "ENSDART00000130222" | 240.306797097599 | 273.584671506831 | 0.922128169309922 |
| "ENSDART00000148507" | 19.3207419979622 | 26.703304842991 | 0.922123898571542 |
| "ENSDART00000149379" | 30.7998940334717 | 40.3242627014547 | 0.92212343116252 |
| "ENSDART00000145016" | 37.4062160371542 | 46.5496634546286 | 0.922118049815424 |
| "ENSDART00000006120" | 94.1339109586259 | 118.37714571396 | 0.922089153544021 |
| "ENSDART00000126784" | 10.355901014 | 16.1598083982924 | 0.922087134960531 |
| "ENSDART00000050898" | 128.188125064095 | 152.699800570346 | 0.922083005871956 |
| "ENSDART00000147181" | 162.045836083848 | 188.357940357592 | 0.922073526341459 |
| "ENSDART00000108810" | 20.5035770037628 | 28.9201701152087 | 0.922072291640546 |
| "ENSDART00000153944" | 14.1575280266441 | 24.1861965274021 | 0.922071852009567 |
| "ENSDART00000090689" | 85.6148940497102 | 107.767642216315 | 0.922065711640906 |
| "ENSDART00000135362" | 17.0595199267267 | 26.0550345769348 | 0.922060884242212 |
| "ENSDART00000134748" | 48.9004400365926 | 61.7222544636468 | 0.92205547897005 |
| "ENSDART00000087163" | 10.4117170073089 | 14.2577610919055 | 0.922053482978147 |
| "ENSDART00000141943" | 64.4229710771806 | 80.3155504893135 | 0.92205169591048 |
| "ENSDART00000079660" | 54.334879071709 | 67.6282972012563 | 0.922048900944118 |
| "ENSDART00000075320" | 291.58740729802 | 336.213396744821 | 0.922038199280298 |
| "ENSDART00000112896" | 201.956433927521 | 235.913458787058 | 0.922032825300827 |
| "ENSDART00000128090" | 48.9442812004607 | 76.9493757394899 | 0.92202846189917 |
| "ENSDART00000057441" | 654.21368924328 | 709.020537640304 | 0.922022936922557 |
| "ENSDART00000099041" | 15.0502980137353 | 21.4450549848082 | 0.9220147417713 |
| "ENSDART00000135083" | 26.1130330157009 | 34.700068991721 | 0.922001501860217 |
| "ENSDART00000141454" | 144.04597003011 | 173.519456679629 | 0.922001125703668 |
| "ENSDART00000047077" | 21.4967080194556 | 29.5249591839544 | 0.921992039674525 |
| "ENSDART00000074426" | 250.517506141854 | 280.366362695077 | 0.921991992486836 |
| "ENSDART00000112921" | 22.519631027294 | 32.3196167965848 | 0.921990588130336 |
| "ENSDART00000062175" | 97.3131901317606 | 124.140079483696 | 0.921986407278862 |
| "ENSDART00000123655" | 27.749261093443 | 39.0518513267294 | 0.921983024322066 |
| "ENSDART00000080633" | 1223.32587945866 | 1321.82272831147 | 0.921976691458999 |
| "ENSDART00000152657" | 27.6530530064665 | 36.888265056818 | 0.921975664095527 |
| "ENSDART00000048775" | 458.474240094198 | 511.820769939323 | 0.921953485584552 |
| "ENSDART00000088419" | 126.909081971318 | 160.927528180131 | 0.921951465944341 |
| "ENSDART00000019206" | 199.551610233387 | 231.889870997013 | 0.921948944407319 |
| "ENSDART00000151777" | 13.1681650019335 | 19.2453918870967 | 0.921947822001526 |
| "ENSDART00000038273" | 91.6883432351118 | 118.910261764904 | 0.921944995884667 |
| "ENSDART00000123621" | 1251.39747864228 | 1313.83264432438 | 0.921941996730157 |
| "ENSDART00000105615" | 4391.20460740918 | 4633.00485569701 | 0.921939837830663 |
| "ENSDART00000141275" | 324.331833813283 | 370.13211812417 | 0.921937607360865 |
| "ENSDART00000049331" | 103.45896803218 | 124.042367761989 | 0.921924900838358 |
| "ENSDART00000053686" | 68.6568239736528 | 86.8858738264962 | 0.921920173738768 |
| "ENSDART00000150574" | 77.5881379894178 | 103.16895851494 | 0.921917721323546 |
| "ENSDART00000074895" | 179.524771133815 | 211.06489445777 | 0.921907587342669 |
| "ENSDART00000064646" | 107.687293259108 | 141.995759166707 | 0.921903489443106 |
| "ENSDART00000155595" | 266.928335307126 | 299.640186721564 | 0.92190027721877 |
| "ENSDART00000044423" | 579.118794162088 | 650.71118211654 | 0.921892029896422 |
| "ENSDART00000154417" | 12.2044740426739 | 22.7396827192338 | 0.921887809713365 |
| "ENSDART00000067857" | 64.5274520324769 | 79.7530507768891 | 0.921878912930463 |
| "ENSDART00000153417" | 18.7738200079588 | 27.643459009649 | 0.92187118078131 |
| "ENSDART00000139924" | 241.860448294514 | 276.02796019037 | 0.921861526006563 |
| "ENSDART00000140286" | 38.540419095898 | 50.895473580015 | 0.921852799989346 |
| "ENSDART00000057865" | 170.376706334572 | 206.132623353266 | 0.921843665744939 |
| "ENSDART00000144076" | 24.8332530365685 | 33.1536935964288 | 0.921840793092004 |
| "ENSDART00000067035" | 51.1997269682935 | 64.527661403363 | 0.921829940427892 |
| "ENSDART00000142479" | 14.8492570357502 | 21.0872332082964 | 0.921820731053268 |
| "ENSDART00000014103" | 82.0259969386408 | 101.166091597001 | 0.921808249654478 |
| "ENSDART00000123667" | 1422.4807660739 | 1493.73398730419 | 0.921804986578987 |
| "ENSDART00000135353" | 10.1213000528871 | 16.801388749198 | 0.921800605120295 |
| "ENSDART00000057697" | 108.655038115201 | 130.2627510788 | 0.921792686708745 |
| "ENSDART00000104272" | 33.0799560596184 | 44.6961147819152 | 0.921791105197893 |
| "ENSDART00000098041" | 11.2264149909101 | 18.0076219291141 | 0.921783338865262 |
| "ENSDART00000098033" | 119.077707159745 | 142.489519358143 | 0.921781299058028 |
| "ENSDART00000127552" | 224.006979606726 | 275.969495861006 | 0.921758904723132 |
| "ENSDART00000109332" | 17.1779840238843 | 24.5643251170962 | 0.921752710715074 |
| "ENSDART00000121745" | 15.9057400267164 | 23.2137911283177 | 0.92175186717561 |
| "ENSDART00000053045" | 633.658350143042 | 690.820178943353 | 0.921739652587633 |
| "ENSDART00000020743" | 91.1496610984976 | 112.598135684839 | 0.92173610059905 |
| "ENSDART00000100173" | 393.584563659708 | 447.815775002247 | 0.921711341544244 |
| "ENSDART00000141090" | 45.091661992627 | 58.8411118430249 | 0.921687901432993 |
| "ENSDART00000155831" | 33.6385339582809 | 52.4330124459084 | 0.921670834381541 |
| "ENSDART00000112270" | 252.054528042923 | 293.893019715276 | 0.921667042950791 |
| "ENSDART00000012940" | 209.402906104266 | 251.883575632659 | 0.92166090834305 |
| "ENSDART00000129123" | 17.6132410123393 | 25.1817763106146 | 0.921657962495868 |
| "ENSDART00000079452" | 152.862873845551 | 197.461198197341 | 0.92164543831516 |
| "ENSDART00000061735" | 45.0950780478969 | 57.385998573188 | 0.921626834728021 |
| "ENSDART00000031135" | 346.239079261026 | 396.127733598482 | 0.921625963899391 |
| "ENSDART00000097719" | 985.015763933725 | 1049.0428126177 | 0.921623283335489 |
| "ENSDART00000149355" | 361.756707201351 | 430.321920335322 | 0.921621366275858 |
| "ENSDART00000134750" | 66.9791149801754 | 86.206540917834 | 0.921614206128228 |
| "ENSDART00000144329" | 32.6378669606236 | 44.3652897337364 | 0.92161133713472 |
| "ENSDART00000156384" | 14.4585120676573 | 22.0861579490843 | 0.921595829177191 |
| "ENSDART00000152974" | 60.2200320098522 | 73.8517851567433 | 0.921594114730995 |
| "ENSDART00000063571" | 22.8956559671702 | 33.8184483314625 | 0.921592922568451 |
| "ENSDART00000123035" | 29.6947460805181 | 42.2425574090889 | 0.921581413889454 |
| "ENSDART00000035713" | 23.6579540127324 | 31.9555824915518 | 0.921569885041575 |
| "ENSDART00000085913" | 354.505612016263 | 400.60350992917 | 0.921566849813909 |
| "ENSDART00000149295" | 17.5947529931404 | 26.3992394554149 | 0.921565540341773 |
| "ENSDART00000100032" | 346.343527201392 | 391.206511563492 | 0.92156491822003 |
| "ENSDART00000088346" | 153.304995959476 | 182.576131934561 | 0.921556920235531 |
| "ENSDART00000153622" | 338.567783565826 | 433.316212162049 | 0.921543256715737 |
| "ENSDART00000136529" | 14.7526970130613 | 20.1633264428856 | 0.921534546295412 |
| "ENSDART00000022546" | 61.2346821493709 | 76.3211125441874 | 0.921532475700823 |
| "ENSDART00000090377" | 34.9070099644665 | 49.007283490559 | 0.921528631707468 |
| "ENSDART00000020769" | 22.6535190240929 | 29.4841052386905 | 0.921526863004822 |
| "ENSDART00000131793" | 15.4150189806648 | 20.7736103346238 | 0.921520797424683 |
| "ENSDART00000078156" | 213.436857389096 | 244.431868702948 | 0.921516699873129 |
| "ENSDART00000136208" | 24.7702529970075 | 32.2708778440121 | 0.921515520055603 |
| "ENSDART00000149384" | 68.1691340322282 | 85.3566973545413 | 0.921511495244123 |
| "ENSDART00000144239" | 23.0884240768356 | 34.7218112159607 | 0.921507969632021 |
| "ENSDART00000114578" | 48.0189740224482 | 63.233507804398 | 0.921502494180847 |
| "ENSDART00000144485" | 14.8232330345868 | 20.4874615759137 | 0.921491224223033 |
| "ENSDART00000111079" | 45.6902470343141 | 58.6357129504146 | 0.921477594067459 |
| "ENSDART00000029588" | 690.466065820927 | 816.641814615221 | 0.921460052000356 |
| "ENSDART00000137653" | 16.7321269339072 | 26.0464351152661 | 0.921459676890023 |
| "ENSDART00000153884" | 16.7691029723049 | 24.7742608082711 | 0.921458854876756 |
| "ENSDART00000139664" | 187.551208273325 | 228.712070609494 | 0.921458011788514 |
| "ENSDART00000123597" | 39.9095750514671 | 51.8482898714458 | 0.921448362339249 |
| "ENSDART00000151919" | 8.89772101770659 | 14.279262997246 | 0.921443865531224 |
| "ENSDART00000132820" | 135.794795144653 | 165.824673978925 | 0.921435386492271 |
| "ENSDART00000081774" | 49.7638029821812 | 63.0159274248134 | 0.921435378770623 |
| "ENSDART00000146109" | 128.515166121202 | 148.908608401245 | 0.921431073861117 |
| "ENSDART00000100351" | 145.198628093122 | 170.436023056242 | 0.921430058982352 |
| "ENSDART00000145330" | 1892.35459456651 | 2208.79650637313 | 0.921429443097171 |
| "ENSDART00000007741" | 59.1149170718293 | 73.1896576737562 | 0.921429409708385 |
| "ENSDART00000087204" | 210.239090196762 | 246.377160138916 | 0.921407197844522 |
| "ENSDART00000099862" | 42.7652952526258 | 69.6383940956736 | 0.921404572073155 |
| "ENSDART00000128763" | 40.4051529105685 | 59.6919728542749 | 0.921387907255705 |
| "ENSDART00000027428" | 640.337535401452 | 696.298471793642 | 0.921383843188907 |
| "ENSDART00000137799" | 47.7964139206373 | 63.2906091509091 | 0.92137817630557 |
| "ENSDART00000106448" | 9.67885901817995 | 14.2417540095575 | 0.921377451037648 |
| "ENSDART00000137769" | 55.0979140036264 | 72.6546288251253 | 0.921355377683609 |
| "ENSDART00000140680" | 13.6849100491484 | 20.1824381639097 | 0.921353104927284 |
| "ENSDART00000018245" | 130.175475917549 | 157.058990526034 | 0.921346819585188 |
| "ENSDART00000150376" | 701.540230385197 | 769.44321285903 | 0.921337894740212 |
| "ENSDART00000153639" | 17.5536570280481 | 25.7767708255335 | 0.921333871270499 |
| "ENSDART00000003494" | 239.751617724862 | 298.297119776994 | 0.921333389883175 |
| "ENSDART00000087976" | 147.896514895662 | 189.200689047309 | 0.921329430813133 |
| "ENSDART00000078358" | 174.72525533722 | 207.187457599575 | 0.921323678628709 |
| "ENSDART00000016582" | 381.769173951357 | 464.463071639924 | 0.921323484268889 |
| "ENSDART00000026924" | 11.5274320468539 | 17.6896993246071 | 0.921323048548585 |
| "ENSDART00000004717" | 17.3156400116654 | 23.5472434285432 | 0.921320035593915 |
| "ENSDART00000086443" | 161.804084091413 | 191.692407187486 | 0.92131642559378 |
| "ENSDART00000133959" | 157.311013975514 | 186.350296322189 | 0.921308914496664 |
| "ENSDART00000066892" | 71.3970781673502 | 90.8518828834009 | 0.921305478389356 |
| "ENSDART00000143225" | 226.797724470834 | 278.731652168804 | 0.921298906864739 |
| "ENSDART00000012963" | 85.3912451258317 | 105.446441233107 | 0.921293984091398 |
| "ENSDART00000148989" | 17.8851699475622 | 30.0771902070915 | 0.921289814138743 |
| "ENSDART00000149045" | 216.625916218192 | 245.638342201764 | 0.92128096997696 |
| "ENSDART00000064365" | 234.697407115522 | 271.417977347074 | 0.92127728600041 |
| "ENSDART00000058831" | 60.2347520380688 | 73.8560848875776 | 0.921275633120212 |
| "ENSDART00000079164" | 67.9126620115771 | 85.9992357298747 | 0.921272428671766 |
| "ENSDART00000074997" | 171.618656814815 | 215.174015733716 | 0.9212710271868 |
| "ENSDART00000146322" | 810.36209838141 | 905.593197948013 | 0.921266884514119 |
| "ENSDART00000135794" | 41.9071410557995 | 56.7501501130052 | 0.921252532322752 |
| "ENSDART00000152109" | 31.7590801153937 | 45.6169169086499 | 0.921249642449235 |
| "ENSDART00000098962" | 156.801485989482 | 187.385297890777 | 0.921234766941485 |
| "ENSDART00000121765" | 25.982176123529 | 36.5949489963277 | 0.921230416579075 |
| "ENSDART00000145691" | 98.4073530325492 | 124.308035314485 | 0.921222337619264 |
| "ENSDART00000059970" | 39.531189863458 | 63.3878434859864 | 0.921219698307781 |
| "ENSDART00000045881" | 553.628192545102 | 618.123329938942 | 0.921201696819609 |
| "ENSDART00000125012" | 46.5867850123875 | 62.4574871243241 | 0.921194739205551 |
| "ENSDART00000016090" | 342.375943510876 | 383.283813868049 | 0.921194694908957 |
| "ENSDART00000085666" | 803.470084007088 | 958.265546628809 | 0.921191393273808 |
| "ENSDART00000017110" | 1283.85170382205 | 1337.46088444026 | 0.921191025735593 |
| "ENSDART00000067600" | 13.5102780229696 | 20.7597531176929 | 0.921187475130976 |
| "ENSDART00000156541" | 18.584116017851 | 25.1371000211459 | 0.921179162879004 |
| "ENSDART00000108667" | 19.0378610255049 | 24.5086591816425 | 0.921179110280604 |
| "ENSDART00000149580" | 354.159060147751 | 407.598959152796 | 0.921172180372532 |
| "ENSDART00000138672" | 12.9448680137674 | 18.6140834766475 | 0.92116627387556 |
| "ENSDART00000051528" | 86.3847280772368 | 108.292702118087 | 0.921165830410476 |
| "ENSDART00000141314" | 47.5648440641514 | 62.9260974592466 | 0.921160960371498 |
| "ENSDART00000155395" | 9.30730594071063 | 18.6599548582743 | 0.921157484146566 |
| "ENSDART00000143448" | 11.010686999639 | 17.3626966206332 | 0.921153951134011 |
| "ENSDART00000102275" | 25.5993190731129 | 35.7123735628102 | 0.921149914043531 |
| "ENSDART00000064672" | 282.246325755253 | 328.582810264203 | 0.921145758150832 |
| "ENSDART00000020859" | 107.48727507333 | 135.377240189448 | 0.921144942496956 |
| "ENSDART00000147962" | 86.9573221326912 | 106.746086403896 | 0.921141302983427 |
| "ENSDART00000130765" | 18.6399320111599 | 31.3780272188694 | 0.921136699555373 |
| "ENSDART00000154333" | 47.5357889583611 | 63.5387715353012 | 0.921128996960322 |
| "ENSDART00000038574" | 137.484127031859 | 160.736463891081 | 0.921120353097262 |
| "ENSDART00000137364" | 1240.96616726436 | 1335.16326930195 | 0.921114656562213 |
| "ENSDART00000153487" | 319.452356488217 | 441.820491404033 | 0.921114492847167 |
| "ENSDART00000110927" | 67.5667479846282 | 88.501772374173 | 0.921111629540733 |
| "ENSDART00000110679" | 174.883225052623 | 206.89008212715 | 0.921090280159205 |
| "ENSDART00000123114" | 186.220183208083 | 215.612281131561 | 0.921085740281587 |
| "ENSDART00000123922" | 13.1349569545181 | 22.0861579490843 | 0.921079787871764 |
| "ENSDART00000005740" | 38.4438590732092 | 49.6154175168801 | 0.921065728455676 |
| "ENSDART00000111733" | 297.112245101779 | 374.572923759065 | 0.921061524310032 |
| "ENSDART00000063245" | 33.3737890693101 | 43.0792614609795 | 0.921059431888426 |
| "ENSDART00000124627" | 350.639192270566 | 385.705057333751 | 0.921053016102685 |
| "ENSDART00000139874" | 358.561467375562 | 414.219042141678 | 0.921051864113205 |
| "ENSDART00000146247" | 44.6714769681009 | 60.4316861411563 | 0.921041074292092 |
| "ENSDART00000011931" | 251.191868968759 | 302.19784273765 | 0.921039727541158 |
| "ENSDART00000143974" | 27.0054510670797 | 36.0319686461048 | 0.921035994935528 |
| "ENSDART00000092687" | 178.873049267733 | 214.415602186573 | 0.921035974818382 |
| "ENSDART00000090528" | 626.626684910346 | 682.553516688329 | 0.921035078617718 |
| "ENSDART00000125232" | 91.1432139386007 | 111.035038752839 | 0.921034838442781 |
| "ENSDART00000052289" | 61.4768190924482 | 76.8800302313063 | 0.921032956466659 |
| "ENSDART00000036680" | 108.855159602833 | 145.468077209212 | 0.921032444302499 |
| "ENSDART00000111652" | 126.305992052294 | 151.156292746 | 0.921027687575823 |
| "ENSDART00000073604" | 21.7758210009307 | 31.4000097620083 | 0.92102212804097 |
| "ENSDART00000154066" | 216.277290007397 | 247.976811285345 | 0.921019015645264 |
| "ENSDART00000133756" | 46.2001929859198 | 62.4668042915214 | 0.921003876725205 |
| "ENSDART00000074968" | 125.000221086928 | 147.902038972047 | 0.921002013393417 |
| "ENSDART00000080673" | 1573.56459986275 | 1697.96119535921 | 0.920987085493987 |
| "ENSDART00000124132" | 80.492809058415 | 110.86708292205 | 0.92098600281352 |
| "ENSDART00000153566" | 17.6170090033215 | 22.9699284747599 | 0.920974390081349 |
| "ENSDART00000157351" | 38.7040990848425 | 53.9748477914671 | 0.920964747823889 |
| "ENSDART00000117148" | 101.706570075552 | 125.465529541829 | 0.920956524717655 |
| "ENSDART00000088168" | 574.700144632833 | 658.899705501666 | 0.920932347380409 |
| "ENSDART00000130877" | 233.952508267091 | 280.190357217454 | 0.920913455016766 |
| "ENSDART00000064809" | 107.212666969192 | 128.170357188892 | 0.920911051889754 |
| "ENSDART00000111359" | 73.406652016054 | 91.8262010801721 | 0.920900258811252 |
| "ENSDART00000141188" | 31.2423350681788 | 40.2929662422874 | 0.92088763345394 |
| "ENSDART00000000698" | 10.2031400473593 | 17.4436868056322 | 0.920883962592736 |
| "ENSDART00000130355" | 7.07517199019599 | 14.5859588880377 | 0.92087628872921 |
| "ENSDART00000048867" | 9.64188297978225 | 14.2897752566015 | 0.920871734265216 |
| "ENSDART00000135070" | 21.7316609162808 | 36.5748792508757 | 0.920859466000934 |
| "ENSDART00000141446" | 431.571862239565 | 495.64734139183 | 0.920850044658955 |
| "ENSDART00000067131" | 26.2397369662476 | 36.039135947885 | 0.920849557278424 |
| "ENSDART00000098942" | 28.6646720413582 | 39.1149248828625 | 0.920848630755292 |
| "ENSDART00000153087" | 47.2340680309926 | 66.1096329886569 | 0.920836796070787 |
| "ENSDART00000152137" | 18.1077300493731 | 25.4105866550833 | 0.92082235555654 |
| "ENSDART00000134468" | 10.7908060667427 | 17.3512295880187 | 0.920821922087421 |
| "ENSDART00000153154" | 21.6905649511886 | 31.6646566153779 | 0.920817280090165 |
| "ENSDART00000122915" | 55.7560830296047 | 70.4403940561232 | 0.920810208394747 |
| "ENSDART00000142082" | 10.0730200415427 | 14.5620667983808 | 0.920782081220078 |
| "ENSDART00000101536" | 51.4000310599233 | 66.4275588443327 | 0.92077526370127 |
| "ENSDART00000134950" | 15.7978760310808 | 24.2055485673254 | 0.920771500274591 |
| "ENSDART00000115259" | 187.262298106545 | 225.760624456261 | 0.920762952822993 |
| "ENSDART00000081015" | 103.351104036545 | 127.873218784197 | 0.920759307081724 |
| "ENSDART00000122579" | 293.898954965012 | 330.785821570659 | 0.920757202513507 |
| "ENSDART00000012552" | 100.661424072463 | 127.347032967303 | 0.920755875077058 |
| "ENSDART00000150852" | 23.6432339845158 | 34.112004710852 | 0.920750696675137 |
| "ENSDART00000086795" | 13.6256780005696 | 20.1886506924309 | 0.920750479355394 |
| "ENSDART00000080709" | 103.325750891875 | 131.045290532162 | 0.920749161656149 |
| "ENSDART00000143771" | 18.0409620188298 | 24.7919403694068 | 0.920747550892581 |
| "ENSDART00000089408" | 154.844279063887 | 178.737395905045 | 0.920737294924797 |
| "ENSDART00000104838" | 13.651702001733 | 22.7272609133603 | 0.920736502844592 |
| "ENSDART00000092073" | 385.590395334612 | 437.567504164556 | 0.920735146494679 |
| "ENSDART00000124103" | 54.4578480462041 | 68.5342840866323 | 0.920733388576731 |
| "ENSDART00000155081" | 21.187803045835 | 28.8728665736933 | 0.92073133800633 |
| "ENSDART00000061365" | 650.882957801562 | 715.738809737522 | 0.920727858124605 |
| "ENSDART00000127691" | 227.871523099725 | 262.938442652893 | 0.92072189136599 |
| "ENSDART00000143500" | 16.2625730759691 | 23.2522548892652 | 0.920720688333015 |
| "ENSDART00000102411" | 207.596869798464 | 248.959491876055 | 0.920716628067311 |
| "ENSDART00000152314" | 13.2609570336401 | 21.1223520116685 | 0.920710157472736 |
| "ENSDART00000013635" | 29.8252510369777 | 37.830328769994 | 0.920702708273866 |
| "ENSDART00000090171" | 401.879997674607 | 480.425195011052 | 0.92070179188208 |
| "ENSDART00000157017" | 121.737210181037 | 151.691555411192 | 0.920699462251406 |
| "ENSDART00000131632" | 18.7180040146499 | 25.1232428042151 | 0.920689783231786 |
| "ENSDART00000122120" | 157.193183462859 | 198.645866797219 | 0.920687370348087 |
| "ENSDART00000111258" | 21.0987790051106 | 30.9650201447023 | 0.920685832806417 |
| "ENSDART00000151942" | 17.1410079854866 | 23.9186821030866 | 0.920684872794176 |
| "ENSDART00000100685" | 67.5787888439302 | 87.8109756840653 | 0.920682605352996 |
| "ENSDART00000136040" | 13.573629998243 | 20.5135035309877 | 0.92066773998717 |
| "ENSDART00000146074" | 633.447782400016 | 711.794489983666 | 0.920657911107921 |
| "ENSDART00000076502" | 62.8418551213227 | 78.1080056354621 | 0.920657124782953 |
| "ENSDART00000156839" | 311.783636660078 | 358.775161188745 | 0.920653919509431 |
| "ENSDART00000025031" | 205.059715439448 | 237.371913763301 | 0.920647299618869 |
| "ENSDART00000146576" | 319.745802333963 | 359.218203704054 | 0.920643802418326 |
| "ENSDART00000153428" | 560.313609344481 | 607.613210641792 | 0.92064038356168 |

**Table S5**. Top (1,000) down-regulated transcripts after NaBu treatment

| **Transcript ID** | **Control TMM mean** | **NaBu TMM mean** | **Noiseq probability** |
| --- | --- | --- | --- |
| "ENSDART00000035031" | 1238.37929213622 | 965.902559321108 | 1 |
| "ENSDART00000041142" | 1191.31398078322 | 941.953881067008 | 0.999999999999985 |
| "ENSDART00000059228" | 4383.03787647249 | 3654.90020381762 | 0.99999999999998 |
| "ENSDART00000067817" | 6896.56507641841 | 5916.60763007956 | 0.999999999999945 |
| "ENSDART00000020834" | 19497.0565997468 | 14063.2433531654 | 0.999999999999886 |
| "ENSDART00000050753" | 3304.96998921525 | 2723.35467804284 | 0.99999996749396 |
| "ENSDART00000027793" | 702.761532731451 | 521.915485475652 | 0.99999751349915 |
| "ENSDART00000101043" | 535.695881952538 | 351.729945811082 | 0.999996597720434 |
| "ENSDART00000143888" | 10631.4812592006 | 8809.1560565835 | 0.99945175281097 |
| "ENSDART00000074692" | 799.662810707005 | 562.833481674 | 0.992198961944864 |
| "ENSDART00000004756" | 1579.86128205021 | 1279.4917633432 | 0.982360055758714 |
| "ENSDART00000075539" | 1252.00978270639 | 841.499880534347 | 0.96141216862952 |
| "ENSDART00000042895" | 2595.74840880157 | 2144.74218897837 | 0.956207622299696 |
| "ENSDART00000130501" | 1874.21348868067 | 1053.85385270786 | 0.930968144700635 |
| "ENSDART00000105594" | 1983.92448033684 | 1528.44385700179 | 0.930940267959533 |
| "ENSDART00000010282" | 968.837006149718 | 756.654652819284 | 0.930805750802233 |
| "ENSDART00000110690" | 179.070988854168 | 79.8070442335551 | 0.930794766242239 |
| "ENSDART00000146460" | 3456.75914866176 | 2536.13138935811 | 0.930619681670495 |
| "ENSDART00000037371" | 823.431142809635 | 640.976372296273 | 0.929778601231368 |
| "ENSDART00000141125" | 40.2962000928654 | 28.8387025435802 | 0.927951459045452 |
| "ENSDART00000001830" | 893.727676946161 | 809.984920837166 | 0.927950298755065 |
| "ENSDART00000078476" | 31.4177039807129 | 22.0173525022284 | 0.927949388027437 |
| "ENSDART00000103124" | 1765.29661202442 | 1641.9604611517 | 0.927949028933922 |
| "ENSDART00000147937" | 354.315639649649 | 303.833573963049 | 0.927946046013799 |
| "ENSDART00000023059" | 51.1661339702351 | 34.717985620587 | 0.927945306798804 |
| "ENSDART00000144591" | 34.761433044078 | 23.6236935638087 | 0.927944617498977 |
| "ENSDART00000065681" | 1588.1978933761 | 1407.87454837241 | 0.927943020926122 |
| "ENSDART00000042237" | 230.437145267244 | 199.18018119149 | 0.927942609741289 |
| "ENSDART00000024644" | 56.0355479467921 | 38.4425222082502 | 0.927941420807386 |
| "ENSDART00000102932" | 759.240883168586 | 663.268943334756 | 0.927940925076951 |
| "ENSDART00000104555" | 19.737159031506 | 14.5780738807288 | 0.927939774219978 |
| "ENSDART00000154197" | 45.0984610882362 | 32.8835519200667 | 0.927939265257206 |
| "ENSDART00000122725" | 571.934818943256 | 507.229599074623 | 0.927938701187762 |
| "ENSDART00000112639" | 26.0938081101467 | 16.7414230829099 | 0.927938426098097 |
| "ENSDART00000002633" | 235.713196621384 | 188.407571408725 | 0.927938387744955 |
| "ENSDART00000063151" | 324.051214044167 | 278.361892475386 | 0.927938020148153 |
| "ENSDART00000103795" | 147.822778437795 | 104.352198206098 | 0.927937410826302 |
| "ENSDART00000149503" | 16.2591570206992 | 10.2370408728062 | 0.927936412163796 |
| "ENSDART00000114796" | 252.459157108085 | 212.504478031888 | 0.927936366360549 |
| "ENSDART00000111717" | 178.464201231084 | 145.623295235854 | 0.927935804646062 |
| "ENSDART00000136832" | 19.3946940747576 | 12.3877279502146 | 0.927934755548341 |
| "ENSDART00000009382" | 238.821504278075 | 203.324352093772 | 0.927934056112248 |
| "ENSDART00000108543" | 115.134656168337 | 92.8076833275546 | 0.927933456679009 |
| "ENSDART00000059174" | 51.3770381633869 | 36.2751135941339 | 0.927931951523136 |
| "ENSDART00000100869" | 1288.7525001376 | 1217.33715951897 | 0.927928841719012 |
| "ENSDART00000156044" | 146.224967156231 | 123.463380329419 | 0.927927224255012 |
| "ENSDART00000145816" | 67.7787409998477 | 50.1879553531995 | 0.927924990008075 |
| "ENSDART00000135040" | 130.206440290968 | 99.8224845911037 | 0.927921250371653 |
| "ENSDART00000125433" | 7343.5547573541 | 4121.99363164784 | 0.927921090563082 |
| "ENSDART00000123327" | 38.0172268887863 | 23.0222494526383 | 0.927919446987043 |
| "ENSDART00000134321" | 212.487032997561 | 173.15255480365 | 0.927913703690427 |
| "ENSDART00000032887" | 795.471945513909 | 734.367642529661 | 0.927910903632055 |
| "ENSDART00000105661" | 81.5817301955107 | 61.7473383942929 | 0.927904798520396 |
| "ENSDART00000139218" | 94.5050790854523 | 74.6786946548071 | 0.927903599131197 |
| "ENSDART00000137034" | 28.043446038847 | 21.1032402906444 | 0.927902364978199 |
| "ENSDART00000100557" | 113.06658715741 | 87.7612787070646 | 0.927899689893712 |
| "ENSDART00000006934" | 39.4218851100425 | 26.1078329414426 | 0.927898615376961 |
| "ENSDART00000142804" | 282.458688235529 | 238.979864628971 | 0.927897306162481 |
| "ENSDART00000057761" | 26.3838731288562 | 15.5330400375767 | 0.927896464251562 |
| "ENSDART00000016658" | 2095.49398670634 | 1976.04610488434 | 0.9278956938005 |
| "ENSDART00000139062" | 73.789894017113 | 56.7690247662991 | 0.927888099604354 |
| "ENSDART00000074478" | 241.191327231301 | 201.643710762344 | 0.927888076990145 |
| "ENSDART00000135783" | 484.666919974649 | 417.219743924242 | 0.927887077683817 |
| "ENSDART00000143999" | 2223.26025045701 | 1937.48412485258 | 0.927883806492286 |
| "ENSDART00000138473" | 641.165695259487 | 573.050930187984 | 0.927878643748063 |
| "ENSDART00000067036" | 99.1813730166316 | 78.1316574062198 | 0.927875736379826 |
| "ENSDART00000100487" | 17.6988489977937 | 10.5222348582573 | 0.927874708613794 |
| "ENSDART00000136085" | 19.3950460104699 | 12.4295366687375 | 0.927868261252865 |
| "ENSDART00000124945" | 53.1297880557273 | 38.4128982278705 | 0.92786677142265 |
| "ENSDART00000123700" | 749.506421562722 | 646.952099574623 | 0.927866673160569 |
| "ENSDART00000129698" | 14228.0001111469 | 13130.4558536629 | 0.927864804697482 |
| "ENSDART00000132953" | 671.704849800305 | 560.048612112859 | 0.927864646359071 |
| "ENSDART00000128321" | 234.192368521242 | 189.839332650233 | 0.927861270766192 |
| "ENSDART00000007196" | 19.5628789410396 | 10.8511471087492 | 0.927853309327332 |
| "ENSDART00000150571" | 463.963078364552 | 356.343464887217 | 0.927843969882796 |
| "ENSDART00000129336" | 41.2523550701605 | 30.3970223581164 | 0.927838441630186 |
| "ENSDART00000150218" | 16.3149730140081 | 8.64383932262806 | 0.927834879047888 |
| "ENSDART00000151386" | 177.27609144666 | 139.770050862753 | 0.927834332289299 |
| "ENSDART00000152599" | 26.6893620472069 | 18.5832644041096 | 0.927833927684645 |
| "ENSDART00000150219" | 734.350034717499 | 673.628476866318 | 0.927832997812544 |
| "ENSDART00000153687" | 54.92219315538 | 39.3900839955876 | 0.927822153096381 |
| "ENSDART00000148142" | 22.3298940222556 | 15.2086645856494 | 0.927809405363865 |
| "ENSDART00000024244" | 111.681974287269 | 86.8070335069144 | 0.927804723757739 |
| "ENSDART00000145112" | 227.184063606381 | 186.626413459342 | 0.927804279332437 |
| "ENSDART00000008607" | 272.504550256718 | 233.377887278937 | 0.92780291163779 |
| "ENSDART00000121509" | 445.721068897373 | 377.689787451192 | 0.927784277785258 |
| "ENSDART00000137438" | 349.132430871491 | 287.896603699603 | 0.927781563358064 |
| "ENSDART00000113196" | 803.800791966627 | 715.475716515882 | 0.9277806853647 |
| "ENSDART00000007013" | 225.636507633651 | 182.671634367322 | 0.927778151204422 |
| "ENSDART00000157340" | 525.926760482499 | 472.47362057738 | 0.927769274772558 |
| "ENSDART00000029646" | 14237.0182257731 | 13119.1408486994 | 0.927768817991452 |
| "ENSDART00000142769" | 3327.02187865121 | 3066.52440071557 | 0.927764940368534 |
| "ENSDART00000152960" | 126.120023038237 | 101.065986439809 | 0.927754432360148 |
| "ENSDART00000097357" | 250.208150187729 | 208.763697294146 | 0.927752258560819 |
| "ENSDART00000047834" | 177.147412198624 | 147.0412025116 | 0.927729959632449 |
| "ENSDART00000128379" | 521.0840413636 | 466.739122198203 | 0.927725561696419 |
| "ENSDART00000137533" | 951.458834608341 | 852.388823092436 | 0.927724250303108 |
| "ENSDART00000138324" | 1978.47101022869 | 1647.36012086323 | 0.927716156975948 |
| "ENSDART00000129301" | 207.971504524836 | 164.660190094003 | 0.927715396898874 |
| "ENSDART00000125000" | 174.461918191098 | 135.278981904076 | 0.927706981583735 |
| "ENSDART00000024328" | 690.017857498039 | 447.361775628743 | 0.927683620741506 |
| "ENSDART00000090675" | 519.728967594684 | 471.278380294618 | 0.927672956230338 |
| "ENSDART00000143403" | 43.6444010186373 | 29.3933204998647 | 0.927662202499704 |
| "ENSDART00000111025" | 549.266733737075 | 489.477369506379 | 0.927661145372795 |
| "ENSDART00000141276" | 33.0237551156665 | 20.4955836509528 | 0.927654236308599 |
| "ENSDART00000136871" | 825.459706202317 | 732.857452429334 | 0.927653867152084 |
| "ENSDART00000144452" | 165.005201309156 | 132.060393497161 | 0.927651070935944 |
| "ENSDART00000103498" | 57.1467911239302 | 41.9248718723122 | 0.927647349605772 |
| "ENSDART00000137082" | 1057.69061155448 | 904.057225718524 | 0.927647289735972 |
| "ENSDART00000113176" | 310.398957760076 | 258.468398212928 | 0.927643234038295 |
| "ENSDART00000060905" | 93.2985473116906 | 70.0235613865824 | 0.927639581301128 |
| "ENSDART00000138155" | 111.404302063574 | 89.016728226183 | 0.927636543009201 |
| "ENSDART00000079498" | 19.1303011214992 | 10.5468414022739 | 0.927635838298843 |
| "ENSDART00000089121" | 49.193921110572 | 35.032089132058 | 0.927628524408424 |
| "ENSDART00000138809" | 16.6978300644241 | 9.90860600894372 | 0.927620424269186 |
| "ENSDART00000121848" | 927.698863190298 | 830.152991609724 | 0.927612005390337 |
| "ENSDART00000144067" | 198.350672159031 | 164.812306733138 | 0.927601581741724 |
| "ENSDART00000132642" | 1532.02849061699 | 1307.17248742018 | 0.927573677591515 |
| "ENSDART00000152698" | 53.5865641680081 | 36.8935195609113 | 0.92756503805371 |
| "ENSDART00000007810" | 100.668575103785 | 75.8137981294182 | 0.927564268275178 |
| "ENSDART00000148453" | 814.642609971207 | 690.643656438686 | 0.927558686475957 |
| "ENSDART00000114831" | 82.2999011713537 | 59.7081575808238 | 0.92754918658453 |
| "ENSDART00000103350" | 206.36654221195 | 176.456024916806 | 0.927541911657918 |
| "ENSDART00000145927" | 181.836215498954 | 143.012837604091 | 0.927538972293302 |
| "ENSDART00000139071" | 772.610003375996 | 582.597730687854 | 0.927538677727609 |
| "ENSDART00000155902" | 436.192291676919 | 380.737730100573 | 0.927537697336985 |
| "ENSDART00000098226" | 267.354934476618 | 226.390082743721 | 0.927528081781103 |
| "ENSDART00000052257" | 111.092399000257 | 85.2554003563599 | 0.927520515610511 |
| "ENSDART00000033844" | 76.496940163395 | 56.1073746699415 | 0.927512179204594 |
| "ENSDART00000150496" | 58.4046670285938 | 44.6366265022567 | 0.927511585441073 |
| "ENSDART00000013497" | 1208.65596150352 | 1065.60011139912 | 0.927507639820576 |
| "ENSDART00000085816" | 171.337366189205 | 139.572059590822 | 0.927501998760425 |
| "ENSDART00000076816" | 81.1550649961572 | 60.2253324752871 | 0.927494578148937 |
| "ENSDART00000065101" | 15.1098819980264 | 9.90741091678554 | 0.927492161321989 |
| "ENSDART00000141530" | 135.884908007445 | 107.962594775437 | 0.927490892580213 |
| "ENSDART00000130565" | 140.850497056048 | 114.077855498693 | 0.927489290930241 |
| "ENSDART00000149546" | 99.6524842064861 | 76.608693462786 | 0.927486980745385 |
| "ENSDART00000042410" | 77.9788169276494 | 58.2812361314781 | 0.927476070708781 |
| "ENSDART00000104781" | 27.3848920622258 | 19.8446861328499 | 0.927457443026531 |
| "ENSDART00000103074" | 21.240202983874 | 14.585241182509 | 0.927444434675587 |
| "ENSDART00000028450" | 978.930691854595 | 902.030931092882 | 0.92743730615385 |
| "ENSDART00000100327" | 773.582633803007 | 713.041672571335 | 0.927432498340523 |
| "ENSDART00000083820" | 469.623018576536 | 422.280413971256 | 0.92742179601776 |
| "ENSDART00000033574" | 21.2394991124493 | 12.4412440202512 | 0.927418641258543 |
| "ENSDART00000153674" | 22.3483820414545 | 16.1349615353765 | 0.927416178373925 |
| "ENSDART00000125746" | 95.4211939047921 | 72.8263410742519 | 0.927413144121202 |
| "ENSDART00000024136" | 90.0229940217182 | 65.9103839498695 | 0.927412634562771 |
| "ENSDART00000011540" | 629.504661121686 | 550.645792896307 | 0.927402979891771 |
| "ENSDART00000130424" | 87.7912120069159 | 67.8712018303862 | 0.927384020462904 |
| "ENSDART00000111029" | 353.362229871129 | 301.094819353602 | 0.927382871253584 |
| "ENSDART00000007827" | 126.265248022914 | 102.949399411801 | 0.927378869614741 |
| "ENSDART00000001253" | 210.149648190465 | 167.446175816759 | 0.927345535555535 |
| "ENSDART00000111938" | 143.973960235874 | 118.12199042082 | 0.927345347254937 |
| "ENSDART00000143128" | 4510.30421558991 | 4153.84992842151 | 0.927331468939273 |
| "ENSDART00000134182" | 79.4996120128609 | 57.0169500829609 | 0.927326626641702 |
| "ENSDART00000065941" | 437.571326333241 | 342.463193259734 | 0.92730736575753 |
| "ENSDART00000008326" | 152.245950373732 | 119.401565846156 | 0.927303766259499 |
| "ENSDART00000148518" | 278.817809151995 | 242.790649156895 | 0.927301857832938 |
| "ENSDART00000023278" | 90.9150931523153 | 67.5138606916728 | 0.927274657408428 |
| "ENSDART00000099846" | 371.469744301574 | 306.476240285941 | 0.927270081017014 |
| "ENSDART00000022646" | 735.164446795249 | 665.716538251467 | 0.927269759753604 |
| "ENSDART00000138632" | 54.9334971283267 | 41.2362250476216 | 0.927261170063097 |
| "ENSDART00000103973" | 103.58806524579 | 71.7108267072945 | 0.927254487718032 |
| "ENSDART00000060243" | 134.24682120635 | 109.771226839783 | 0.927253072873963 |
| "ENSDART00000138581" | 478.471759968535 | 393.595230690078 | 0.927247282357542 |
| "ENSDART00000055611" | 206.622695311819 | 174.829377042043 | 0.927240650023217 |
| "ENSDART00000104921" | 68.2958379827749 | 49.5387270627154 | 0.92723668176043 |
| "ENSDART00000124219" | 15.7756200208997 | 9.90024361500537 | 0.927235310891612 |
| "ENSDART00000141304" | 773.349002876523 | 663.702533579964 | 0.927233465846848 |
| "ENSDART00000080664" | 498.002515427165 | 409.425425664296 | 0.927225615849071 |
| "ENSDART00000007577" | 58.9625080409009 | 44.9361550912682 | 0.927225398536775 |
| "ENSDART00000147083" | 25.673975021333 | 17.3626966206332 | 0.927220831055057 |
| "ENSDART00000110301" | 63.3185600105823 | 48.3690480972288 | 0.927211381765019 |
| "ENSDART00000078576" | 799.918277464794 | 714.216918788019 | 0.927202052524743 |
| "ENSDART00000129337" | 1579.39810506525 | 1462.04668273495 | 0.927180982867524 |
| "ENSDART00000123973" | 22.0247570396172 | 14.9060313579617 | 0.927179696750172 |
| "ENSDART00000124939" | 19.7744870056161 | 13.3338543264947 | 0.927150854387095 |
| "ENSDART00000090669" | 1369.97980807627 | 1265.60066380837 | 0.92712898312725 |
| "ENSDART00000135047" | 22.7576150287462 | 15.4976809153053 | 0.927121459558036 |
| "ENSDART00000052080" | 1246.80327333944 | 1131.48727807328 | 0.927106479287939 |
| "ENSDART00000153214" | 322.812327683481 | 273.539995217362 | 0.927073056418313 |
| "ENSDART00000009223" | 1140.34990632611 | 1057.84085577018 | 0.927062948995797 |
| "ENSDART00000129940" | 656.392417992894 | 598.454178399424 | 0.927060173961929 |
| "ENSDART00000011138" | 2309.76739356547 | 2029.13088321205 | 0.92703194914656 |
| "ENSDART00000015046" | 225.516569763783 | 181.076276449388 | 0.927026469157552 |
| "ENSDART00000149749" | 89.067223995066 | 69.7378900145018 | 0.927003479256358 |
| "ENSDART00000082409" | 568.617817752479 | 511.215500232779 | 0.927001834151806 |
| "ENSDART00000125289" | 2586.28859257184 | 2344.49172831452 | 0.926969047846979 |
| "ENSDART00000046576" | 23.1853360352367 | 14.9055539713323 | 0.926957105073473 |
| "ENSDART00000005682" | 20116.3856415942 | 17778.460134924 | 0.926927766750168 |
| "ENSDART00000011970" | 811.877433870697 | 732.218673723507 | 0.926899449211249 |
| "ENSDART00000136592" | 1014.93107728922 | 906.421983854518 | 0.926893638290705 |
| "ENSDART00000138333" | 33.7535820001685 | 25.4337610392115 | 0.926888243937808 |
| "ENSDART00000016502" | 1227.15204547123 | 1134.60674923433 | 0.92686363591062 |
| "ENSDART00000087497" | 183.281182254672 | 148.948681966281 | 0.926860132923564 |
| "ENSDART00000127607" | 142.265337413253 | 103.68576123877 | 0.926847619895104 |
| "ENSDART00000065985" | 1048.56605666214 | 909.073772818175 | 0.926818646426337 |
| "ENSDART00000156551" | 332.811395648229 | 274.322402818989 | 0.926807360421898 |
| "ENSDART00000111362" | 315.240020502267 | 265.777710629125 | 0.926780944059511 |
| "ENSDART00000114330" | 339.242617115884 | 298.905431447516 | 0.926772256583245 |
| "ENSDART00000140586" | 1032.35854942085 | 920.774174385254 | 0.926768678426 |
| "ENSDART00000045598" | 238.605776286804 | 199.044476593127 | 0.926763975559211 |
| "ENSDART00000030509" | 26.5479380684436 | 18.6174284342228 | 0.926754931093867 |
| "ENSDART00000137989" | 866.819672377192 | 724.226673411464 | 0.926720727607312 |
| "ENSDART00000113823" | 24.9150930310407 | 17.0385614876051 | 0.926707948192 |
| "ENSDART00000056523" | 282.088927851552 | 179.396415498187 | 0.926702568723974 |
| "ENSDART00000150416" | 27.2736120113204 | 16.4406994017403 | 0.926676648809487 |
| "ENSDART00000034216" | 2941.43573818696 | 2741.51883131143 | 0.926662589759611 |
| "ENSDART00000149264" | 1364.5657530557 | 1266.98417973978 | 0.926640632953819 |
| "ENSDART00000153424" | 151.026876215889 | 114.853158473238 | 0.926632014080251 |
| "ENSDART00000138073" | 231.602614090844 | 195.305368334173 | 0.926622290027274 |
| "ENSDART00000134456" | 400.111071510684 | 349.429466352988 | 0.926588959709656 |
| "ENSDART00000153970" | 17.5242169716149 | 10.5100501201141 | 0.926559126725349 |
| "ENSDART00000123257" | 107.464315191724 | 85.0048510388204 | 0.926558913322121 |
| "ENSDART00000047211" | 83.1225200875623 | 65.4439234803642 | 0.926544391126843 |
| "ENSDART00000024199" | 14680.8497602888 | 12635.722363882 | 0.926508250776974 |
| "ENSDART00000104074" | 5249.77203276801 | 4300.65735219591 | 0.926504480467816 |
| "ENSDART00000136834" | 47.3046040525181 | 34.1679077140359 | 0.926500140685387 |
| "ENSDART00000111567" | 3945.82901399035 | 3657.34167876741 | 0.926478520263468 |
| "ENSDART00000112833" | 1148.78733613147 | 972.246574216472 | 0.926469480912021 |
| "ENSDART00000132140" | 19.0529329894338 | 13.3216695883515 | 0.926467397363274 |
| "ENSDART00000112092" | 35.620291112329 | 25.434238425841 | 0.926437216558043 |
| "ENSDART00000149350" | 645.058308338063 | 587.497365384623 | 0.926423083118391 |
| "ENSDART00000077578" | 385.436479516043 | 341.479765426874 | 0.926410320946463 |
| "ENSDART00000080106" | 219.681021020627 | 172.869030045818 | 0.92637336913848 |
| "ENSDART00000046959" | 592.397370268849 | 526.842060728393 | 0.926360363281532 |
| "ENSDART00000103153" | 646.768539036875 | 562.510946591555 | 0.926358683604772 |
| "ENSDART00000003666" | 428.669117967541 | 367.48869480562 | 0.926343983049483 |
| "ENSDART00000003665" | 535.748398464714 | 456.976657367604 | 0.926334475848614 |
| "ENSDART00000122648" | 264.281242610192 | 210.031805686868 | 0.926329002978451 |
| "ENSDART00000132883" | 58.0399130467336 | 43.7447339015419 | 0.926295039900846 |
| "ENSDART00000084223" | 762.88091780685 | 694.269388527597 | 0.926285125384488 |
| "ENSDART00000080269" | 1374.5485765057 | 1158.34012169728 | 0.926282192821197 |
| "ENSDART00000144171" | 239.411530545591 | 193.370348838662 | 0.926270122156636 |
| "ENSDART00000123044" | 127.846012043059 | 103.178450075169 | 0.926262080347866 |
| "ENSDART00000150703" | 18.3347950285215 | 13.0178412685057 | 0.926236381895651 |
| "ENSDART00000020256" | 874.22813245105 | 739.859199890463 | 0.926230920072315 |
| "ENSDART00000148446" | 110.514244260328 | 80.7719760860928 | 0.926208518704588 |
| "ENSDART00000084007" | 54.6064560712196 | 39.6181766345277 | 0.926181308367503 |
| "ENSDART00000133882" | 606.957415746381 | 553.383757372019 | 0.926142115300882 |
| "ENSDART00000152145" | 41.9256290749983 | 29.5106245803941 | 0.926137534847079 |
| "ENSDART00000153421" | 240.698010575541 | 195.109526927659 | 0.926123980342094 |
| "ENSDART00000144959" | 30.0481630745009 | 19.8164943123587 | 0.926113521353261 |
| "ENSDART00000007197" | 717.04288523684 | 589.221257347969 | 0.926089525781114 |
| "ENSDART00000125198" | 2060.20467601758 | 1864.34224548219 | 0.926080559764552 |
| "ENSDART00000076996" | 21.9131250529994 | 14.5642166637979 | 0.926072359976075 |
| "ENSDART00000134695" | 163.617975299961 | 131.971995691482 | 0.926054571906241 |
| "ENSDART00000139889" | 300.29944725128 | 214.97476344376 | 0.926041298958534 |
| "ENSDART00000076494" | 1081.75612464846 | 941.260209677229 | 0.926027357485259 |
| "ENSDART00000108492" | 99.3714289424517 | 78.4574650180356 | 0.926021886686207 |
| "ENSDART00000148685" | 148.033148091237 | 120.700973949214 | 0.925998893251836 |
| "ENSDART00000122904" | 1629.98443914737 | 1425.04323808006 | 0.925963966825739 |
| "ENSDART00000103004" | 1268.67288942163 | 1074.32975759044 | 0.925961213664683 |
| "ENSDART00000102041" | 16.6831100362075 | 10.5480364944321 | 0.925952049550208 |
| "ENSDART00000147549" | 105.374309091397 | 85.259463019464 | 0.925921539360129 |
| "ENSDART00000150204" | 497.169731904353 | 431.713179668799 | 0.925895490618549 |
| "ENSDART00000132695" | 27.8235651059508 | 17.6514726313899 | 0.92588893057705 |
| "ENSDART00000124453" | 648.286017111609 | 547.604187774557 | 0.925861316837016 |
| "ENSDART00000137696" | 199.552380134673 | 156.398943731428 | 0.925854720249683 |
| "ENSDART00000141287" | 28.6872799872516 | 20.4781444087163 | 0.925832385436789 |
| "ENSDART00000102505" | 70.5865001104437 | 49.4935733866172 | 0.925831774353369 |
| "ENSDART00000156276" | 88.3011579585216 | 68.4771859912901 | 0.925804998705549 |
| "ENSDART00000033037" | 3072.95907047315 | 2666.18086954167 | 0.925800177335337 |
| "ENSDART00000055292" | 238.989656129426 | 192.020292236513 | 0.92577104101975 |
| "ENSDART00000048537" | 51.270262989819 | 39.0850605835836 | 0.925738150665085 |
| "ENSDART00000052660" | 91.2729489937744 | 69.1734775043904 | 0.925659077675775 |
| "ENSDART00000057155" | 25.7633840127003 | 16.3890961293906 | 0.925641589495123 |
| "ENSDART00000052039" | 69.5450890834064 | 52.3599040170493 | 0.92563478553876 |
| "ENSDART00000086905" | 80.418439016046 | 58.6185107759084 | 0.925614383141434 |
| "ENSDART00000128722" | 362.730158073924 | 302.378151197277 | 0.925613039313726 |
| "ENSDART00000089207" | 352.196578443532 | 301.911516334739 | 0.9256059959493 |
| "ENSDART00000108937" | 638.510547526465 | 585.082275023913 | 0.925594812792006 |
| "ENSDART00000089651" | 45.1693820604046 | 33.8134308950995 | 0.925591108816878 |
| "ENSDART00000065984" | 122.27582628779 | 96.0360695394656 | 0.925589195947775 |
| "ENSDART00000122050" | 168.511587569259 | 135.921589456446 | 0.925589179880864 |
| "ENSDART00000148062" | 7167.58740410644 | 6734.0045087804 | 0.925588236848966 |
| "ENSDART00000057469" | 76.5048610960024 | 62.6922696784148 | 0.925587601803741 |
| "ENSDART00000141594" | 18.9858130231782 | 13.35917857604 | 0.925587593824118 |
| "ENSDART00000082124" | 1288.18621692526 | 1211.36166280601 | 0.925587557736507 |
| "ENSDART00000111744" | 6254.23651833337 | 5885.65544610238 | 0.92558754819087 |
| "ENSDART00000137630" | 2932.68698973162 | 2739.63229004009 | 0.92558734415602 |
| "ENSDART00000093247" | 168.802675380175 | 136.218250474511 | 0.925587168816481 |
| "ENSDART00000088177" | 182.028130148128 | 153.353740052427 | 0.925586538449845 |
| "ENSDART00000132644" | 74.3658380979761 | 58.611106406398 | 0.925586389918613 |
| "ENSDART00000125124" | 12918.8042209422 | 12113.1424336659 | 0.925586032890982 |
| "ENSDART00000057432" | 98.7789391249491 | 81.6321607668781 | 0.925585966494098 |
| "ENSDART00000149909" | 458.743689993848 | 405.415954917094 | 0.92558570876577 |
| "ENSDART00000064142" | 81.9358840758488 | 66.1024656868767 | 0.925585601950931 |
| "ENSDART00000151056" | 254.43344431003 | 218.998285307938 | 0.925585272696594 |
| "ENSDART00000147440" | 45.2436860729124 | 35.6416553182675 | 0.925584830981921 |
| "ENSDART00000133939" | 498.94130422226 | 452.101076148848 | 0.925584414256953 |
| "ENSDART00000134768" | 110.949787154634 | 88.3808830171691 | 0.925583318810937 |
| "ENSDART00000079682" | 161.948891110516 | 130.791083509943 | 0.925583219256312 |
| "ENSDART00000023454" | 705.300965823153 | 637.044938730244 | 0.925583202498407 |
| "ENSDART00000024084" | 2572.0374234796 | 2384.25809367769 | 0.925583037743635 |
| "ENSDART00000139267" | 52.4828899877651 | 40.6049166371724 | 0.925582377392695 |
| "ENSDART00000078428" | 67.4766351218362 | 52.0840271987955 | 0.925582305595752 |
| "ENSDART00000029553" | 17.7543130553903 | 12.0817497649517 | 0.925582266143289 |
| "ENSDART00000156901" | 18.9303489655817 | 13.0207088394516 | 0.925582015765427 |
| "ENSDART00000148984" | 1191.88846862071 | 1096.27102209843 | 0.925581932971064 |
| "ENSDART00000062067" | 311.894316141413 | 269.123953987569 | 0.925581808516176 |
| "ENSDART00000115106" | 272.925054202026 | 236.405724143907 | 0.92558179660509 |
| "ENSDART00000109752" | 705.070012021824 | 631.275473182544 | 0.925581625476594 |
| "ENSDART00000110041" | 56.7529820362797 | 44.0050810240772 | 0.925580587216194 |
| "ENSDART00000073806" | 262.885204935906 | 213.338966292494 | 0.92558035669215 |
| "ENSDART00000155827" | 231.722668534848 | 197.335475550513 | 0.925579716469126 |
| "ENSDART00000135435" | 406.369719808856 | 355.863610956347 | 0.925578619987493 |
| "ENSDART00000148522" | 66.3578889776643 | 53.3593061444666 | 0.925578264668968 |
| "ENSDART00000149236" | 395.626758258539 | 359.828006957994 | 0.925577533506586 |
| "ENSDART00000081980" | 279.364060285504 | 242.477023032054 | 0.92557714803055 |
| "ENSDART00000130319" | 45.7272230727118 | 34.7065185879725 | 0.925576843270348 |
| "ENSDART00000026679" | 150.893692090515 | 122.896034322494 | 0.925575687217564 |
| "ENSDART00000123304" | 63.0798391227749 | 49.2776654858203 | 0.925575567016132 |
| "ENSDART00000027393" | 2277.83654949763 | 2145.72557814749 | 0.925575283436061 |
| "ENSDART00000124144" | 248.280834299071 | 216.947525743521 | 0.925575210048358 |
| "ENSDART00000006636" | 117.153774311426 | 91.9300594045329 | 0.92557461758743 |
| "ENSDART00000155489" | 19.2351340125077 | 13.3343317131242 | 0.925574588463806 |
| "ENSDART00000102431" | 49.2093450102133 | 37.8974617380622 | 0.925573814791818 |
| "ENSDART00000145980" | 3982.92721152931 | 3718.55818545269 | 0.925572718595383 |
| "ENSDART00000042755" | 16957.8425153468 | 14702.9183789966 | 0.925571470786501 |
| "ENSDART00000137490" | 43.0156720490923 | 33.8256156332427 | 0.925566587962318 |
| "ENSDART00000134150" | 80.034526158493 | 63.3371949868957 | 0.925566012588825 |
| "ENSDART00000151235" | 284.68792314114 | 242.189376187588 | 0.925564820989268 |
| "ENSDART00000111127" | 101.347944332807 | 77.7879299143691 | 0.925563737727512 |
| "ENSDART00000007012" | 93.7393649846202 | 73.0549143509904 | 0.925562242313628 |
| "ENSDART00000017975" | 3359.9968324881 | 3103.51825925023 | 0.925561541708534 |
| "ENSDART00000060978" | 69.8200821381869 | 55.8384280857376 | 0.925560412759646 |
| "ENSDART00000149009" | 19.0043010423771 | 13.9446156048624 | 0.925558255861 |
| "ENSDART00000039788" | 804.088008484707 | 706.450742678541 | 0.92555782997138 |
| "ENSDART00000122718" | 602.466353942903 | 544.436030306016 | 0.925557125461156 |
| "ENSDART00000130302" | 444.061731348958 | 404.586958228312 | 0.925553966130271 |
| "ENSDART00000011251" | 25455.0434498832 | 23632.8441259127 | 0.92555368500126 |
| "ENSDART00000129798" | 659.104202164786 | 563.793126234654 | 0.925553502233423 |
| "ENSDART00000014703" | 208.532293082564 | 182.316436591687 | 0.925552693398426 |
| "ENSDART00000024778" | 376.118137978892 | 332.618108656931 | 0.925552570915721 |
| "ENSDART00000144154" | 42.9861989777285 | 31.0295258607238 | 0.925551799134347 |
| "ENSDART00000146590" | 52.5715620927772 | 40.6142338043697 | 0.925549617318012 |
| "ENSDART00000132838" | 36.8487269605594 | 25.5135561320524 | 0.925549029698604 |
| "ENSDART00000123243" | 51.4260550610866 | 39.0470742092656 | 0.925549011689281 |
| "ENSDART00000134190" | 477.81770661219 | 439.21583158886 | 0.92554759880965 |
| "ENSDART00000156885" | 181.119936261395 | 152.201737396907 | 0.925544734267409 |
| "ENSDART00000131824" | 101.359633256397 | 80.5725526542735 | 0.925543670227903 |
| "ENSDART00000063870" | 12778.1191655974 | 11960.7828639208 | 0.925538134141118 |
| "ENSDART00000014661" | 145.994519136743 | 117.694164854125 | 0.925536525491145 |
| "ENSDART00000150953" | 520.019736484818 | 482.883634717338 | 0.925532033650082 |
| "ENSDART00000086512" | 408.802074291794 | 372.875715776947 | 0.925531453014494 |
| "ENSDART00000129107" | 26.2691770226808 | 20.141824537545 | 0.925531258509667 |
| "ENSDART00000002571" | 1156.64111858605 | 1030.70660446598 | 0.925530943440506 |
| "ENSDART00000082011" | 15.663603083639 | 9.29927689046447 | 0.925529887418543 |
| "ENSDART00000064616" | 29.3643219830717 | 21.0437520109859 | 0.92552986108988 |
| "ENSDART00000149325" | 36.4315730406602 | 27.5815805456742 | 0.925529306517554 |
| "ENSDART00000075351" | 847.046472283227 | 733.430559863603 | 0.925528072344494 |
| "ENSDART00000050687" | 111.621620401692 | 80.9611935033231 | 0.925527317162285 |
| "ENSDART00000029353" | 16.5865500135187 | 12.1087464932847 | 0.925525299243836 |
| "ENSDART00000048642" | 399.904403818363 | 344.417940216971 | 0.925525268808273 |
| "ENSDART00000139803" | 57.2961030203703 | 41.4824746343268 | 0.925525078609531 |
| "ENSDART00000109244" | 308.949754503527 | 271.83098767241 | 0.925523865792954 |
| "ENSDART00000123929" | 21.1285709972562 | 15.1888351590965 | 0.925523850391681 |
| "ENSDART00000155189" | 74.1912060717973 | 59.2603314457132 | 0.925522515615672 |
| "ENSDART00000079523" | 695.887634867513 | 600.831858486103 | 0.925521311320484 |
| "ENSDART00000089113" | 173.787588379123 | 139.205098291314 | 0.925520898890427 |
| "ENSDART00000151411" | 57.0879110110637 | 46.1951866356921 | 0.925519737482716 |
| "ENSDART00000146957" | 166.202606754106 | 135.784452698195 | 0.925519275787609 |
| "ENSDART00000108875" | 32.9649080177307 | 22.3087557650318 | 0.925518072297673 |
| "ENSDART00000018908" | 453.623012745402 | 409.001030488057 | 0.925513283327267 |
| "ENSDART00000056850" | 56.7974940566419 | 46.5083321227352 | 0.925513280881886 |
| "ENSDART00000133577" | 29.7803870809032 | 21.694889847988 | 0.925512506844391 |
| "ENSDART00000149528" | 677.630850069682 | 571.296048010236 | 0.925510968607305 |
| "ENSDART00000126052" | 145.440380085557 | 121.006474747848 | 0.925508125312992 |
| "ENSDART00000124448" | 986.108838012446 | 918.457839203559 | 0.925507067954806 |
| "ENSDART00000074099" | 20.5891849892172 | 14.2611060494808 | 0.9255063064878 |
| "ENSDART00000135156" | 429.745831126923 | 384.825652464777 | 0.925502934125819 |
| "ENSDART00000124198" | 33.106331996494 | 23.8455769253964 | 0.925502081321552 |
| "ENSDART00000039235" | 125.88615896348 | 104.304473450313 | 0.925500617175749 |
| "ENSDART00000065467" | 353.418430815081 | 302.573890638963 | 0.925498951240236 |
| "ENSDART00000132320" | 25.4171510649696 | 16.7067816661673 | 0.925498827542832 |
| "ENSDART00000149538" | 61.5552430316505 | 48.3559085858266 | 0.925498753960228 |
| "ENSDART00000156454" | 27.4222200363359 | 18.9697553877421 | 0.925494061813952 |
| "ENSDART00000126937" | 424.457657398034 | 373.016846021266 | 0.925490591768921 |
| "ENSDART00000146338" | 499.125264923895 | 425.469065650688 | 0.925490103610745 |
| "ENSDART00000134053" | 1136.61121536692 | 1025.03829403512 | 0.92548970071525 |
| "ENSDART00000080196" | 99.1836672349033 | 79.1246099373857 | 0.925488294525411 |
| "ENSDART00000099622" | 359.134057173954 | 305.622679594439 | 0.925485564841883 |
| "ENSDART00000083062" | 514.790189977875 | 444.801153328053 | 0.925482494577023 |
| "ENSDART00000015391" | 255.820351398443 | 219.788709768608 | 0.925477916726981 |
| "ENSDART00000063750" | 33.3368130309124 | 26.0676967017074 | 0.925477532883934 |
| "ENSDART00000111709" | 53.6804780367129 | 41.5223738063317 | 0.925477441999282 |
| "ENSDART00000131628" | 16.9064070243737 | 10.577900793711 | 0.925474932595847 |
| "ENSDART00000123365" | 50.4502902275944 | 32.8405481093856 | 0.925473546610952 |
| "ENSDART00000136886" | 46.2929850176264 | 36.9360459849628 | 0.925472978178405 |
| "ENSDART00000153778" | 54.5208150708345 | 40.5537907514522 | 0.925472242720651 |
| "ENSDART00000150386" | 64.330179045474 | 48.8899794100296 | 0.925472152425238 |
| "ENSDART00000139951" | 18.647116057412 | 12.7092358311961 | 0.925471132072147 |
| "ENSDART00000154023" | 18.997116996125 | 13.6524946365303 | 0.925470069683284 |
| "ENSDART00000146281" | 108.074204206358 | 86.1969866829064 | 0.925469546229002 |
| "ENSDART00000149041" | 31.7933769848768 | 22.9854581704785 | 0.925468586328535 |
| "ENSDART00000139156" | 56.9016230762258 | 44.0203704008965 | 0.925468294381767 |
| "ENSDART00000063789" | 7336.68885065526 | 6837.06371306712 | 0.925465390654343 |
| "ENSDART00000122217" | 58.768651109168 | 45.6455861157706 | 0.925464876820323 |
| "ENSDART00000128167" | 1547.04367318989 | 1362.75762431711 | 0.925464450508786 |
| "ENSDART00000102433" | 273.271320164687 | 238.935017197639 | 0.925463484143317 |
| "ENSDART00000148371" | 63.4264570211485 | 51.1706326927402 | 0.925463338603421 |
| "ENSDART00000082143" | 184.385978271913 | 158.134780114019 | 0.925461109127486 |
| "ENSDART00000126782" | 34.4936240355496 | 26.6990051121567 | 0.925455629099392 |
| "ENSDART00000101226" | 60.9261291114626 | 47.7255549486364 | 0.92545236518945 |
| "ENSDART00000021753" | 402.328199527131 | 361.018236306731 | 0.92544509287154 |
| "ENSDART00000123842" | 97.6001910309125 | 76.4701277958153 | 0.925443452886043 |
| "ENSDART00000139930" | 21.6638040636699 | 15.5065206958732 | 0.925443396024567 |
| "ENSDART00000052342" | 120.540359018445 | 99.8511537982244 | 0.925441595069915 |
| "ENSDART00000104673" | 579.371751082677 | 512.877072384481 | 0.925441194221573 |
| "ENSDART00000104933" | 335.900093448723 | 289.279938735643 | 0.925437672339585 |
| "ENSDART00000078040" | 38.850060955874 | 27.8787189503693 | 0.925436045109082 |
| "ENSDART00000098529" | 202.214111344376 | 165.059814086699 | 0.925430784328735 |
| "ENSDART00000142722" | 198.071108197051 | 160.106762033553 | 0.925429734375704 |
| "ENSDART00000078181" | 220.92714197184 | 180.851532019193 | 0.925428526666036 |
| "ENSDART00000147161" | 19.5700629872917 | 13.3436488803215 | 0.92542807959722 |
| "ENSDART00000132420" | 41.977292126682 | 30.7084953663718 | 0.925425617863185 |
| "ENSDART00000131355" | 74.0881988892116 | 57.0912503528092 | 0.925420796369203 |
| "ENSDART00000135456" | 36.6805420942774 | 27.5908977128715 | 0.925419860650052 |
| "ENSDART00000146738" | 15.0393459765009 | 10.228918797767 | 0.925418201253528 |
| "ENSDART00000125918" | 737.530050776989 | 665.463937782168 | 0.925415571204865 |
| "ENSDART00000111670" | 1095.38910752648 | 982.787509334991 | 0.925415251184276 |
| "ENSDART00000123520" | 3245.59032708491 | 3005.64328513755 | 0.925412829859025 |
| "ENSDART00000044738" | 192.057023119951 | 163.580986371407 | 0.925409290378673 |
| "ENSDART00000124504" | 295.210937728698 | 250.177129413887 | 0.925408022553138 |
| "ENSDART00000038929" | 107.581155927104 | 87.2444100573678 | 0.925405412091013 |
| "ENSDART00000137220" | 80.9129610680104 | 65.7169327276721 | 0.925403367725568 |
| "ENSDART00000045069" | 1552.18356002785 | 1413.25468462605 | 0.925402170706963 |
| "ENSDART00000125162" | 146.320823307495 | 122.877465913967 | 0.925399432582733 |
| "ENSDART00000109681" | 29.594418066847 | 22.3233306874914 | 0.925395913280404 |
| "ENSDART00000154388" | 145.380092229841 | 122.886065375635 | 0.925393793352583 |
| "ENSDART00000081553" | 2241.68152684367 | 1749.50650259052 | 0.925388184637767 |
| "ENSDART00000017728" | 840.21645285515 | 761.673105938183 | 0.92538739014234 |
| "ENSDART00000060930" | 190.449817133069 | 162.845342249967 | 0.925384758736339 |
| "ENSDART00000097176" | 25.1274379819725 | 17.6412006909336 | 0.925380297024946 |
| "ENSDART00000127600" | 108.713918228068 | 85.2346129053792 | 0.925376470984285 |
| "ENSDART00000149774" | 21.9874290655072 | 16.1442787025739 | 0.925375466416989 |
| "ENSDART00000078824" | 481.017618433728 | 440.271136719462 | 0.925372918671646 |
| "ENSDART00000133597" | 40.2332000533044 | 30.3614261681147 | 0.925371409415796 |
| "ENSDART00000103373" | 12634.4513896372 | 11253.4911447461 | 0.925366664314202 |
| "ENSDART00000091159" | 31.9680090110556 | 24.8024526287623 | 0.925364583881377 |
| "ENSDART00000154045" | 670.701501589974 | 612.266980927164 | 0.925361668225041 |
| "ENSDART00000012637" | 575.172982486318 | 509.991663171101 | 0.925360547920046 |
| "ENSDART00000053864" | 880.076579771061 | 801.327846540647 | 0.925359521251837 |
| "ENSDART00000067764" | 281.415935495504 | 240.312416063016 | 0.92535888325717 |
| "ENSDART00000079645" | 80.7564321103875 | 65.1429627314643 | 0.925357897323016 |
| "ENSDART00000125363" | 205.442858395715 | 174.799035356135 | 0.925355102503423 |
| "ENSDART00000139146" | 3326.38286628762 | 3063.51468150823 | 0.925349225688829 |
| "ENSDART00000112969" | 197.903759261917 | 171.417745622524 | 0.925348757356445 |
| "ENSDART00000142582" | 66890.0684345082 | 53906.5789301892 | 0.925348366453663 |
| "ENSDART00000008174" | 1369.11982595772 | 1093.94260089225 | 0.925346214369442 |
| "ENSDART00000097770" | 317.77305493459 | 270.25013875107 | 0.925345027698892 |
| "ENSDART00000129535" | 17.0029670470625 | 11.4583263618113 | 0.925343952452888 |
| "ENSDART00000153465" | 43.9344660373468 | 34.1382837336563 | 0.925338846994797 |
| "ENSDART00000016891" | 677.405828631187 | 606.185571486918 | 0.925338050562498 |
| "ENSDART00000130471" | 158.287632269498 | 128.238211113658 | 0.92532541363568 |
| "ENSDART00000037027" | 15.7163879723209 | 9.89761636295872 | 0.925325349079349 |
| "ENSDART00000062366" | 283.58197227197 | 245.398953672072 | 0.925320476066437 |
| "ENSDART00000101545" | 152.928586068957 | 126.653780167012 | 0.925319808756009 |
| "ENSDART00000098696" | 27.3739400249915 | 21.0994179464396 | 0.925317484294261 |
| "ENSDART00000080749" | 155.88868818062 | 123.379999960103 | 0.925315046526068 |
| "ENSDART00000105315" | 216.535385389826 | 186.073705049575 | 0.925314590109993 |
| "ENSDART00000143556" | 159.843109174836 | 130.288786531536 | 0.925313680975944 |
| "ENSDART00000127239" | 34.3710400116975 | 22.8944331127534 | 0.925313039679067 |
| "ENSDART00000098111" | 2337.49197203165 | 1876.14795641415 | 0.925310601946222 |
| "ENSDART00000144283" | 283.221085325883 | 246.974475661115 | 0.925309527948022 |
| "ENSDART00000149989" | 178.958371347337 | 140.799488431312 | 0.925309125837834 |
| "ENSDART00000150187" | 51.9050871835485 | 37.2155048285223 | 0.925305720178084 |
| "ENSDART00000036419" | 27.7567970754075 | 18.8550785592594 | 0.925303870392657 |
| "ENSDART00000151784" | 921.301650642517 | 853.261050906123 | 0.925303434058699 |
| "ENSDART00000156082" | 21.9764770282728 | 16.7380781253346 | 0.925299225669345 |
| "ENSDART00000108791" | 188.811092490409 | 158.362635685229 | 0.925289628650306 |
| "ENSDART00000010140" | 1253.14992714319 | 1180.20885090163 | 0.925289480029142 |
| "ENSDART00000109803" | 564.433300674488 | 512.753934448402 | 0.925289464142403 |
| "ENSDART00000124830" | 118.984563192326 | 82.4633699957762 | 0.925288246713576 |
| "ENSDART00000065143" | 858.112759657247 | 741.533101636719 | 0.925287072010764 |
| "ENSDART00000126192" | 160.992351182578 | 115.351465216745 | 0.9252818108757 |
| "ENSDART00000077840" | 692.98510842772 | 598.323286957485 | 0.925279004035995 |
| "ENSDART00000136428" | 386.847116387347 | 350.779997090597 | 0.925277333083557 |
| "ENSDART00000017349" | 73.9343491005033 | 59.2373973804842 | 0.925277163792213 |
| "ENSDART00000098446" | 51.7907760280161 | 41.5813846993607 | 0.925273519371088 |
| "ENSDART00000006513" | 1856.7329001304 | 1726.87996935349 | 0.925272607569792 |
| "ENSDART00000125723" | 4888.49272768743 | 4044.51195894643 | 0.925271336950838 |
| "ENSDART00000136874" | 20.1984400211244 | 14.8790346296287 | 0.925270526798696 |
| "ENSDART00000041269" | 4151.37153021389 | 3419.97182838579 | 0.925270507072085 |
| "ENSDART00000141601" | 156.825651267292 | 130.935868207772 | 0.925267737319641 |
| "ENSDART00000122015" | 141.93188221118 | 119.349007800548 | 0.925266447799782 |
| "ENSDART00000141994" | 189.947440178358 | 159.495348975523 | 0.925265774815566 |
| "ENSDART00000054059" | 74.739920755293 | 55.4939861395271 | 0.925260960489775 |
| "ENSDART00000148160" | 561.891073883493 | 504.832115846844 | 0.925257004021503 |
| "ENSDART00000129409" | 17.2745440465731 | 12.0757775553297 | 0.925255943748219 |
| "ENSDART00000040707" | 514.797290464921 | 475.239200773297 | 0.925254603510693 |
| "ENSDART00000101952" | 60.2313029678683 | 45.0252673513064 | 0.925253083580697 |
| "ENSDART00000142061" | 99.1542601934007 | 81.5738675793777 | 0.925248360558461 |
| "ENSDART00000133943" | 96.0009389915681 | 76.0299463491143 | 0.925246904398073 |
| "ENSDART00000136667" | 1666.13456278859 | 1515.45660459072 | 0.925243481863846 |
| "ENSDART00000144744" | 3388.87957982813 | 3036.9100037959 | 0.925239504417038 |
| "ENSDART00000145105" | 22.010388947113 | 14.2637333015275 | 0.925233928793323 |
| "ENSDART00000133005" | 59.103580083952 | 47.1253059296242 | 0.925233260513152 |
| "ENSDART00000052989" | 2497.41291171713 | 2312.86655228165 | 0.925230194580829 |
| "ENSDART00000039807" | 64.2774271717227 | 48.7208976641186 | 0.925226512698527 |
| "ENSDART00000153482" | 52.0544320949193 | 38.820413730214 | 0.925226354574547 |
| "ENSDART00000049075" | 491.411096055697 | 439.671302412381 | 0.925225678801625 |
| "ENSDART00000112669" | 1251.55874766928 | 1010.29967257066 | 0.925218131907768 |
| "ENSDART00000153543" | 131.631478269707 | 104.929091945612 | 0.925215244089039 |
| "ENSDART00000088513" | 599.708667537144 | 553.011363924217 | 0.925213027434219 |
| "ENSDART00000134805" | 8175.75946052969 | 6746.74911914078 | 0.925211162759307 |
| "ENSDART00000102855" | 462.159010885876 | 404.899632831063 | 0.925208223746647 |
| "ENSDART00000142593" | 14.6116249700103 | 9.32961857637282 | 0.925208081436681 |
| "ENSDART00000085686" | 500.38596958654 | 455.943641024908 | 0.925207338045649 |
| "ENSDART00000080120" | 2113.88022491056 | 1960.47015622247 | 0.925204016166294 |
| "ENSDART00000047662" | 1117.65518803507 | 1013.55544005983 | 0.925201510392161 |
| "ENSDART00000154616" | 22.4117340167278 | 15.1988700318225 | 0.925198282076026 |
| "ENSDART00000021491" | 1989.47351643334 | 1881.75071477079 | 0.925198110043952 |
| "ENSDART00000012673" | 454.108056532843 | 393.017885849387 | 0.925194863695982 |
| "ENSDART00000133338" | 825.495760537058 | 702.56395261419 | 0.925192842070194 |
| "ENSDART00000140696" | 2037.19748710858 | 1894.93183671745 | 0.925189474052182 |
| "ENSDART00000148232" | 6030.04232432999 | 5555.28389269809 | 0.925188463891073 |
| "ENSDART00000150863" | 351.424868163307 | 315.173463826871 | 0.925185949797411 |
| "ENSDART00000153971" | 47.2333311446373 | 32.9631066940083 | 0.925184185905939 |
| "ENSDART00000149501" | 31.3354790355978 | 23.2479551584309 | 0.925182184712575 |
| "ENSDART00000129174" | 142.184619255779 | 119.372899890204 | 0.92518181343206 |
| "ENSDART00000139513" | 16.8245670299014 | 12.4109023343428 | 0.925181448220542 |
| "ENSDART00000140151" | 64.6839809900998 | 49.5743265038859 | 0.925180822530314 |
| "ENSDART00000125852" | 376.493509591619 | 319.341184535084 | 0.925180344773786 |
| "ENSDART00000131339" | 434.933326950187 | 380.681649453188 | 0.925179311739709 |
| "ENSDART00000062551" | 130.277361263136 | 106.375125115982 | 0.925179004554024 |
| "ENSDART00000145755" | 48.806592197749 | 34.0357916433168 | 0.925177590319821 |
| "ENSDART00000040769" | 232.64938345571 | 194.448594536831 | 0.925173995222007 |
| "ENSDART00000139737" | 34.2823679066854 | 23.5223965656273 | 0.925173297149558 |
| "ENSDART00000109546" | 225.017426260344 | 195.63069855936 | 0.925167761584995 |
| "ENSDART00000136321" | 130.124248360783 | 102.852165076724 | 0.925164179622671 |
| "ENSDART00000146671" | 214.181019351172 | 182.790541749602 | 0.925160206312342 |
| "ENSDART00000140652" | 50.1092990435565 | 38.756385400822 | 0.925157002963144 |
| "ENSDART00000129794" | 779.009686445226 | 692.760913447355 | 0.925154890059283 |
| "ENSDART00000059321" | 1286.15215870842 | 1105.94374980117 | 0.92515438788672 |
| "ENSDART00000111658" | 68.1801190843932 | 52.0897623406872 | 0.925153937930163 |
| "ENSDART00000108477" | 3271.22243845317 | 2921.38997358418 | 0.925151866092262 |
| "ENSDART00000065373" | 3448.66300157646 | 3192.61877605532 | 0.925150501839398 |
| "ENSDART00000043016" | 25.2722780160058 | 17.7188459183573 | 0.925147631392524 |
| "ENSDART00000138437" | 26.1798010462442 | 19.2045379418328 | 0.925147039190864 |
| "ENSDART00000076318" | 65.7585670496219 | 53.9812973877186 | 0.925146556170971 |
| "ENSDART00000027454" | 135.47040024153 | 113.506753073431 | 0.925142237833913 |
| "ENSDART00000133443" | 71.9440001573537 | 57.9826655668946 | 0.92514103906717 |
| "ENSDART00000081319" | 79.2171490059772 | 61.3790043584256 | 0.925139038319586 |
| "ENSDART00000104762" | 657.027829483911 | 606.615184501903 | 0.92513671263916 |
| "ENSDART00000079689" | 47.70556417149 | 35.6719970041759 | 0.925131475848961 |
| "ENSDART00000148580" | 191.635749273357 | 162.202326488004 | 0.925129447974834 |
| "ENSDART00000112088" | 107.193475078569 | 88.7014328737227 | 0.92512768889559 |
| "ENSDART00000148809" | 334.910092582448 | 296.96569750924 | 0.925124919161315 |
| "ENSDART00000002962" | 1810.25847276246 | 1627.89917996519 | 0.925122063287307 |
| "ENSDART00000139310" | 508.114673665655 | 446.727267117129 | 0.925121015180377 |
| "ENSDART00000134304" | 443.30872470686 | 389.015382001582 | 0.925118487291372 |
| "ENSDART00000073518" | 118.448593239557 | 95.7059621968155 | 0.925115979657877 |
| "ENSDART00000046821" | 31.9484321697892 | 15.2022149893979 | 0.925113792316922 |
| "ENSDART00000144567" | 25.3839430175542 | 17.6939990554415 | 0.925110916180698 |
| "ENSDART00000138701" | 89.121599230595 | 67.5547113857678 | 0.925109451402179 |
| "ENSDART00000150246" | 29.0780249553445 | 19.8291564371314 | 0.925107531342389 |
| "ENSDART00000066257" | 284.299439376388 | 249.122058099847 | 0.925105416608099 |
| "ENSDART00000057696" | 344.266382405928 | 313.243942405521 | 0.925104797634452 |
| "ENSDART00000122734" | 2967.48585558678 | 2427.68126685124 | 0.925101173090495 |
| "ENSDART00000151762" | 14.7640009860081 | 10.5370468484471 | 0.9250994955937 |
| "ENSDART00000084528" | 275.042206140514 | 244.292585330449 | 0.925099061129569 |
| "ENSDART00000032151" | 2498.03990279452 | 2361.78190886242 | 0.925088222935624 |
| "ENSDART00000015267" | 46.0960969812665 | 33.8664695785065 | 0.925086397693782 |
| "ENSDART00000108654" | 356.551106096227 | 316.743734562989 | 0.92508217079612 |
| "ENSDART00000060550" | 552.519496477156 | 502.850813508944 | 0.925081669476069 |
| "ENSDART00000065183" | 118.314705242758 | 95.1305567895502 | 0.925080696492663 |
| "ENSDART00000007333" | 654.028540674785 | 584.205833188374 | 0.925080233574745 |
| "ENSDART00000149635" | 111.657072123104 | 90.5599989827991 | 0.925077892011588 |
| "ENSDART00000133107" | 53.7992940695829 | 40.8542741137226 | 0.925067840041031 |
| "ENSDART00000151852" | 21.046346052141 | 13.0472281811551 | 0.925050067165749 |
| "ENSDART00000152765" | 585.759564668079 | 502.376951921097 | 0.925049745718289 |
| "ENSDART00000088569" | 126.492983858556 | 101.650946082002 | 0.925048897250782 |
| "ENSDART00000143240" | 74.5819840548207 | 58.33809715909 | 0.925042635258807 |
| "ENSDART00000086936" | 151.400606937494 | 121.969737372767 | 0.925042396086476 |
| "ENSDART00000142602" | 30.3238600007061 | 22.0080353350311 | 0.925039513144141 |
| "ENSDART00000099131" | 1205.39097751344 | 1089.45836733152 | 0.925035223796971 |
| "ENSDART00000048003" | 171.415053242052 | 131.186417525312 | 0.925030832769962 |
| "ENSDART00000080286" | 161.67168739667 | 126.33042541421 | 0.925029385816778 |
| "ENSDART00000006948" | 9079.30661909924 | 8437.85875222223 | 0.925028450320686 |
| "ENSDART00000135816" | 468.290937704156 | 386.465950025362 | 0.92502529398928 |
| "ENSDART00000150908" | 14.7451610310969 | 10.8650043256801 | 0.925024855410107 |
| "ENSDART00000057957" | 175.545129054652 | 150.620006130513 | 0.925022933641275 |
| "ENSDART00000012670" | 885.657783593425 | 829.208879995958 | 0.925018173984806 |
| "ENSDART00000046857" | 135.080425174723 | 107.743272740029 | 0.925013770314236 |
| "ENSDART00000079655" | 346.031426048491 | 295.633633215331 | 0.925013330139698 |
| "ENSDART00000127464" | 230.110137225067 | 201.107552747422 | 0.925011975016987 |
| "ENSDART00000008401" | 456.979871490137 | 420.315121966873 | 0.925011845025828 |
| "ENSDART00000059440" | 101.848027069246 | 83.7926423981134 | 0.925010524915654 |
| "ENSDART00000100060" | 851.143814859081 | 725.72229861894 | 0.925010110071845 |
| "ENSDART00000128441" | 172.761568236797 | 144.94444621616 | 0.925009204559063 |
| "ENSDART00000102791" | 1636.40037711968 | 1444.2594000131 | 0.925006776003558 |
| "ENSDART00000130859" | 30.2529720434682 | 23.8823682075563 | 0.925005811989218 |
| "ENSDART00000128721" | 3327.64324811151 | 2956.32778096052 | 0.925005400447338 |
| "ENSDART00000003752" | 2983.62789991671 | 2823.51994773082 | 0.92500108939108 |
| "ENSDART00000154295" | 1298.3125781816 | 1190.31098056097 | 0.924999789166487 |
| "ENSDART00000110511" | 101.491578970636 | 82.900272410769 | 0.924997146497908 |
| "ENSDART00000129224" | 17.8848180118499 | 12.0767323285887 | 0.924991331997375 |
| "ENSDART00000125939" | 438.229981398412 | 368.106547706394 | 0.924991116803635 |
| "ENSDART00000154123" | 204.883257704846 | 168.344821007323 | 0.924989438995556 |
| "ENSDART00000015755" | 186.953828627842 | 148.935605129578 | 0.924987348405335 |
| "ENSDART00000041349" | 10251.7040807212 | 8045.00436078771 | 0.924986638558574 |
| "ENSDART00000156577" | 21.4521959990934 | 15.7986416642052 | 0.924982632421373 |
| "ENSDART00000112468" | 83.6573682033331 | 66.9468176783459 | 0.924979475727046 |
| "ENSDART00000017155" | 250.293054301759 | 184.878217945576 | 0.92497688630645 |
| "ENSDART00000104063" | 485.910344227603 | 436.958115622548 | 0.924975312790439 |
| "ENSDART00000139569" | 43.6745779614258 | 32.5321797398063 | 0.924973692103811 |
| "ENSDART00000140580" | 1080.75100331774 | 938.636974951191 | 0.924970731981985 |
| "ENSDART00000102515" | 156.664148922483 | 131.768032209929 | 0.924968822606974 |
| "ENSDART00000113727" | 51.3294950383978 | 42.186651154736 | 0.924967950136795 |
| "ENSDART00000084689" | 31.019390015725 | 23.2417426299097 | 0.924963954223405 |
| "ENSDART00000010246" | 661.816428301961 | 566.788086372989 | 0.924962051044075 |
| "ENSDART00000101125" | 13097.4555348108 | 12113.0213202458 | 0.924961366235629 |
| "ENSDART00000040838" | 18.5471399794533 | 13.0195137472934 | 0.92496133656544 |
| "ENSDART00000141628" | 15.3516670053914 | 9.61481256182395 | 0.924958906823731 |
| "ENSDART00000157042" | 28.5714950590087 | 20.5034686582617 | 0.924958840967391 |
| "ENSDART00000129911" | 1135.36809250541 | 996.484877412205 | 0.924956749972552 |
| "ENSDART00000145867" | 302.124640389016 | 263.049303639509 | 0.924955782697933 |
| "ENSDART00000103784" | 27.6417490335198 | 21.0846059562497 | 0.924954785654955 |
| "ENSDART00000132433" | 207.082001003948 | 174.321759633676 | 0.924954688239736 |
| "ENSDART00000077887" | 401.549993586492 | 351.934212286233 | 0.924954170176747 |
| "ENSDART00000081367" | 554.928171721478 | 485.804623588774 | 0.924951102618296 |
| "ENSDART00000110121" | 236.946555312525 | 209.030971399563 | 0.924950605916831 |
| "ENSDART00000092347" | 711.374865989059 | 605.264406943056 | 0.924949528614199 |
| "ENSDART00000008140" | 264.704056259357 | 212.928583219205 | 0.924948990377085 |
| "ENSDART00000139939" | 5327.92465463637 | 4843.90934327381 | 0.924945183011233 |
| "ENSDART00000150920" | 502.401286723716 | 421.816442399725 | 0.924944992887599 |
| "ENSDART00000126816" | 122.116717206045 | 99.9166142875049 | 0.924944615072147 |
| "ENSDART00000009827" | 96.9341010723268 | 77.4836242078938 | 0.924943147191195 |
| "ENSDART00000016105" | 637.13012061288 | 589.282888990707 | 0.924934290133581 |
| "ENSDART00000123094" | 253.726192356491 | 218.616634116467 | 0.924932691858525 |
| "ENSDART00000109315" | 191.669694207128 | 166.65110609036 | 0.924932274349046 |
| "ENSDART00000087126" | 2186.84975474444 | 1995.16069338716 | 0.924929701897239 |
| "ENSDART00000058032" | 156.614780089071 | 132.885456123405 | 0.92492795757098 |
| "ENSDART00000027603" | 5545.65872863437 | 4822.12839923724 | 0.92492748277942 |
| "ENSDART00000085304" | 336.497858044849 | 301.428271653828 | 0.924927339571763 |
| "ENSDART00000098108" | 35.9631080047897 | 26.3817969620095 | 0.924923554249197 |
| "ENSDART00000040810" | 225.835122333642 | 190.395382766406 | 0.924915847680905 |
| "ENSDART00000143412" | 99.501197012556 | 82.2276326684265 | 0.924913540370185 |
| "ENSDART00000145503" | 70.3405951763841 | 52.604721443866 | 0.924911701742354 |
| "ENSDART00000098976" | 1316.44147337533 | 1232.95810046858 | 0.924910471926592 |
| "ENSDART00000111499" | 29.4646499967427 | 21.3573748846585 | 0.924908579389823 |
| "ENSDART00000133799" | 61.8434823419371 | 43.0890560148063 | 0.924902209282127 |
| "ENSDART00000150083" | 19.2577419584012 | 12.9975344553234 | 0.924901300825326 |
| "ENSDART00000027465" | 180.875505100046 | 151.848695989028 | 0.924900319282296 |
| "ENSDART00000152390" | 2043.24891138831 | 1812.55879483366 | 0.924899027877574 |
| "ENSDART00000134020" | 6657.69025934432 | 5954.32080162383 | 0.92489799943541 |
| "ENSDART00000135668" | 20.0607840333433 | 15.1955250742472 | 0.924896268790489 |
| "ENSDART00000005778" | 27.4218681006235 | 19.5628371049741 | 0.924896052171275 |
| "ENSDART00000137127" | 227.858778368999 | 197.395134972035 | 0.92489407795726 |
| "ENSDART00000142706" | 41.632148001019 | 31.645781962084 | 0.924893931825373 |
| "ENSDART00000089015" | 227.580369258948 | 197.109292458091 | 0.924891646813607 |
| "ENSDART00000032978" | 18.305003036376 | 12.6958560008947 | 0.924876861197367 |
| "ENSDART00000139769" | 24.1188830666384 | 18.3059554259674 | 0.924873358641022 |
| "ENSDART00000134322" | 71.4117981955669 | 56.1570683957732 | 0.924873224087339 |
| "ENSDART00000098160" | 167.757529377086 | 133.167479544313 | 0.924872503034793 |
| "ENSDART00000114700" | 29.5125780723748 | 22.6293088727543 | 0.924871110879978 |
| "ENSDART00000136490" | 16.6167269563072 | 8.40164914785795 | 0.924870571155087 |
| "ENSDART00000026409" | 6587.4074641424 | 6148.99891190655 | 0.924870498479087 |
| "ENSDART00000078800" | 230.027527329309 | 171.661605024913 | 0.924866216981962 |
| "ENSDART00000048599" | 5702.94265327773 | 5211.27922228209 | 0.924862563567257 |
| "ENSDART00000150817" | 31.2724789960367 | 22.9027955066918 | 0.924861842040713 |
| "ENSDART00000093093" | 56.9762790244459 | 45.272952349069 | 0.92486068787663 |
| "ENSDART00000148548" | 38.8349559770144 | 29.4719205005474 | 0.924859780054652 |
| "ENSDART00000110705" | 46.5984739359772 | 35.0633855912253 | 0.924854440847744 |
| "ENSDART00000014473" | 814.221133777968 | 750.086423175159 | 0.924854257991591 |
| "ENSDART00000021191" | 612.666027390717 | 567.045980636509 | 0.924852008822926 |
| "ENSDART00000073663" | 49.7702171271474 | 34.9812003140681 | 0.924851835255192 |
| "ENSDART00000104848" | 46.4170097992585 | 29.3653689982727 | 0.924848993389648 |
| "ENSDART00000079326" | 91.6573963910374 | 67.026853090086 | 0.924848667450804 |
| "ENSDART00000141827" | 42.8036790338728 | 33.5373170091154 | 0.924847023135431 |
| "ENSDART00000005118" | 58.564579026556 | 44.2885992795717 | 0.924844862743052 |
| "ENSDART00000126646" | 227.320063217454 | 194.262554186482 | 0.924840636305423 |
| "ENSDART00000130288" | 27.2147318984539 | 17.7353303873348 | 0.924838410065392 |
| "ENSDART00000135824" | 1442.74215967783 | 1300.54324547547 | 0.924837473122296 |
| "ENSDART00000110377" | 325.078758503479 | 280.764969239816 | 0.924819004833827 |
| "ENSDART00000005547" | 1544.89820115776 | 1350.79722731445 | 0.924812154028779 |
| "ENSDART00000091873" | 718.616346592839 | 668.849826989042 | 0.924809611418459 |
| "ENSDART00000037879" | 877.024451271915 | 768.014453941086 | 0.924809351798312 |
| "ENSDART00000124728" | 49.2278330294121 | 33.1878576265419 | 0.924808204760325 |
| "ENSDART00000148593" | 448.125221735013 | 372.021088593853 | 0.924808040383385 |
| "ENSDART00000148215" | 194.133547603196 | 159.877540228322 | 0.924806961827795 |
| "ENSDART00000130629" | 15.1691140466052 | 10.5227122448868 | 0.924804817405488 |
| "ENSDART00000145601" | 1231.6926741977 | 1125.17712096326 | 0.924803018737283 |
| "ENSDART00000144632" | 185.431795131496 | 158.396322328712 | 0.924802400489049 |
| "ENSDART00000148206" | 2607.30134783922 | 2420.90271634063 | 0.924802210315201 |
| "ENSDART00000147572" | 431.118068731394 | 382.349091849686 | 0.924800534776302 |
| "ENSDART00000142707" | 192.51492106923 | 162.929917711441 | 0.924799586023006 |
| "ENSDART00000079538" | 164.727562100391 | 136.205348030839 | 0.924798595688461 |
| "ENSDART00000136134" | 155.198014978651 | 127.289628627194 | 0.924796770823894 |
| "ENSDART00000098750" | 260.073864695387 | 219.949254727549 | 0.924790727579116 |
| "ENSDART00000121449" | 150.373948953604 | 123.684545985478 | 0.924789072576715 |
| "ENSDART00000129769" | 312.777823482908 | 271.233062911847 | 0.924788335781786 |
| "ENSDART00000124920" | 1060.56460415591 | 950.094484893816 | 0.924786776122789 |
| "ENSDART00000091798" | 44.0050020588723 | 31.2619182304982 | 0.924784479778452 |
| "ENSDART00000085684" | 25.979145018902 | 17.9691581681666 | 0.92478169631106 |
| "ENSDART00000064144" | 271.213785225421 | 237.347066900385 | 0.924778934286956 |
| "ENSDART00000061327" | 201.253049009756 | 167.480162202672 | 0.924775903104918 |
| "ENSDART00000145825" | 49.0008010651943 | 37.8391685505618 | 0.924774775128866 |
| "ENSDART00000098677" | 130.032193215432 | 108.944794728348 | 0.924770439982205 |
| "ENSDART00000017436" | 163.043054011305 | 135.633284329981 | 0.924769097158737 |
| "ENSDART00000147441" | 156.052082263714 | 126.671456476978 | 0.9247616144173 |
| "ENSDART00000026712" | 1537.53016602295 | 1370.57756946992 | 0.924758406971605 |
| "ENSDART00000140559" | 43.7300420190224 | 34.4413943479734 | 0.924755606982973 |
| "ENSDART00000000102" | 60.077838129803 | 45.6195441606966 | 0.924750207896635 |
| "ENSDART00000011163" | 221.85695402719 | 193.757560778993 | 0.924744657930319 |
| "ENSDART00000088359" | 17.3043360387186 | 11.5161421626822 | 0.924743315486879 |
| "ENSDART00000122453" | 92.247240054556 | 74.7852494082507 | 0.924741235741458 |
| "ENSDART00000073827" | 2263.07514666619 | 2064.39576352026 | 0.924738720248957 |
| "ENSDART00000149039" | 175.371200899898 | 143.285125894702 | 0.924736899485493 |
| "ENSDART00000154155" | 287.529561155645 | 242.720816508575 | 0.924736809493114 |
| "ENSDART00000004601" | 1496.29561279946 | 1413.41032449335 | 0.924736280983691 |
| "ENSDART00000155937" | 95.8399381715383 | 72.4142855221749 | 0.924728268875276 |
| "ENSDART00000092324" | 243.482088403762 | 193.648625594739 | 0.924728213027233 |
| "ENSDART00000099503" | 266.005876629743 | 224.99057273464 | 0.92472582347102 |
| "ENSDART00000137066" | 2949.72421841993 | 2710.65268068886 | 0.92472468919024 |
| "ENSDART00000133482" | 32.0275929953468 | 23.3138930343409 | 0.924723467807579 |
| "ENSDART00000064865" | 1031.61410155292 | 964.48513593433 | 0.924723186400504 |
| "ENSDART00000122003" | 204.570017185603 | 175.62361734458 | 0.924722362077213 |
| "ENSDART00000137128" | 573.103989727977 | 523.513157898527 | 0.92471950530466 |
| "ENSDART00000085387" | 139.524764298774 | 116.99125260291 | 0.924711838454982 |
| "ENSDART00000064665" | 45.3221100121147 | 34.144973648807 | 0.924710428449683 |
| "ENSDART00000136059" | 1249.90483448855 | 1115.39732895904 | 0.924709005841739 |
| "ENSDART00000080423" | 2106.75240639222 | 1939.39237818515 | 0.924705290992372 |
| "ENSDART00000129872" | 337.927853925188 | 279.754175759158 | 0.924704814192561 |
| "ENSDART00000147699" | 295.608398233195 | 265.181349880185 | 0.924698579940111 |
| "ENSDART00000121485" | 21.0772268663542 | 11.1946342817007 | 0.924695393384398 |
| "ENSDART00000122287" | 475.851655006572 | 420.863043505669 | 0.924694398629866 |
| "ENSDART00000135065" | 4390.94193585276 | 4033.40486350992 | 0.924692891010679 |
| "ENSDART00000149064" | 15.8314360142086 | 11.1494806056025 | 0.924691821765144 |
| "ENSDART00000075495" | 9540.29380607494 | 8870.31729226214 | 0.924690732214322 |
| "ENSDART00000056921" | 508.614536526104 | 473.048137137254 | 0.924688321831349 |
| "ENSDART00000006449" | 2550.72147146838 | 2348.1357446248 | 0.924688035509937 |
| "ENSDART00000088490" | 89.4757200960025 | 70.9897542571455 | 0.924687067988602 |
| "ENSDART00000152034" | 31.4580630594499 | 22.9403044943803 | 0.924678646178002 |
| "ENSDART00000006871" | 934.372454749304 | 824.692815790685 | 0.924673799309754 |
| "ENSDART00000113100" | 20.2429520414866 | 13.9324308667192 | 0.924668455017893 |
| "ENSDART00000143343" | 33.3553010501112 | 25.7203871845511 | 0.924663615708567 |
| "ENSDART00000144224" | 41.0071540075256 | 29.1895940860421 | 0.924660338121733 |
| "ENSDART00000124755" | 83.9673950139519 | 66.9616296685357 | 0.924656147494479 |
| "ENSDART00000109551" | 15.4519950190625 | 11.7719492354839 | 0.924656015338222 |
| "ENSDART00000128975" | 710.732672931777 | 623.232923085068 | 0.924656011762583 |
| "ENSDART00000056381" | 87.9590449374855 | 68.1922323247382 | 0.924653023337731 |
| "ENSDART00000130856" | 485.86162872134 | 432.410962765317 | 0.924652581075652 |
| "ENSDART00000154761" | 449.44644961495 | 407.591551532117 | 0.924651732602034 |
| "ENSDART00000134808" | 15.4704830382613 | 10.2344136207595 | 0.924650114463639 |
| "ENSDART00000123699" | 27.8537090338087 | 17.6139636437014 | 0.924648623671449 |
| "ENSDART00000097753" | 275.824784898767 | 236.09705278073 | 0.924648258483553 |
| "ENSDART00000130304" | 14.5893689598292 | 8.99879352819402 | 0.924647397038903 |
| "ENSDART00000131005" | 46.5532250292597 | 35.3313774021702 | 0.924645002621342 |
| "ENSDART00000081214" | 664.296473455197 | 615.655306110821 | 0.924644112400259 |
| "ENSDART00000127516" | 501.177340267238 | 433.759458607012 | 0.924630942794139 |
| "ENSDART00000061698" | 341.424143821852 | 297.053311683522 | 0.924627521302272 |
| "ENSDART00000106488" | 131.423605181182 | 110.329189753642 | 0.924623129916616 |
| "ENSDART00000023638" | 209.193493213169 | 178.870884712123 | 0.924622137082371 |
| "ENSDART00000121432" | 253.739570671719 | 216.065605340834 | 0.924621388386711 |
| "ENSDART00000136072" | 68.9057269974087 | 51.7254909679239 | 0.924620857097053 |
| "ENSDART00000065356" | 534.15397448055 | 455.273328792183 | 0.92461072826258 |
| "ENSDART00000131809" | 75.6709382068471 | 60.4491253833928 | 0.924604128909885 |
| "ENSDART00000129754" | 18.2978189901238 | 11.2206762367747 | 0.92460142957947 |
| "ENSDART00000003192" | 121.037912175036 | 101.040902509163 | 0.924599436110575 |
| "ENSDART00000143086" | 23.3826090222396 | 16.4722361798068 | 0.924599038163255 |
| "ENSDART00000135769" | 44.3693710900895 | 33.5222647000264 | 0.92459660790415 |
| "ENSDART00000036499" | 145.696148234783 | 113.450613002517 | 0.92459654533204 |
| "ENSDART00000133279" | 24.1226510576207 | 16.3781064834056 | 0.9245961246871 |
| "ENSDART00000013972" | 478.36421063662 | 391.942339831471 | 0.92459381027333 |
| "ENSDART00000063559" | 23.5798820092424 | 17.3896933489662 | 0.924584962631697 |
| "ENSDART00000060004" | 724.117472143051 | 651.386073689127 | 0.924582265888912 |
| "ENSDART00000077928" | 50.3620360881559 | 39.7459929744125 | 0.924575919791176 |
| "ENSDART00000108935" | 274.382211406113 | 239.542427016094 | 0.924574934519971 |
| "ENSDART00000150351" | 62.7038471978293 | 47.080152253526 | 0.92457411484432 |
| "ENSDART00000104520" | 2507.83314325515 | 2363.19435057569 | 0.924573700419718 |
| "ENSDART00000155734" | 240.534280042321 | 207.668252672641 | 0.924570928044076 |
| "ENSDART00000148365" | 1303.54064665877 | 1203.97528451384 | 0.924563416663851 |
| "ENSDART00000105213" | 161.289985198182 | 132.490777138866 | 0.924563243775954 |
| "ENSDART00000062569" | 380.455433552868 | 343.645504813371 | 0.924557953319069 |
| "ENSDART00000086005" | 469.806308421677 | 418.180850181406 | 0.924557605356326 |
| "ENSDART00000046934" | 578.640885920383 | 515.09318391221 | 0.924557046326848 |
| "ENSDART00000112581" | 43.142375999639 | 33.1873802399125 | 0.924553300229185 |
| "ENSDART00000076946" | 296.216879504979 | 262.268433413766 | 0.924552929897963 |
| "ENSDART00000153111" | 34.6309280876184 | 23.8978979032748 | 0.92455018296942 |
| "ENSDART00000134794" | 17.7358250361914 | 12.3839056060098 | 0.924544571603336 |
| "ENSDART00000149057" | 2756.103379666 | 2582.14226409269 | 0.924540253000115 |
| "ENSDART00000035152" | 539.685303687661 | 491.508836454403 | 0.924539160607147 |
| "ENSDART00000106336" | 38.5928520488676 | 27.8694017831719 | 0.924538519162763 |
| "ENSDART00000051556" | 712.642371960616 | 642.79109216613 | 0.92453236417272 |
| "ENSDART00000050395" | 97.5002479678844 | 80.2916616508256 | 0.924531401604669 |
| "ENSDART00000138735" | 31.6888960295806 | 24.4938471914527 | 0.92453132140437 |
| "ENSDART00000144750" | 526.446503619411 | 472.197450519036 | 0.924530563713245 |
| "ENSDART00000125779" | 210.666459267541 | 184.010698072317 | 0.924528549105876 |
| "ENSDART00000077072" | 100.341600076539 | 77.8005920391418 | 0.924516561648468 |
| "ENSDART00000034563" | 42.3714861649754 | 30.6979831070163 | 0.924515744711634 |
| "ENSDART00000112232" | 62.9651760315301 | 47.7372623001501 | 0.924510528899737 |
| "ENSDART00000134633" | 128.603805211284 | 106.357448806015 | 0.924509322259717 |
| "ENSDART00000103602" | 280.388958590832 | 234.30244582401 | 0.924508032246213 |
| "ENSDART00000054410" | 207.628687632375 | 173.243820180275 | 0.924505874288104 |
| "ENSDART00000131640" | 41.7619160711233 | 32.882834214538 | 0.924504839191819 |
| "ENSDART00000074196" | 57.6597681801628 | 43.4674249233996 | 0.924502754533787 |
| "ENSDART00000103882" | 92.3860178793354 | 72.9928614939835 | 0.924500272846688 |
| "ENSDART00000005750" | 880.965544495177 | 794.781224017857 | 0.92449802124405 |
| "ENSDART00000028033" | 174.983487036432 | 141.566501440124 | 0.924497475588503 |
| "ENSDART00000127797" | 51.6942160053273 | 35.0638629778548 | 0.924494586745607 |
| "ENSDART00000112320" | 71.2039581219726 | 56.9952078587212 | 0.924493311894882 |
| "ENSDART00000003954" | 28.0287260106304 | 17.301535862187 | 0.924484937372996 |
| "ENSDART00000137801" | 267.85396140592 | 232.996002270906 | 0.924483325392328 |
| "ENSDART00000134987" | 944.388636005331 | 852.226520221827 | 0.924483069886501 |
| "ENSDART00000100126" | 18.63651595589 | 11.7356353399536 | 0.924481354697948 |
| "ENSDART00000133935" | 86.544640075199 | 68.2854072478804 | 0.924476270577402 |
| "ENSDART00000139983" | 22.7613830197284 | 15.1718733034895 | 0.924470967903286 |
| "ENSDART00000085738" | 535.326658321574 | 412.020435782052 | 0.924469150031364 |
| "ENSDART00000154628" | 96.1080991157789 | 76.0129844935073 | 0.924465083972736 |
| "ENSDART00000007058" | 573.156257606293 | 518.249406715014 | 0.924455935266283 |
| "ENSDART00000155830" | 273.399999412724 | 229.717444946533 | 0.924454187318891 |
| "ENSDART00000156723" | 28.300303010141 | 21.7101792248073 | 0.924454166563944 |
| "ENSDART00000131651" | 19.6964150021261 | 13.6739965418708 | 0.924453819148202 |
| "ENSDART00000147879" | 102.777454173953 | 84.2710505380316 | 0.924451589942111 |
| "ENSDART00000066590" | 61.0442082579772 | 44.0468897426 | 0.924447109540213 |
| "ENSDART00000052730" | 5964.24751641612 | 5527.16984400358 | 0.924446742023145 |
| "ENSDART00000152161" | 130.477027513202 | 102.035764586847 | 0.924442610593767 |
| "ENSDART00000154425" | 24.1596270960184 | 17.3896933489662 | 0.924439425096318 |
| "ENSDART00000013654" | 145.206131060156 | 115.012755161258 | 0.924435194314714 |
| "ENSDART00000148456" | 1287.02013425981 | 1190.25455437869 | 0.924433870547297 |
| "ENSDART00000141049" | 240.655858803312 | 201.193494442917 | 0.924433767605141 |
| "ENSDART00000109037" | 493.15539047572 | 434.028879326677 | 0.924432698451746 |
| "ENSDART00000147513" | 59.4762219834889 | 45.8555218069455 | 0.924431759025105 |
| "ENSDART00000112243" | 155.059655119446 | 131.137922142807 | 0.924425093675859 |
| "ENSDART00000126426" | 286.910244420763 | 250.222039519917 | 0.924421264403603 |
| "ENSDART00000134907" | 20.6371130648493 | 13.0106739667255 | 0.924414475793001 |
| "ENSDART00000079161" | 53.4602121531737 | 40.2528300025523 | 0.924413539132766 |
| "ENSDART00000142818" | 370.919857236805 | 337.3885705333 | 0.924410851097055 |
| "ENSDART00000111006" | 66.354120986682 | 53.3275322986698 | 0.924409996031075 |
| "ENSDART00000098643" | 149.271447154634 | 128.080767542224 | 0.924409525402622 |
| "ENSDART00000154918" | 16.2071090183725 | 11.7841339736271 | 0.92440864277631 |
| "ENSDART00000109304" | 425.899241113413 | 386.685584807875 | 0.924405053115831 |
| "ENSDART00000033132" | 363.243036085364 | 315.174830060892 | 0.924399888648953 |
| "ENSDART00000079930" | 622.006057383408 | 572.663553608128 | 0.92439912038492 |
| "ENSDART00000136041" | 161.844795105863 | 137.896913082386 | 0.924398326387659 |
| "ENSDART00000148950" | 399.942737055335 | 344.111175149143 | 0.924396596010358 |
| "ENSDART00000114576" | 73.2105008660494 | 55.0019611015772 | 0.924396244373623 |
| "ENSDART00000111084" | 84.9118280527268 | 69.1868573346918 | 0.924394818330184 |
| "ENSDART00000015136" | 140.933007907015 | 116.3319894397 | 0.924394671982986 |
| "ENSDART00000039345" | 744.205100623119 | 644.731362288178 | 0.924380846622281 |
| "ENSDART00000149412" | 1310.13423023255 | 1142.06946206587 | 0.924379718473757 |
| "ENSDART00000020261" | 427.098921033987 | 369.382382969238 | 0.924372498539819 |
| "ENSDART00000122403" | 939.106476154724 | 863.136622051245 | 0.924371556340169 |
| "ENSDART00000014240" | 260.304866997233 | 221.530027969516 | 0.924370835946768 |
| "ENSDART00000109324" | 52.0993290659244 | 35.9657904512956 | 0.924370116768408 |
| "ENSDART00000133000" | 78.8229879826144 | 64.4884799368868 | 0.924365978140352 |
| "ENSDART00000139174" | 184.859970977327 | 155.616950841732 | 0.924360322881348 |
| "ENSDART00000132934" | 16.7502630173937 | 8.99712104940635 | 0.924354464188945 |
| "ENSDART00000156174" | 203.083200049092 | 171.748330351803 | 0.924353854257594 |
| "ENSDART00000146485" | 1828.47121626308 | 1527.41995389664 | 0.924352599361481 |
| "ENSDART00000153242" | 89.0770872102328 | 67.7154339889094 | 0.924350003263356 |
| "ENSDART00000112782" | 192.375791308738 | 163.102100844859 | 0.92434411325377 |
| "ENSDART00000113062" | 85.6601759713582 | 67.9263903792104 | 0.924339895042412 |
| "ENSDART00000151476" | 75.5860340928173 | 61.7064844490291 | 0.924335660997706 |
| "ENSDART00000111936" | 692.582355615256 | 552.02230941663 | 0.924326114780812 |
| "ENSDART00000108780" | 45.217662071749 | 35.6602896526622 | 0.924324250305543 |
| "ENSDART00000024743" | 121.58141810977 | 99.4782797126235 | 0.924323814471117 |
| "ENSDART00000074639" | 54.8848321663393 | 42.4981241629914 | 0.924323125117661 |
| "ENSDART00000133906" | 15.5115790033536 | 11.4803056537813 | 0.924308797800064 |
| "ENSDART00000046172" | 7847.41296620498 | 6093.52265054779 | 0.924308206560827 |
| "ENSDART00000113581" | 231.266596293992 | 203.521454518312 | 0.924303371306453 |
| "ENSDART00000042467" | 92.0099929394592 | 75.0271992641216 | 0.924299762685074 |
| "ENSDART00000124284" | 28.404047079082 | 20.1535318890587 | 0.924297519299424 |
| "ENSDART00000130581" | 454.392076871643 | 405.360829042968 | 0.924296686191487 |
| "ENSDART00000140852" | 268.610214771573 | 222.987774993737 | 0.924294761617735 |
| "ENSDART00000011078" | 94.0249911558533 | 70.5129559213167 | 0.924292900763227 |
| "ENSDART00000075928" | 836.776634492248 | 791.927022579595 | 0.924292573696143 |
| "ENSDART00000148460" | 951.047274387847 | 864.819716241688 | 0.924288981940971 |
| "ENSDART00000113285" | 175.530409026436 | 152.586559152921 | 0.924284286956864 |
| "ENSDART00000074272" | 26.607873988447 | 18.6300905589955 | 0.924283136960209 |
| "ENSDART00000143352" | 31.6149439527852 | 23.5802123664982 | 0.924279340795942 |
| "ENSDART00000143924" | 141.382314067192 | 121.228598428335 | 0.924279190935275 |
| "ENSDART00000004357" | 118.946179411079 | 82.1858898757708 | 0.924278336800958 |
| "ENSDART00000127318" | 419.254986308987 | 378.320726942177 | 0.924275923259255 |
| "ENSDART00000129276" | 846.090552667508 | 786.714169967578 | 0.92426773985364 |
| "ENSDART00000052383" | 9193.24161609184 | 8511.55049664432 | 0.924267444259153 |
| "ENSDART00000143551" | 92.5092067298204 | 63.4098227779564 | 0.924263401917778 |
| "ENSDART00000036513" | 601.792426610916 | 504.369619326784 | 0.924260315906756 |
| "ENSDART00000012522" | 439.429344611508 | 385.027630720438 | 0.924257712286377 |
| "ENSDART00000102555" | 71.9341369421869 | 56.6930520176631 | 0.924255905462195 |
| "ENSDART00000056763" | 84.9042920707623 | 70.3675259461633 | 0.924250342574732 |
| "ENSDART00000122854" | 190.382158370041 | 144.504268020628 | 0.924249961404587 |
| "ENSDART00000130781" | 1131.40829564402 | 1056.3554667403 | 0.924246984083897 |
| "ENSDART00000024576" | 669.549129432814 | 616.444229234567 | 0.924241747562388 |
| "ENSDART00000122592" | 114.487758100375 | 95.879409599428 | 0.924240025804621 |
| "ENSDART00000048366" | 100.145063975891 | 81.5533204472962 | 0.924234304346058 |
| "ENSDART00000011757" | 265.460226065804 | 215.612211954525 | 0.924232485714213 |
| "ENSDART00000114703" | 18.279330970925 | 12.4383764493053 | 0.924229145655983 |
| "ENSDART00000104740" | 236.429744235449 | 204.823249554517 | 0.924229129975406 |
| "ENSDART00000137096" | 62.2364049541652 | 48.44119850166 | 0.924229080359113 |
| "ENSDART00000018981" | 671.760597719994 | 625.831081009185 | 0.924228795680643 |
| "ENSDART00000146022" | 330.024451790034 | 280.349812300232 | 0.924225958674994 |
| "ENSDART00000124772" | 74.9908651064001 | 60.184238211124 | 0.924215962615279 |
| "ENSDART00000126009" | 261.392897401844 | 216.512947586977 | 0.924214426140812 |
| "ENSDART00000145138" | 111.684367550332 | 84.0766445425752 | 0.924211500423003 |
| "ENSDART00000079968" | 204.057425080013 | 171.930143399523 | 0.924210952808262 |
| "ENSDART00000053797" | 2941.82794435041 | 2376.83434835336 | 0.924210586374381 |
| "ENSDART00000017467" | 653.539174614004 | 514.932990990502 | 0.924207234044069 |
| "ENSDART00000099869" | 1102.02267015605 | 1015.89125263084 | 0.924206514335928 |
| "ENSDART00000146700" | 261.352791214028 | 233.498058930413 | 0.924196687985425 |
| "ENSDART00000132315" | 16.8618950040115 | 12.7350374673709 | 0.924193606829904 |
| "ENSDART00000114885" | 259.739589047753 | 223.939455616539 | 0.924190106785317 |
| "ENSDART00000102051" | 34.9364170059691 | 26.0103582874661 | 0.924184077599221 |
| "ENSDART00000125997" | 50.4627160375393 | 38.195317848286 | 0.924181230924292 |
| "ENSDART00000146173" | 3576.68325422469 | 3305.07304577663 | 0.92417621802038 |
| "ENSDART00000128122" | 475.951734386385 | 430.516500723811 | 0.924172348066837 |
| "ENSDART00000056540" | 643.040540923183 | 553.136342229778 | 0.924171683956899 |
| "ENSDART00000134647" | 1507.57110850462 | 1384.05669238321 | 0.924169497044429 |
| "ENSDART00000123384" | 501.608277195656 | 446.247186498423 | 0.924168452935067 |
| "ENSDART00000139102" | 824.628477967662 | 770.236429211272 | 0.924167902239353 |
| "ENSDART00000112457" | 73.815566082564 | 57.6477778556117 | 0.924167512475733 |
| "ENSDART00000023745" | 25.4288069736287 | 18.9300965346364 | 0.924166967475959 |
| "ENSDART00000131527" | 17.6207769943038 | 12.7218979559688 | 0.924165891641746 |
| "ENSDART00000123540" | 20.0385280231622 | 14.8802297217869 | 0.924165200026482 |
| "ENSDART00000149237" | 796.201136600606 | 720.03504110658 | 0.924161215726237 |
| "ENSDART00000039865" | 825.943225672771 | 777.264613556292 | 0.924155126003646 |
| "ENSDART00000077550" | 357.131704643495 | 285.20450410318 | 0.924149794044667 |
| "ENSDART00000112124" | 365.09443787202 | 321.146813925581 | 0.924146693631786 |
| "ENSDART00000029206" | 21.6456679801834 | 14.5608717062226 | 0.92414667988387 |
| "ENSDART00000077761" | 1077.51862033514 | 968.379982147853 | 0.924143381240847 |
| "ENSDART00000114689" | 71.2468797954878 | 53.3339818949213 | 0.92414102955801 |
| "ENSDART00000136841" | 24.8223009993341 | 17.3686688302552 | 0.92413998053382 |
| "ENSDART00000049651" | 999.24719349289 | 945.763536428642 | 0.924139461815075 |
| "ENSDART00000144845" | 56.2618760395852 | 40.901577655238 | 0.924134396472758 |
| "ENSDART00000113712" | 105.690046175558 | 88.6756312375479 | 0.92413210495057 |
| "ENSDART00000100948" | 38.4216030630281 | 28.1653450957089 | 0.924128744446746 |
| "ENSDART00000156883" | 20.2093920583588 | 14.8713899412191 | 0.924127930270607 |
| "ENSDART00000024919" | 115.987419012404 | 94.7839649779226 | 0.924110090392704 |
| "ENSDART00000010496" | 141.094191331042 | 112.025294854922 | 0.924109154571358 |
| "ENSDART00000008854" | 2756.74675616354 | 2606.07035232312 | 0.924104605011671 |
| "ENSDART00000124540" | 731.340348153496 | 673.056014709372 | 0.924102892435358 |
| "ENSDART00000067803" | 10977.2893554474 | 10206.0755389207 | 0.924100217727787 |
| "ENSDART00000135655" | 164.769043016126 | 138.918294501773 | 0.92409197615174 |
| "ENSDART00000142717" | 253.528952384419 | 212.282051357803 | 0.924090502865764 |
| "ENSDART00000137431" | 17.2933840014843 | 11.7874789312024 | 0.924083800200181 |
| "ENSDART00000061497" | 402.477610468363 | 353.822760915259 | 0.924080497913337 |
| "ENSDART00000082250" | 12070.0471833449 | 11271.4557305303 | 0.924072393971178 |
| "ENSDART00000017695" | 101.117496313319 | 81.2463874887743 | 0.924072286033206 |
| "ENSDART00000145894" | 32.9467719342442 | 22.8779486437759 | 0.924068455519618 |
| "ENSDART00000127022" | 264.435158428761 | 224.242980942787 | 0.92406705823974 |
| "ENSDART00000121550" | 909.631624279385 | 841.379988842185 | 0.924063932502829 |
| "ENSDART00000123222" | 184.93698717368 | 159.362755518175 | 0.924062744902655 |
| "ENSDART00000149532" | 800.770672887562 | 706.463704545743 | 0.924061214311828 |
| "ENSDART00000017838" | 556.133630158758 | 512.216271845386 | 0.924056339942303 |
| "ENSDART00000137706" | 30.5656450080711 | 23.8747235191466 | 0.924045015454551 |
| "ENSDART00000132153" | 18.316658945035 | 12.3839056060098 | 0.9240449120086 |
| "ENSDART00000122237" | 66.9633391448218 | 51.5719356665625 | 0.924044688556981 |
| "ENSDART00000043173" | 6717.25556868399 | 6236.93768344682 | 0.924034398130736 |
| "ENSDART00000157190" | 16.7838230005215 | 12.402062553775 | 0.924020631087745 |
| "ENSDART00000124367" | 18.6847959672344 | 13.0515279119894 | 0.924018269454735 |
| "ENSDART00000149768" | 279.197316177002 | 243.312057856326 | 0.924015375092776 |
| "ENSDART00000040229" | 48.7397911522751 | 37.232229616399 | 0.923996075531989 |
| "ENSDART00000074592" | 1788.40476014874 | 1530.7767030121 | 0.923996045125844 |
| "ENSDART00000036546" | 33.1546120078385 | 25.4535904657644 | 0.923984060932829 |
| "ENSDART00000077119" | 494.856076880147 | 437.611169508406 | 0.92397819183342 |
| "ENSDART00000054982" | 71.3943989984356 | 58.5955767106794 | 0.923977990311278 |
| "ENSDART00000083388" | 84.5957720477846 | 69.8339325085898 | 0.923977172757186 |
| "ENSDART00000108869" | 308.985107180147 | 276.148846296205 | 0.92397661807373 |
| "ENSDART00000145979" | 279.528527705079 | 242.177975080841 | 0.923976562295528 |
| "ENSDART00000155269" | 74.5891681010729 | 59.3085930116564 | 0.923958603045909 |
| "ENSDART00000076478" | 1240.98608464327 | 959.928362916304 | 0.923958537962061 |
| "ENSDART00000151482" | 20.74913000211 | 15.8113037889779 | 0.923951762920858 |
| "ENSDART00000150578" | 18.5656279986522 | 11.7382625920002 | 0.923951201659089 |
| "ENSDART00000084282" | 785.831520821252 | 723.905310662994 | 0.923949766655016 |
| "ENSDART00000078336" | 42.795791116196 | 33.506975323207 | 0.923945655756743 |
| "ENSDART00000149327" | 16.1437570430991 | 10.8497149488607 | 0.923944636274258 |
| "ENSDART00000152739" | 23.6692579856791 | 16.0957800689004 | 0.923939733000361 |
| "ENSDART00000109703" | 670.997327426501 | 597.047906046987 | 0.92392763054367 |
| "ENSDART00000041590" | 19.1419570301582 | 13.9539327720597 | 0.923925638463631 |
| "ENSDART00000151262" | 17.6207769943038 | 13.343171493692 | 0.923915799488695 |
| "ENSDART00000125935" | 205.448249748475 | 170.143184382381 | 0.923911870548839 |
| "ENSDART00000135826" | 19.4991420151232 | 14.2670782591028 | 0.923903722379823 |
| "ENSDART00000113087" | 85.2833811301961 | 67.9502792176984 | 0.923901161639359 |
| "ENSDART00000126587" | 92.3065051180654 | 70.8057945951775 | 0.92389828607097 |
| "ENSDART00000026273" | 1551.75775458981 | 1372.52461231522 | 0.92389751304493 |
| "ENSDART00000135562" | 90.1308910322844 | 70.7057586150215 | 0.923892857990074 |
| "ENSDART00000137717" | 348.911410572251 | 301.317894556179 | 0.923890143078104 |
| "ENSDART00000042678" | 90.3092910494454 | 72.2351689035379 | 0.923887442042942 |
| "ENSDART00000077980" | 119.66910815518 | 97.0447888340803 | 0.923886551572464 |
| "ENSDART00000023931" | 81735.9144917904 | 78197.5388132021 | 0.923884423275252 |
| "ENSDART00000125680" | 488.145405980891 | 442.126295196264 | 0.923879687466264 |
| "ENSDART00000156936" | 32.7751710126923 | 25.1206155521684 | 0.923879420181258 |
| "ENSDART00000073706" | 77.2527910490603 | 64.238645073707 | 0.92387750100601 |
| "ENSDART00000065278" | 92.0688730523256 | 71.557274657102 | 0.923874181343336 |
| "ENSDART00000042492" | 101.264547006418 | 83.1661143562968 | 0.923871589841317 |
| "ENSDART00000086290" | 576.494140079333 | 516.294801363019 | 0.92386949001027 |
| "ENSDART00000111981" | 35.159362058423 | 21.1197247596219 | 0.923863474105358 |
| "ENSDART00000080863" | 94.3070692120941 | 75.2951910750665 | 0.923858029361232 |
| "ENSDART00000014031" | 103.078053264323 | 82.1296806278204 | 0.923857624514768 |
| "ENSDART00000007819" | 617.987679587287 | 572.933451714422 | 0.923851844501839 |
| "ENSDART00000099847" | 328.371176450873 | 286.851155797527 | 0.923850854166418 |
| "ENSDART00000152071" | 88.3970141097858 | 71.8957443936904 | 0.923846633328494 |
| "ENSDART00000136772" | 265.457410580106 | 221.703346771563 | 0.923839421927723 |
| "ENSDART00000135669" | 193.456505607376 | 156.903222684557 | 0.923832346946921 |
| "ENSDART00000016758" | 545.507474115588 | 490.214929676675 | 0.923827920329129 |
| "ENSDART00000020550" | 1043.70225612764 | 989.108814228181 | 0.923818562578743 |
| "ENSDART00000145893" | 88.5493901257835 | 67.2126563727048 | 0.923809731300425 |

**Table S6**. Full list of GO terms enriched in up-regulated genes after NaBu treatment, ranked by their *P*-values.

| **Term** | **P-Value** |
| --- | --- |
| ribonucleoprotein complex biogenesis | 3,50E-05 |
| ribosome biogenesis | 2,70E-04 |
| enzyme activator activity | 7,60E-04 |
| ncRNA metabolic process | 1,00E-03 |
| nuclear lumen | 1,40E-03 |
| lipid modification | 1,60E-03 |
| intracellular organelle lumen | 1,90E-03 |
| organelle lumen | 1,90E-03 |
| GTPase activator activity | 2,20E-03 |
| ion channel complex | 2,30E-03 |
| membrane-enclosed lumen | 3,00E-03 |
| embryonic cranial skeleton morphogenesis | 3,50E-03 |
| embryonic skeletal system development | 3,50E-03 |
| embryonic skeletal system morphogenesis | 3,50E-03 |
| cation transport | 3,90E-03 |
| nucleolus | 4,50E-03 |
| ion transport | 5,30E-03 |
| rRNA processing | 5,40E-03 |
| intracellular signaling cascade | 5,80E-03 |
| cell fraction | 6,60E-03 |
| rRNA metabolic process | 6,80E-03 |
| cation channel complex | 7,40E-03 |
| zinc ion binding | 8,40E-03 |
| skeletal system morphogenesis | 9,30E-03 |
| transcription factor binding | 1,00E-02 |
| regulation of cellular component biogenesis | 1,20E-02 |
| protein transport | 1,20E-02 |
| establishment of protein localization | 1,20E-02 |
| regulation of cell proliferation | 1,20E-02 |
| phospholipase C activity | 1,30E-02 |
| transferase activity, transferring pentosyl groups | 1,30E-02 |
| monovalent inorganic cation transport | 1,40E-02 |
| synaptic vesicle | 1,50E-02 |
| protein localization | 1,60E-02 |
| nuclear pore | 1,60E-02 |
| pore complex | 1,60E-02 |
| negative regulation of cell proliferation | 1,60E-02 |
| ncRNA processing | 1,80E-02 |
| regulation of small GTPase mediated signal transduction | 1,90E-02 |
| nuclear envelope | 1,90E-02 |
| insoluble fraction | 2,00E-02 |
| metal ion transport | 2,10E-02 |
| phospholipid metabolic process | 2,10E-02 |
| tRNA metabolic process | 2,20E-02 |
| regulation of transcription | 2,40E-02 |
| RNA processing | 2,60E-02 |
| chordate embryonic development | 2,60E-02 |
| embryonic development ending in birth or egg hatching | 2,60E-02 |
| cellular macromolecular complex assembly | 2,70E-02 |
| sodium ion transport | 2,70E-02 |
| cytosol | 3,30E-02 |
| tRNA aminoacylation for protein translation | 3,40E-02 |
| embryonic viscerocranium morphogenesis | 3,50E-02 |
| small nucleolar ribonucleoprotein complex | 3,60E-02 |
| cellular macromolecular complex subunit organization | 3,60E-02 |
| metal ion transmembrane transporter activity | 3,60E-02 |
| endopeptidase inhibitor activity | 3,70E-02 |
| tRNA aminoacylation | 3,90E-02 |
| amino acid activation | 3,90E-02 |
| inorganic anion transport | 3,90E-02 |
| oligodendrocyte cell fate commitment | 4,00E-02 |
| regulation of cell-substrate adhesion | 4,00E-02 |
| lifelong otolith mineralization | 4,00E-02 |
| regulation of cell-matrix adhesion | 4,00E-02 |
| small GTPase mediated signal transduction | 4,30E-02 |
| voltage-gated cation channel activity | 4,40E-02 |
| mitotic cell cycle | 4,50E-02 |
| lipid catabolic process | 4,60E-02 |
| membrane fraction | 4,60E-02 |
| phosphoinositide metabolic process | 4,60E-02 |
| voltage-gated ion channel activity | 4,90E-02 |
| embryonic morphogenesis | 4,90E-02 |
| synapse part | 5,00E-02 |
| hydro-lyase activity | 5,00E-02 |

**Table S7**. Full list of GO terms enriched in down-regulated genes after NaBu treatment, ranked by their *P*-values.

| **Term** | ***P*-Value** |
| --- | --- |
| structural molecule activity | 3,80E-20 |
| structural constituent of ribosome | 5,90E-10 |
| calcium ion binding | 2,50E-08 |
| oxidation reduction | 6,50E-08 |
| translation | 1,80E-07 |
| cofactor binding | 2,10E-07 |
| generation of precursor metabolites and energy | 2,60E-07 |
| visual perception | 4,30E-07 |
| sensory perception of light stimulus | 4,30E-07 |
| striated muscle cell development | 1,80E-06 |
| myofibril assembly | 3,80E-06 |
| muscle cell development | 4,50E-06 |
| coenzyme binding | 5,60E-06 |
| sensory perception | 5,90E-06 |
| cognition | 5,90E-06 |
| cellular component assembly involved in morphogenesis | 7,20E-06 |
| acetyl-CoA metabolic process | 7,30E-06 |
| striated muscle cell differentiation | 8,80E-06 |
| hexose catabolic process | 1,30E-05 |
| glucose catabolic process | 1,30E-05 |
| monosaccharide catabolic process | 1,30E-05 |
| electron carrier activity | 1,30E-05 |
| actin binding | 1,50E-05 |
| neurological system process | 1,70E-05 |
| alcohol catabolic process | 2,20E-05 |
| cellular carbohydrate catabolic process | 2,20E-05 |
| muscle cell differentiation | 2,50E-05 |
| glucose metabolic process | 2,70E-05 |
| tricarboxylic acid cycle | 2,90E-05 |
| acetyl-CoA catabolic process | 2,90E-05 |
| actomyosin structure organization | 3,50E-05 |
| cytoskeletal protein binding | 3,80E-05 |
| muscle organ development | 4,00E-05 |
| aerobic respiration | 6,60E-05 |
| coenzyme catabolic process | 6,60E-05 |
| monosaccharide metabolic process | 1,20E-04 |
| glycolysis | 1,30E-04 |
| detection of light stimulus | 1,40E-04 |
| cytoskeleton organization | 1,50E-04 |
| skeletal muscle organ development | 2,30E-04 |
| antioxidant activity | 2,90E-04 |
| hexose metabolic process | 3,20E-04 |
| striated muscle contraction | 3,30E-04 |
| coenzyme metabolic process | 4,00E-04 |
| carbohydrate catabolic process | 4,20E-04 |
| hydrolase activity, acting on acid anhydrides, catalyzing transmembrane movement of substances | 4,70E-04 |
| calcium-dependent cysteine-type endopeptidase activity | 5,60E-04 |
| cellular respiration | 6,10E-04 |
| energy derivation by oxidation of organic compounds | 7,70E-04 |
| translational elongation | 8,50E-04 |
| transmembrane transport | 9,30E-04 |
| calcium-dependent phospholipid binding | 1,00E-03 |
| acyl-CoA dehydrogenase activity | 1,00E-03 |
